# Intermittent ischemia/reperfusion as a potent insulin-sensitizing intervention via blood flow enhancement and muscle decanoyl-l-carnitine suppression

**DOI:** 10.1172/JCI183567

**Published:** 2025-09-02

**Authors:** Kohei Kido, Janne R. Hingst, Johan Onslev, Kim A. Sjøberg, Jesper B. Birk, Nicolas O. Eskesen, Tongzhu Zhou, Kentaro Kawanaka, Jesper F. Havelund, Nils J. Færgeman, Ylva Hellsten, Jørgen F.P. Wojtaszewski, Rasmus Kjøbsted

**Affiliations:** 1 The August Krogh Section for Human and Molecular Physiology, Department of Nutrition, Exercise and Sports, Faculty of Science, University of Copenhagen, Copenhagen, Denmark.; 2 Health and Medical Research Institute, National Institute of Advanced Industrial Science and Technology (AIST), Takamatsu, Kagawa, Japan.; 3 Faculty of Sports and Health Science, Fukuoka University, Fukuoka, Japan.; 4 Department of Biochemistry and Molecular Biology, VILLUM Center for Bioanalytical Sciences, University of Southern Denmark, Odense, Denmark.

**Keywords:** Metabolism, Muscle biology, Glucose metabolism, Insulin, Skeletal muscle

## Abstract

A single bout of exercise improves muscle insulin sensitivity for up to 48 hours via AMPK. Limb ischemia activates AMPK in muscle, and subsequent reperfusion enhances insulin-stimulated vasodilation, potentially eliciting a more pronounced exercise effect with reduced workload. We investigated the combined effect of upper leg intermittent ischemia/reperfusion (IIR) and continuous knee-extension exercise on muscle insulin sensitivity regulation. We found that IIR exercise potentiated AMPK activation and muscle insulin sensitivity. The potentiating effect of IIR exercise on muscle insulin sensitivity was associated with increased insulin-stimulated blood flow in parallel with enhanced phosphorylation of endothelial nitric oxide synthase. Metabolomics analyses demonstrated a suppression of muscle medium-chain acylcarnitines during IIR exercise, which correlated with insulin sensitivity and was consistent with findings in isolated rat muscle treated with decanoyl-l-carnitine. Collectively, combining IIR with low- to moderate-intensity exercise may represent a promising intervention to effectively enhance muscle insulin sensitivity. This approach could offer potential for mitigating muscle insulin resistance in clinical settings and among individuals with lower physical activity levels.

## Abstract 2

Combining intermittent ischemia-reperfusion with knee-extension exercise improves insulin sensitivity via increased blood flow and reduced muscle Decanoyl-L-carnitine, offering clinical potential.

## Graphical abstract

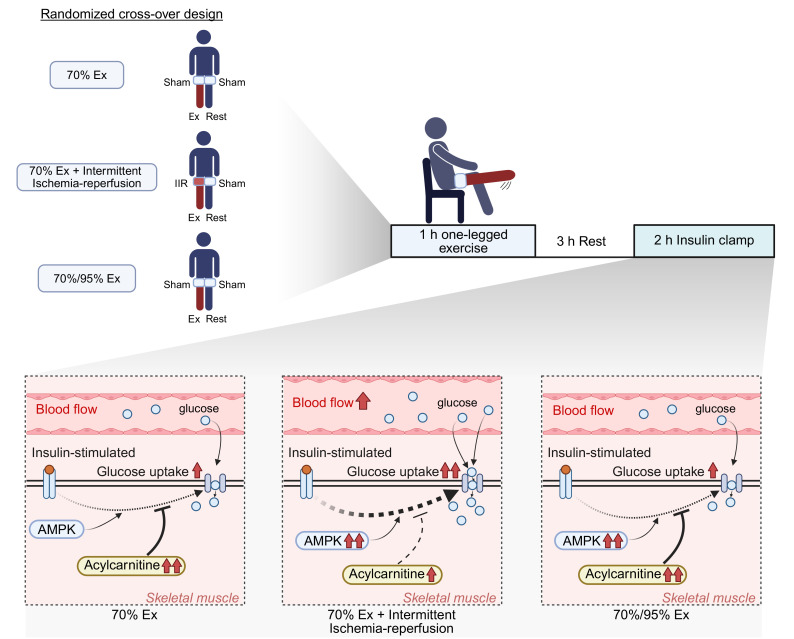

## Introduction

Skeletal muscle accounts for up to 80% of insulin-stimulated whole-body glucose disposal (1, 2). Illuminating ways of enhancing skeletal muscle insulin sensitivity has thus gained much attention, in part due to the rapidly increasing prevalence of obesity and type 2 diabetes worldwide. A single bout of exercise increases the effect of insulin on skeletal muscle glucose uptake in humans (3–5). This effect has been reported to be sustained for up to 48 hours (6). The insulin-sensitizing effect of exercise involves a coordinated increase in microvascular perfusion (7) and intramuscular events (8–10), such as GLUT4 recruitment to the cell surface membrane (11). Recently, we showed that activation of the AMPK-TBC1D4 signaling axis in skeletal muscle following exercise is necessary to enhance insulin sensitivity (8–10).

Short-term limb ischemia reduces glycogen, phosphocreatine, and ATP levels in skeletal muscle, whereas ADP and AMP levels increase (12, 13). Because an increase in the intracellular AMP/ATP ratio activates AMPK, ischemia has the potential to activate AMPK in skeletal muscle. Indeed, hindlimb vascular occlusion and common iliac artery ischemia have been shown to increase phosphorylation of AMPKα Thr^172^ in rodent skeletal muscle (14, 15). In humans, blood flow restriction of the leg augments exercise-induced phosphorylation of the AMPK downstream target ACC Ser^221^ (16). Collectively, these findings suggest that short-term limb ischemia enhances AMPK activation in skeletal muscle during exercise, which may improve muscle insulin sensitivity in the period after exercise. In addition, reperfusion following ischemia produces endothelial shear stress that enhances vasodilation in response to insulin in mammals, including humans (17).

Based on these insights, we hypothesize that upper-leg intermittent ischemia/reperfusion (IIR) during exercise potentiates the insulin-sensitizing effect of exercise on muscle glucose uptake. This potentiation may occur through further activation of AMPK and/or improved blood flow (enhanced glucose delivery). To test this hypothesis, we investigated the effect of IIR on muscle insulin sensitivity during moderate-intensity continuous knee-extension exercise. We used the hyperinsulinemic-euglycemic clamp to assess insulin-stimulated whole-body and limb glucose uptake, which serves as our primary measures of insulin sensitization. Additionally, guided by a prior pilot experiment, we included a high-intensity interval exercise trial that produced a similar magnitude of AMPK activation and glycogen consumption in skeletal muscle compared with the IIR-exercise trial. Our findings offer compelling evidence that IIR potentiates the insulin-sensitizing effect of exercise on muscle glucose uptake by increasing blood flow, rather than solely relying on AMPK activation. Supported by deep metabolomics analyses as well as animal and cell culture–based experiments, we identify the suppression of decanoyl-l-carnitine by IIR as a probable mechanism contributing to the potentiation of muscle insulin sensitivity.

## Results

### IIR potentiates the insulin-sensitizing effect of exercise.

**Figure 1:**
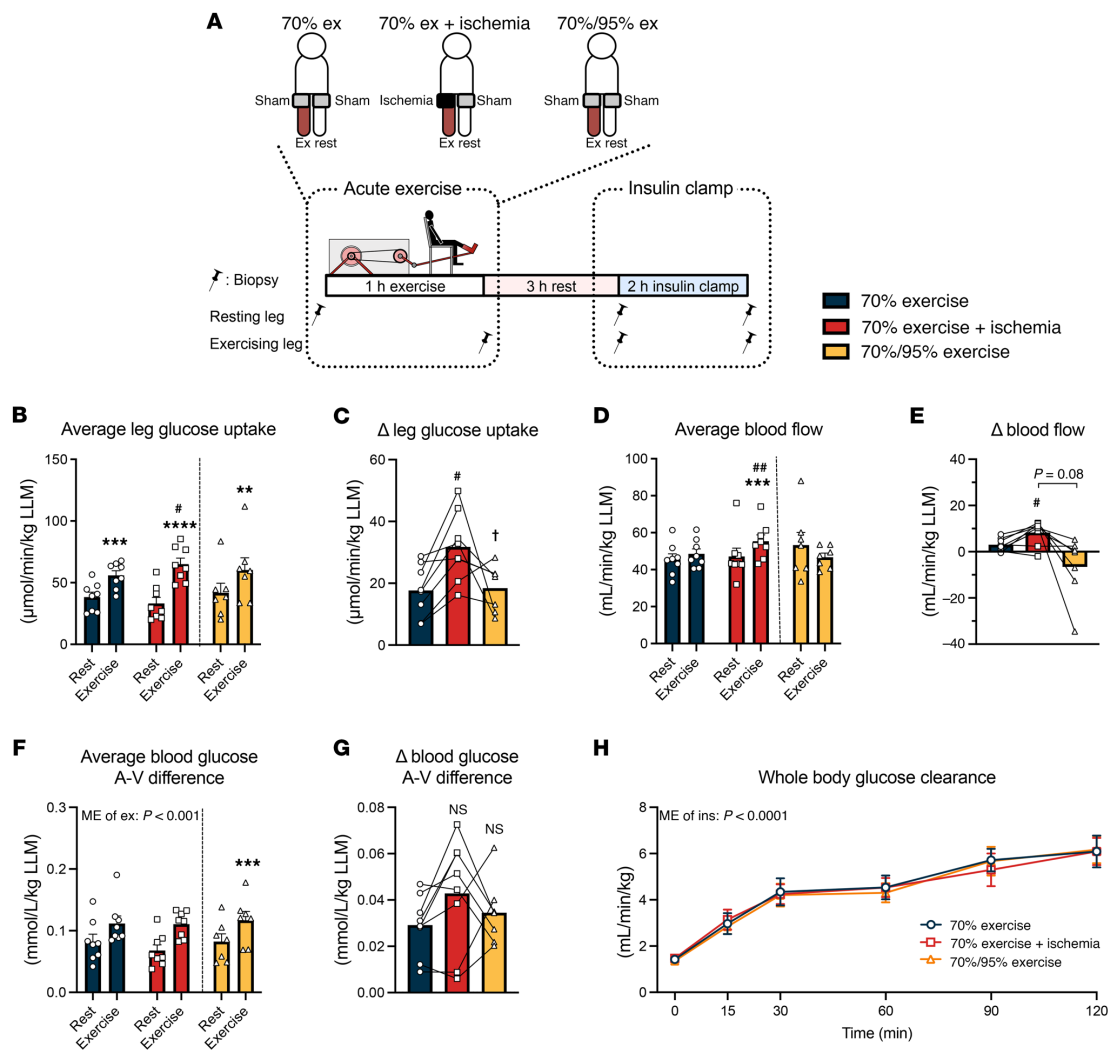
IIR potentiates the insulin-sensitizing effect of a single bout of exercise. (**A**) Schematic of the human study. Participants performed a 1-legged knee extension exercise under 1 of the following regimens in a randomized crossover design: 70%EX, 70%EX + ischemia, or 70%/95%EX. After a 3-hour rest, a 2-hour hyperinsulinemic-euglycemic clamp was performed, with biopsy and blood sampling collected from rested and exercised legs. (**B**–**H**) Results from the insulin clamp: average leg glucose uptake (**B**), prior exercise-induced change in leg glucose uptake (Δ) (**C**), average leg blood flow (**D**), prior exercise-induced change in blood flow (Δ) (**E**), average blood glucose A-V difference (**F**), prior exercise-induced change in A–V glucose difference (Δ) (**G**), and whole-body glucose clearance (**H**). Sample sizes were as follows: *n* = 8 for 70%EX and 70%EX + ischemia; *n* = 7 for 70%/95%EX. Data are presented as means ± SEM. (**B**, **D**, and **F**) Comparisons between 70%EX and 70%EX + ischemia were made with 2-way repeated-measures ANOVA; significant interactions were followed by Bonferroni-Šidák post hoc tests. For 70%/95%EX, the exercise effect was evaluated using a paired (2-tailed) Student’s *t* test. (**C**, **E**, and **G**) One-way repeated-measures ANOVA was used, followed by Tukey’s post hoc test when significant. (**H**) Analysis was conducted using 2-way repeated-measures ANOVA to assess main effects. **P < 0.01, ***P < 0.001, ****P < 0.0001 vs. rest within trial; ^#^*P* < 0.05, ^##^*P* < 0.01 vs. 70%EX within rest or exercise leg; ^†^*P* < 0.05 vs. 70%EX + ischemia. Δ, change in; Ex, exercise; Ins, insulin; ME, main effect; LLM, lean leg mass.

To investigate the insulin-sensitizing effect of IIR on muscle glucose uptake, each participant underwent a euglycemic-hyperinsulinemic clamp 3 hours into recovery from 3 knee-extension exercise trials: (a) continuous moderate-intensity exercise (70%EX), (b) continuous moderate-intensity exercise with IIR (IIR-70%EX) and (c) high-intensity interval exercise (70%/95%EX) (Figure 1A). Insulin-stimulated muscle glucose uptake was enhanced by prior 70%EX (52.7% ± 11.6%) and significantly potentiated when combined with IIR (111.8% ± 18.6%) (Figure 1, B and C). The enhancing effect of 70%/95%EX on insulin-stimulated muscle glucose uptake (47.2% ± 6.2%) did not differ from that induced by 70%EX (Figure 1, B and C). Femoral artery blood flow during the insulin clamp was significantly increased by prior IIR-70%EX (19.8% ± 4.4%) compared with 70%EX (7.3% ± 2.7%) and 70%/95%EX (–7.2% ± 7.0%) (Figure 1, D and E). The blood glucose arteriovenous (A-V) difference was elevated in the prior exercised leg compared with the rested leg during the insulin clamp but was similar among the 3 exercise trials (Figure 1, F and G). Whole-body glucose clearance during the insulin clamp did not differ among the 3 exercise trials (Figure 1H). Plasma levels of fatty acids (FAs), triacylglycerol (TG), and glycerol decreased, whereas insulin levels increased during the insulin clamp but no differences were observed among the 3 exercise trials (Table 1). Plasma adrenaline and noradrenalin levels did not differ among the 3 groups at the time of insulin clamp initiation (Supplemental Figure 2, I and J; supplemental material available online with this article; https://www.jci.org/articles/view/183567/sd/3). Collectively, these findings suggest that IIR potentiates the insulin-sensitizing effect of exercise by enhancing blood flow, which increases glucose delivery to the prior ischemic-reperfused muscle.

### IIR and 70%/95%EX activate AMPK to a similar extent.

**Figure 2:**
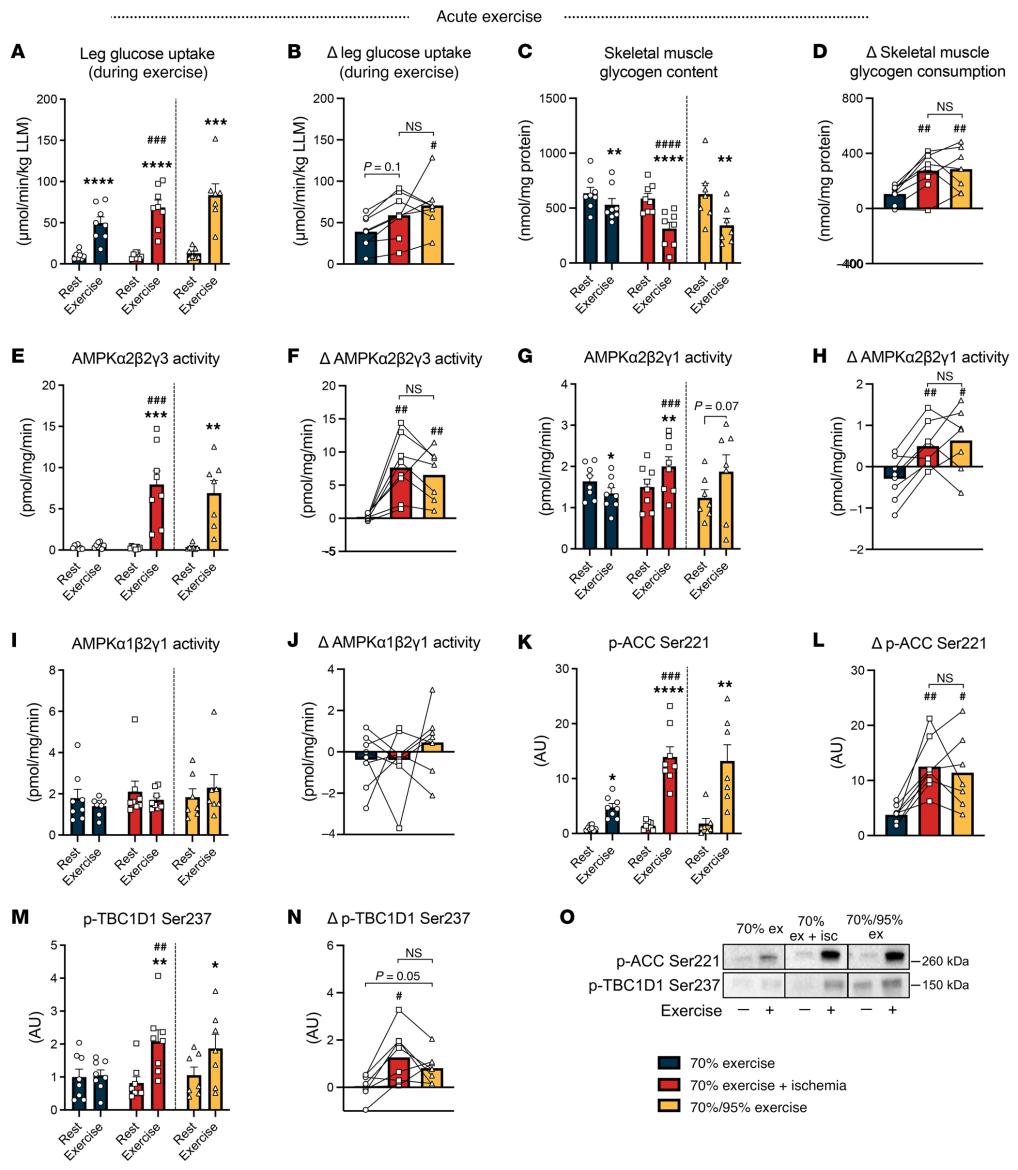
IIR-70%EX and 70%/95%EX enhance AMPK activation and glycogen consumption compared with 70%EX. (**A** and **B**) Leg glucose uptake during each exercise trial and the change in (Δ) glucose uptake between the rested and exercised leg. Pre- and immediately postexercise muscle glycogen content (**C**), AMPK activities(**E**, **G**, and **I**), ACC Ser221 phosphorylation(**K**), and TBC1D1 Ser237 phosphorylation (**M**). (**D**, **F**, **H**, **J**, **L**, and **N**) Change (Δ) in muscle glycogen content, AMPK activities, ACC Ser221, and TBC1D1 Ser237 phosphorylation from rest to exercise. (**O**) Representative immunoblot band images. *n* = 8 in the 70%EX and 70%EX + ischemia groups; *n* = 7 in the 70%/95%EX group. Data are means ± SEM. (**A**, **C**, **E**, **G**, **I**, **K**, and **M**) Comparisons between 70%EX and 70%EX + ischemia were performed using 2-way repeated-measures ANOVA; when a significant interaction was detected, post hoc Bonferroni-Šidák tests were applied. For 70%/95%EX, the exercise effect was evaluated using a paired (2-tailed) Student’s *t* test. (**B**, **D**, **F**, **H**, **J**, **L,** and **N**) One-way repeated-measures ANOVA was used, followed by Tukey’s post hoc test when significance was found. *P< 0.05, **P< 0.01, ***P < 0.001, and ****P< 0.0001 vs. rest within each trial; ^#^*P* < 0.05, ^##^*P* < 0.01, ^###^*P* < 0.001, and ^####^*P* < 0.0001 vs. 70%EX within either rested or exercised leg. LLM, lean leg mass.

Considering our prior findings demonstrating a role of AMPK for postexercise insulin sensitization (10), we investigated glucose uptake, glycogen consumption, and AMPK activation in skeletal muscle immediately after exercise completion. After 70%EX, the exercised muscle exhibited higher levels of glucose uptake, lactate release, and glycogen consumption compared with resting muscle. Both the IIR-70%EX and 70%/95%EX conditions elicited comparable increases in glucose uptake, lactate release, and glycogen consumption, exceeding the changes observed with 70%EX alone (Figure 2, A–D, Supplemental Figure 2, A and D, and Supplemental Table 1). Muscle phosphocreatine content and AMPKα2β2γ3 complex activity were significantly decreased and increased, respectively, by IIR-70%EX and 70%/95%EX but not by 70%EX (Figure 2, E and F, and Supplemental Table 1). A similar trend was observed for AMPKα2β2γ1 complex activity (Figure 2, G and H). Activity of the AMPKα1-containing complex did not change following any of the exercise trials (Figure 2, I and J). As a readout of endogenous AMPK activity, we measured phosphorylation of ACC Ser^221^ and TBC1D1 Ser^237^. At the 70%EX level, ACC Ser^221^ phosphorylation was significantly increased, which was potentiated by IIR-70%EX and 70%/95%EX (Figure 2, K and L). Although there was no significant increase in TBC1D1 Ser^237^ phosphorylation by 70%EX, the phosphorylation was significantly increased by IIR-70%EX and 70%/95%EX (Figure 2, M–O). Total protein content of AMPKα2, ACC, and TBC1D1 was not regulated by exercise (Supplemental Figure 7). Unexpectedly, the muscle AMP/ATP and ADP/ATP ratios did not change in response to exercise (Supplemental Figure 1, C and E). However, we observed that the inosine monophosphate levels, a more sensitive marker of ATP utilization, increased following IIR-70%EX and 70%/95%EX but not 70%EX (Supplemental Figure 1F). The respiratory exchange ratio as well as plasma FA, TG, glycerol, adrenaline, and noradrenaline levels did not differ among the 3 exercise trials (Table 2 and Supplemental Figure 2, E–J). Collectively, these data suggest that IIR-70%EX and 70%/95%EX induced a similar degree of AMPK activation and metabolic stress in skeletal muscle, which was augmented compared with the effect of 70%EX.

Leg O_2_ uptake was calculated because IIR during exercise could restrict oxygen supply that may compromise ATP production and, thus, enhance AMPK activation in skeletal muscle. However, the increase in leg O_2_ uptake during exercise was not different between 70%EX and IIR-70%EX, although it was significantly elevated after 70%/95%EX (Supplemental Figure 2, L–N).

To further explore the relationship between AMPK activation and acute exercise-induced glucose uptake prior to the insulin clamp, we examined the correlation between these parameters. As previously reported (18), AMPK activation following 70%/95%EX was significantly correlated with leg glucose uptake and the A-V glucose difference during the postexercise period but not during the exercise bout itself. Interestingly, this correlation was not observed in the IIR-70%EX condition (Supplemental Figure 3), suggesting that although both interventions similarly activate AMPK, the mechanisms linking AMPK activation to glucose uptake may differ between 70%/95%EX and IIR-moderate exercise.

### IIR and high-intensity exercise enhance phospho-TBC1D4, but only IIR elevates phospho-eNOS.

**Figure 3:**
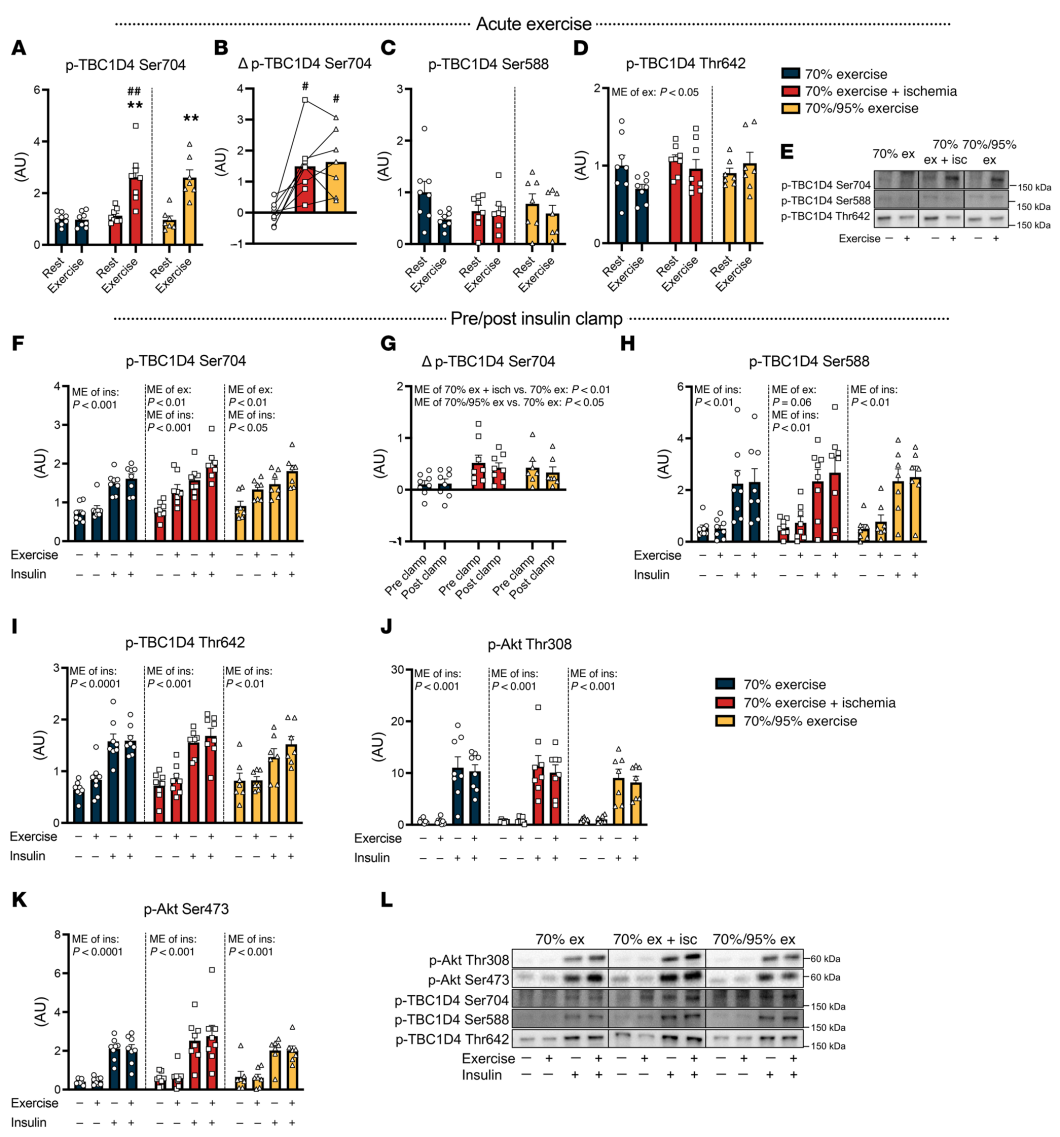
Enhanced phosphorylation of TBC1D4 after exercise and insulin stimulation. (**A**–**D**) Phosphorylation of TBC1D4 Ser704 (**A**), Ser588 (**C**), and Thr642 (**D**) before and immediately after exercise, along with (Δ) changes in TBC1D4 Ser704 phosphorylation (**B**) between the rested and exercised leg. (**E**) Representative immunoblot band images. (**F**–**K**) Phosphorylation of TBC1D4 Ser704 (**F**), Ser588 (**H**), and Thr642 (**I**), as well as Akt Thr308 (**J**) and Ser473 (**K**) before and after insulin stimulation. (**G**) (Δ) Changes in TBC1D4 Ser704 phosphorylation between the rested and exercised leg before and after the insulin clamp. (**L**) Representative immunoblot band images. *n* = 8 in the 70%EX and 70%EX + ischemia groups; *n* = 7 in the 70%/95%EX group. Data are means ± SEM. (**A**, **C**, and **D**) Comparisons between 70%EX and 70%EX + ischemia were performed using 2-way repeated-measures ANOVA; when a significant interaction was detected, post hoc Bonferroni-Šidák tests were applied. For 70%/95%EX, the exercise effect was evaluated using a paired (2-tailed) Student’s *t* test. (**B**) A 1-way repeated-measures ANOVA was used, followed by Tukey’s post hoc test when significance was found. (**F** and **H**–**K**) Analysis was conducted with 2-way repeated-measures ANOVA for each intervention (i.e., 70%EX, 70%EX + ischemia, and 70%/95%EX). (**G**) Two-way repeated-measures ANOVA was used, followed by Tukey’s post hoc test when appropriate. **P < 0.01 vs. rest within each trial; ^#^*P* < 0.05 and ^##^*P* < 0.01 vs. 70%EX within either rested or exercised leg. Δ, change in; Ins, insulin; ME, main effect.

Insulin-stimulated muscle glucose uptake is facilitated by Akt-dependent phosphorylation of the Rab-GTPase–activating protein TBC1D4 (also known as AS160), which promotes GLUT4 translocation to the plasma membrane (19). We have reported that phosphorylation of TBC1D4 Ser^704^ is regulated by both exercise (AMPK) and insulin and appears to be responsible for the insulin-sensitizing effect of exercise (8–10, 20). Therefore, we investigated the phosphorylation signature of TBC1D4 in response to exercise and the subsequent insulin stimulus. Phosphorylation of TBC1D4 Ser^704^ increased to a similar extent immediately after IIR-70%EX and 70%/95%EX but was unchanged following 70%EX (Figure 3, A and B). Phosphorylation of TBC1D4 Ser^588^ and Thr^642^ was unaffected by all 3 exercise modalities (Figure 3, C–E). In all trials, insulin stimulation increased phosphorylation of TBC1D4 Ser^704^ in rested and prior exercised muscle (Figure 3F). In addition, we observed an additive effect of insulin and prior exercise on TBC1D4 Ser^704^ phosphorylation in the IIR-70%EX and 70%/95%EX trials (Figure 3F). The effect of exercise (Δ) on TBC1D4 Ser^704^ phosphorylation before and after insulin stimulation was significantly higher for the IIR-70%EX and 70%/95%EX trials compared with the 70%EX trial, but no difference between the IIR-70%EX and 70%/95%EX trial was observed (Figure 3G). Phosphorylation of TBC1D4 at Ser^588^ and Thr^642^ was also increased by insulin in both rested and prior exercised muscle but was unaffected by prior exercise (Figure 3, H and I). Insulin stimulation led to a significant increase in the phosphorylation of Akt Thr^308^ and Ser^473^ with no significant impact of prior exercise or any difference among the 3 trials (Figure 3, J–L). Total protein content of Akt and TBC1D4 was not regulated by exercise and insulin (Supplemental Figure 7). Together, these results suggest that neither proximal insulin signaling nor the phosphorylation pattern on TBC1D4 can account for the potentiating effect of IIR on exercise-induced muscle insulin-sensitization.

**Figure 4:**
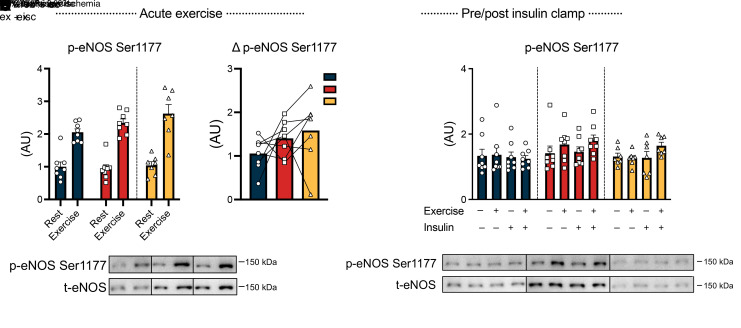
eNOS phosphorylation responses to exercise and insulin stimulation. (**A**–**C**) Phosphorylation of eNOS Ser1177 before and immediately after exercise as well as before and after the insulin clamp, along with (Δ) changes in Ser1177 phosphorylation (**B**) between the rested and exercised leg. *n* = 8 in the 70%EX and 70%EX + ischemia groups; and *n* = 7 in the 70%/95%EX group. Data are means ± SEM. (**A**) Comparison between 70%EX and 70%EX + ischemia was performed using 2-way repeated-measures ANOVA. For 70%/95%EX, the exercise effect was evaluated using a paired (2-tailed) Student’s *t* test. (**B**) A 1-way repeated-measures ANOVA was used. (**C**) Analysis was conducted using 2-way repeated-measures ANOVA for each intervention (i.e., 70%EX, 70%EX + ischemia, and 70%/95%EX). **P < 0.01 vs. rest within each trial. Δ, change in; ME, main effect. Ex, exercise; t-eNOS, total eNOS.

We examined endothelial nitric oxide synthase (eNOS) as another potential mechanism, given its role in regulating vasodilation via nitric oxide (NO) production in endothelial cells (21). Phosphorylation of eNOS plays a crucial role in regulating its activity, and aberrations in eNOS phosphorylation contribute to endothelial dysfunction and impaired vasodilation (21, 22). We observed that phosphorylation of eNOS Ser^1177^ increased immediately after exercise with no discernible difference among the 3 exercise modalities (Figure 4, A and B). Although insulin per se did not alter the phosphorylation of eNOS Ser^1177^, the exercise-induced increase was uniquely sustained throughout the insulin clamp in the IIR-70%EX trial (Figure 4C). This sustained eNOS phosphorylation may contribute to a prolonged enhancement of blood flow during the insulin clamp that potentially explains part of the potentiating effect of IIR exercise on muscle insulin sensitivity.

To evaluate the potential influence of circulating factors on muscle insulin sensitivity, we performed correlation analyses between the exercise-induced enhancement of insulin-stimulated glucose uptake and plasma levels of FA, TG, glycerol, adrenaline, and noradrenaline. These analyses were conducted for each exercise trial. However, none of these plasma metabolites or hormones showed a significant correlation with the degree of insulin-stimulated glucose uptake enhancement (Supplemental Figure 4). These findings suggest that the observed potentiation of insulin action by IIR exercise is unlikely to be driven by changes in systemic factors, reinforcing the notion that local adaptations in skeletal muscle and vasculature such as sustained eNOS phosphorylation and enhanced blood flow, play a more prominent role.

### Insulin-stimulated glycogen synthase activation and pyruvate dehydrogenase phosphorylation cannot explain the effect of IIR exercise.

**Figure 5:**
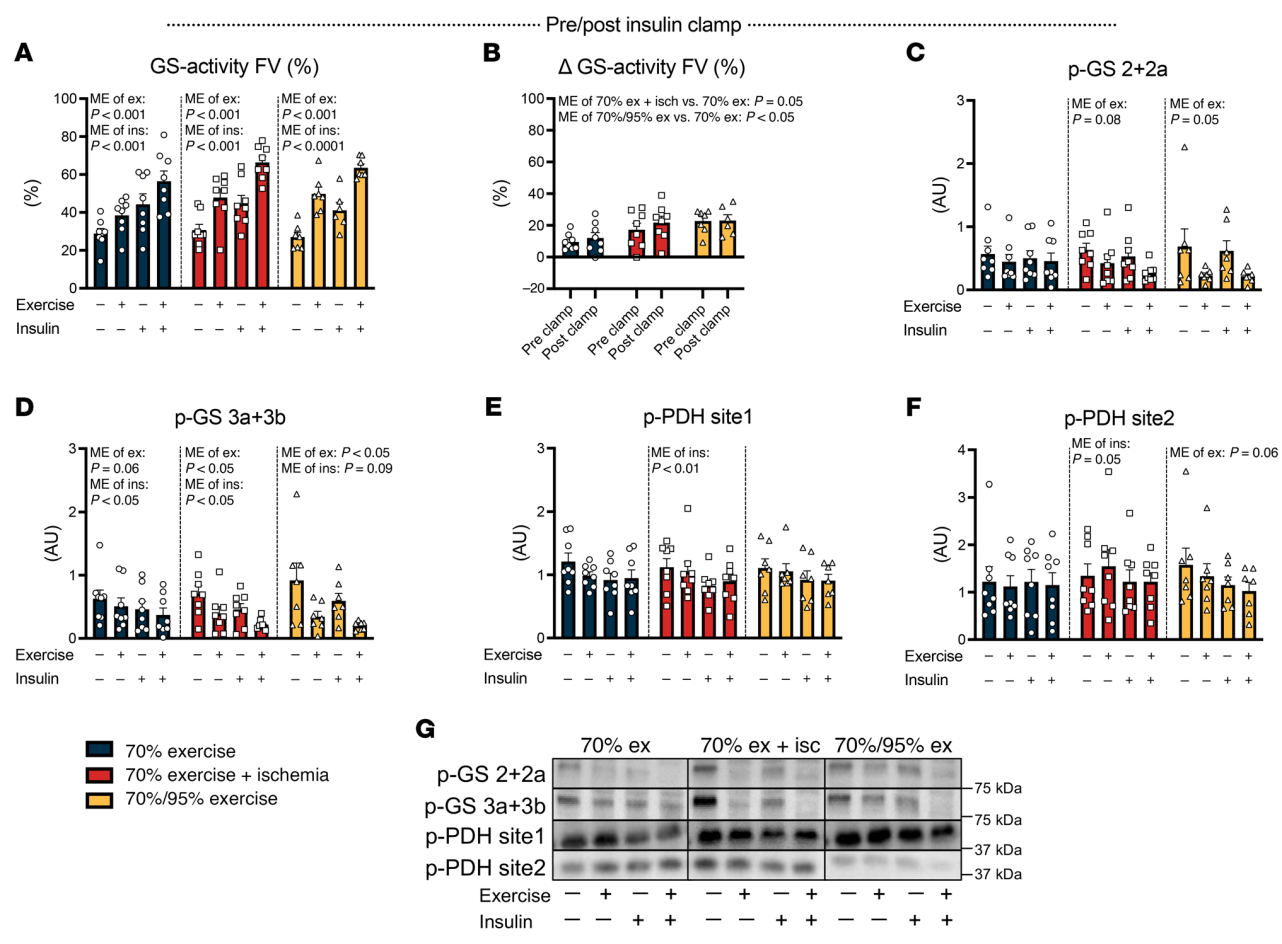
Insulin-stimulated GS activation and PDH phosphorylation do not regulate the insulin-sensitizing effect of exercise. (**A and C–F**) GS activity (percentage of fractional velocity) (**A**), GS site 2+2a (**C**), and 3a+3b (**D**) phosphorylation, as well as PDH site 1 (**E**) and PDH site 2 (**F**) phosphorylation in the prior rested and exercised leg before and after the insulin clamp. (**B**) (Δ) Changes in GS activity between the rested and exercised leg before and after the insulin clamp. (**G**) Representative immunoblot band images. *n* = 8 in the 70%EX and 70%EX + ischemia groups; *n* = 7 in the 70%/95%EX group. Data are means ± SEM. (**A** and **C**–**F**) Analysis was conducted using 2-way repeated-measures ANOVA for each intervention (i.e., 70%EX, 70%EX + ischemia, and 70%/95%EX). (**B**) Two-way repeated-measures ANOVA was used, followed by Tukey’s post hoc test when appropriate. Ex, exercise; Ins, insulin; ME, main effect.

The majority of glucose taken up during insulin stimulation is directed toward the glycogen pool, with glycogen synthase (GS) serving as the rate-limiting enzyme for incorporating glucose into glycogen. Accordingly, alterations in GS activity may influence the capacity of the muscle to take up glucose. GS activity increased immediately after exercise in all trials, but the increase was greater in the IIR-70%EX and 70%/95%EX trials (Supplemental Figure 5, A–C). This likely relates to the greater utilization of muscle glycogen (Figure 2D). These differences were maintained 3 hours into recovery immediately before insulin stimulation (Figure 5, A and B, and Supplemental Figure 5, E–G). Upon insulin stimulation, GS activity increased in muscle of both the rested leg and the prior exercised leg, but the insulin effect (Δ) was similar between legs in all 3 trials. Thus, GS activity levels were elevated in the prior exercised muscle of the IIR-70%EX and 70%/95%EX trials, but no differences between these 2 trials were observed. Similar to the activity levels, phosphorylation of GS site 2+2a and 3a+3b was regulated by prior exercise and insulin but was not differentially regulated among the 3 exercise trials (Figure 5, C and D). Collectively, this indicates that the regulation of GS activity by prior exercise and insulin is not responsible for the potentiating effect of IIR exercise on muscle insulin sensitivity.

Pyruvate dehydrogenase (PDH) is a rate-limiting enzyme, governing the entry of glucose into the tricarboxylic acid cycle for subsequent oxidation. Its activation is closely associated with glucose oxidation in human skeletal muscle during euglycemic insulin infusions (23). In our study, we observed an overall tendency for insulin to reduce phosphorylation of PDH site 1, whereas prior exercise tended to reduce phosphorylation of PDH site 2 only in the 70%/95%EX trial (Figure 5, E–G). Although we used phosphorylation as a proxy for PDH activity, this is not a direct enzymatic measure. However, PDH primarily regulates substrate choice rather than insulin sensitivity or glucose uptake per se (24). Thus, it is unlikely that PDH plays a central role in the IIR-induced improvement in insulin-stimulated glucose uptake.

Total muscle content of proteins associated with glucose disposal and oxidation, including GLUT4, hexokinase I, hexokinase II (HKII), PDH, and GS, were unaffected by insulin (Supplemental Figure 6). Although minor yet statistically significant changes in HKII and GS protein levels were observed following IIR-70%EX and 70%/95%EX, respectively (Supplemental Figure 6, D and E), these changes were not paralleled by corresponding changes in HK and GS enzymatic activities (Figure 5 and Supplemental Figure 5). Therefore, these modest alterations in protein expression are unlikely to contribute markedly to the potentiating effect of IIR exercise on muscle insulin sensitivity.

### Muscle metabolomic profiling reveals IIR-induced suppression of medium-chain acylcarnitines.

**Figure 6:**
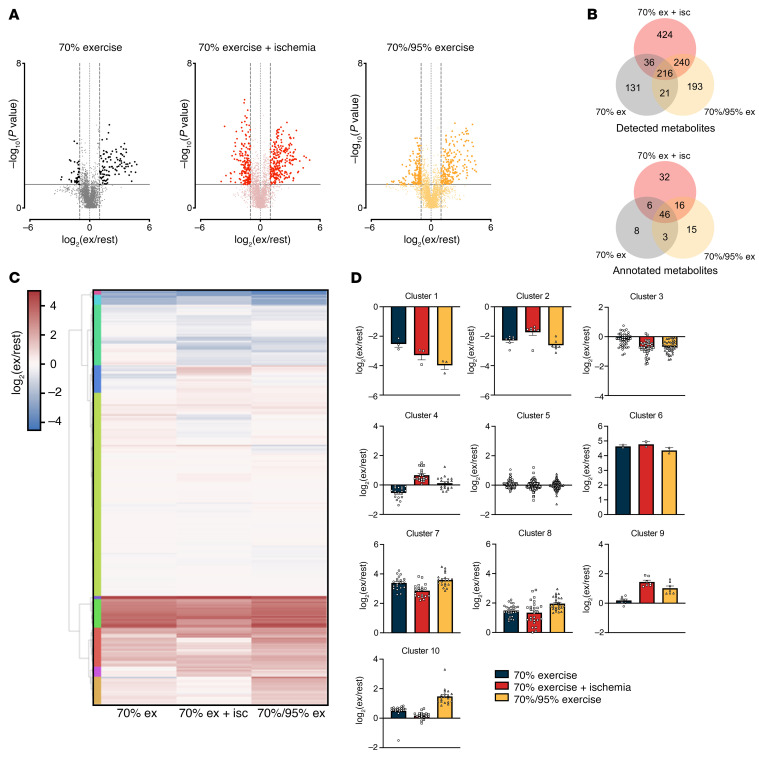
Untargeted metabolomics unveil distinct alterations in intramuscular metabolites across the 3 exercise modalities. Muscle biopsy specimens obtained before and immediately after each exercise trial were used for untargeted metabolomics analysis. (**A**) Summary of results showing significantly (*P* < 0.05, 2-tailed Student’s *t* test) up- and downregulated detected metabolites for each exercise modality (darker color in each panel) in contrast to nonregulated and detected metabolites (lighter color). (**B**) Venn diagrams showing significantly altered metabolites in common or specific for the different exercise trials. (**C**) Heat map showing the change from rest to exercise of all annotated metabolites for each exercise trial. (**D**) Average value of each cluster presented as a bar graph. *n* = 8 in the 70%EX and 70%EX + ischemia groups; *n* = 7 in the 70%/95%EX group. Data are means ± SEM. Ex, exercise.

**Figure 7:**
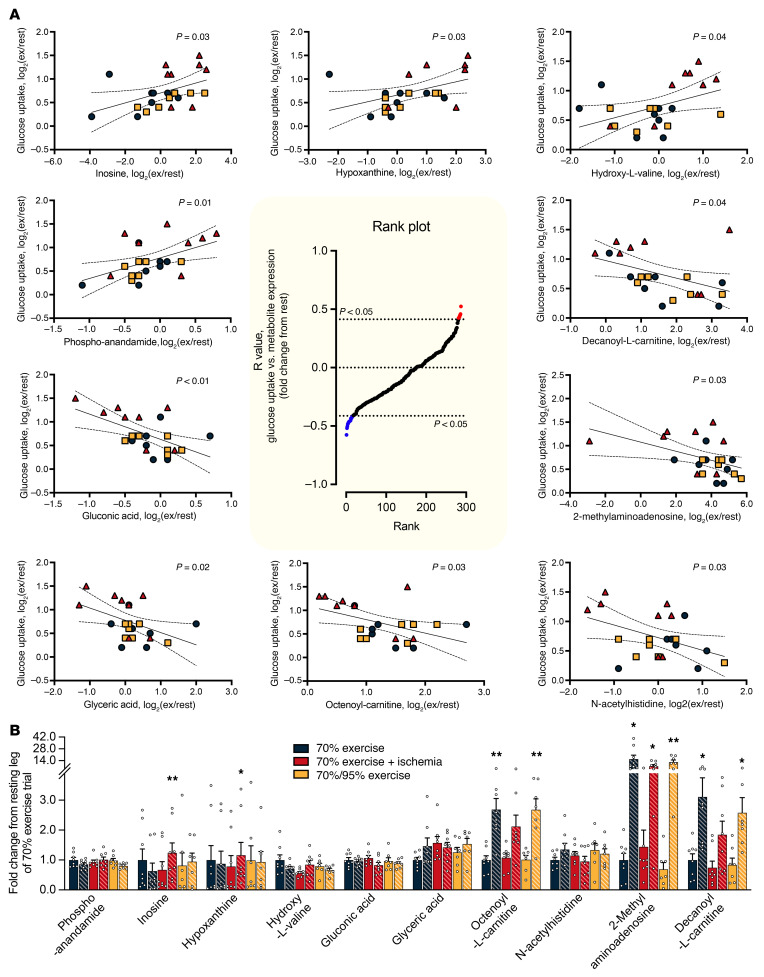
IIR suppresses the exercise-induced increase in decanoyl-l-carnitine, which correlates to muscle insulin sensitivity. (**A**) The center graph displays rank plots for the calculated *r* values between the exercise-induced change in metabolites and the insulin-stimulated change in leg glucose uptake. Surrounding graphs are scatter plots for selected metabolites that exhibited significant positive and negative correlations with the insulin-sensitizing effect of exercise. (**B**) Average fold-change values of the chosen metabolites, with blank bars representing the rested leg and bars with slanted lines representing the exercised leg. *n* = 8 in the 70%EX and 70%EX + ischemia groups; *n* = in the 7 70%/95%EX group. *n* = 12 in the rat study. A 2-way repeated-measures ANOVA was conducted for each metabolite, followed by Bonferroni-Šidák post hoc test when appropriate. Data are means ± SEM. Correlation analyses were performed using Pearson’s product–moment correlation coefficient. *P < 0.05 and **P < 0.01 vs. rest within each trial.

To elucidate potential regulatory factors underlying the enhanced insulin sensitization induced by IIR-70%EX, muscle samples obtained before and immediately after exercise in each trial were subjected to untargeted metabolomic analysis. This comprehensive analysis identified 3,745 metabolites, of which 404, 916, and 670 were significantly up- and downregulated following 70%EX, IIR-70%EX, and 70%/95%EX, respectively (Figure 6A and Supplemental Table 2A). The Venn-diagram in Figure 6B illustrates that 32 annotated metabolites were significantly regulated solely by IIR-70%EX, and 46 annotated metabolites displayed similar responses to all 3 exercise trials. All annotated metabolites were subsequently clustered in 10 groups according to their change from rest to exercise (Figure 6, C and D, and Supplemental Table 2B). The identification of these clusters led us to anticipate that metabolites exclusively affected by IIR-70%EX, or those unaffected by it, might serve as potential regulators of IIR-induced insulin sensitization. Correlation analyses between the exercise-induced changes in annotated metabolite levels and insulin-stimulated leg glucose uptake across the 3 exercise trials revealed significant positive (*n* = 7 metabolites) and negative (*n* = 17 metabolites) correlations (Figure 7A and Supplemental Table 2C). Within the group of significantly correlated metabolites, changes in inosine, hypoxanthine, 2-methyl-aminoadenosine, octenoyl-carnitine, and decanoyl-l-carnitine appeared to statistically explain the potentiating effect of IIR-70%EX on muscle insulin sensitivity (Figure 7B). Notably, the exercise-induced increases in octenoyl-carnitine and decanoyl-l-carnitine levels, both medium-chain acylcarnitines, were suppressed by IIR and emerged as candidate negative regulators of muscle insulin sensitivity.

### Manipulation of muscle acylcarnitines levels affect insulin sensitivity.

**Figure 8:**
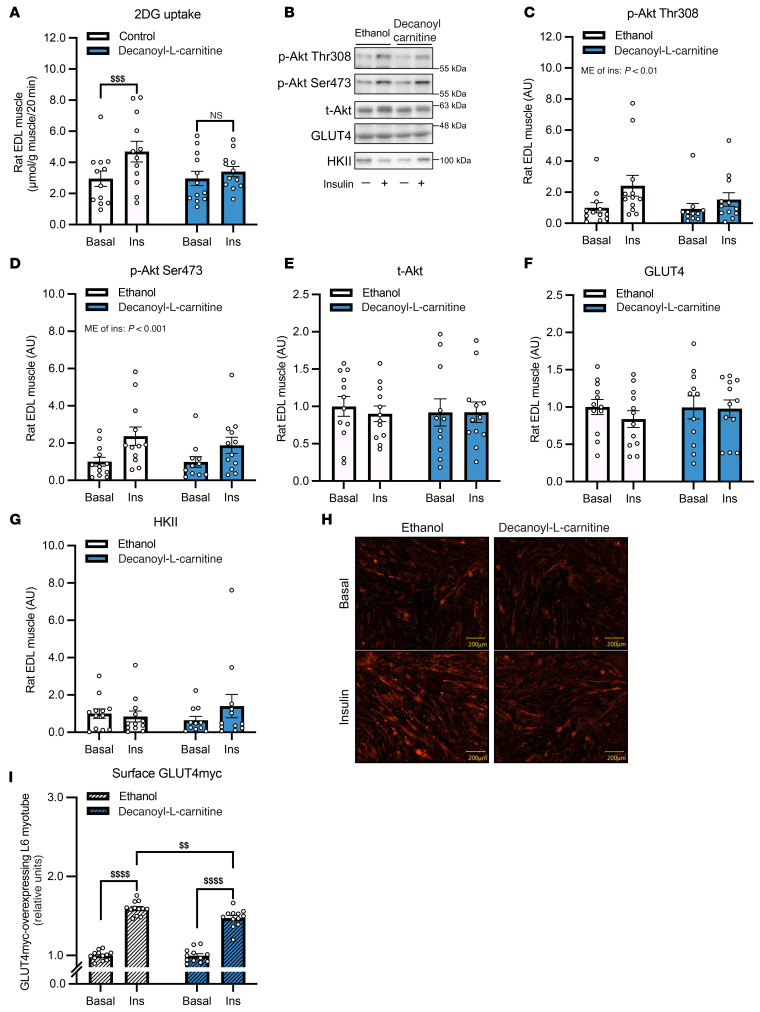
Decanoyl-l-carnitine impairs insulin-stimulated glucose uptake in rat skeletal muscle and plasma membrane GLUT4 translocation in L6 myotubes. (**A**) Uptake of 2-deoxyglucose (2DG) in isolated rat EDL muscle treated with 0.15 mM decanoyl-l-carnitine or vehicle (ethanol) under basal and insulin-stimulated conditions. (**B**) Representative immunoblots for Akt phosphorylation (Ser473 and Thr308), total Akt (t-Akt), GLUT4, and HKII from EDL muscle treated as in (**A**). Quantification of p-Akt Ser473 (**C**), p-Akt Thr308 (**D**), total Akt (**E**), GLUT4 (**F**), and HKII (**G**). (**H**) Representative immunofluorescence images showing surface GLUT4 in GLUT4-myc overexpressing L6 myotubes treated with 0.4 mM decanoyl-l-carnitine or ethanol under basal or insulin-stimulated conditions. Scale bar = 200 μm. (**I**) Quantification of surface GLUT4-myc levels in L6 myotubes shown in (**H**). Experiments using GLUT4-myc overexpressing L6 myotubes were repeated twice, confirming consistent results. *n* = 12 for EDL muscle 2DG uptake and immunoblots and for L6 cell assays. Data are means ± SEM. (**A** and **C**–**G**) Two-way ANOVA was performed, using a repeated-measures design. (**I**) A nonrepeated measures design was used, followed by Bonferroni-Šidák post hoc test when appropriate. ^$$^*P* < 0.01, ^$$$^*P* < 0.001, ^$$$$^*P* < 0.0001.

To further investigate the role of medium-chain acylcarnitines in muscle insulin sensitization, we conducted ex vivo experiments in which rodent skeletal muscle was incubated with decanoyl-l-carnitine. This treatment impaired insulin-stimulated glucose uptake in glycolytic extensor digitorum longus (EDL) muscle (Figure 8A) without affecting phosphorylation of Akt or total expression levels of Akt, GLUT4, or HKII (Figure 8, B–G). In line with these results, insulin-stimulated GLUT4 translocation to the plasma membrane was significantly blunted in GLUT4-myc–overexpressing L6 myotubes following exposure to decanoyl-l-carnitine (Figure 8, H and I). These findings suggest suppression of exercise-induced accumulation of decanoyl-l-carnitine by IIR may enhance insulin-stimulated glucose uptake via a mechanism involving enhanced GLUT4 translocation independently of canonical insulin signaling.

**Figure 9:**
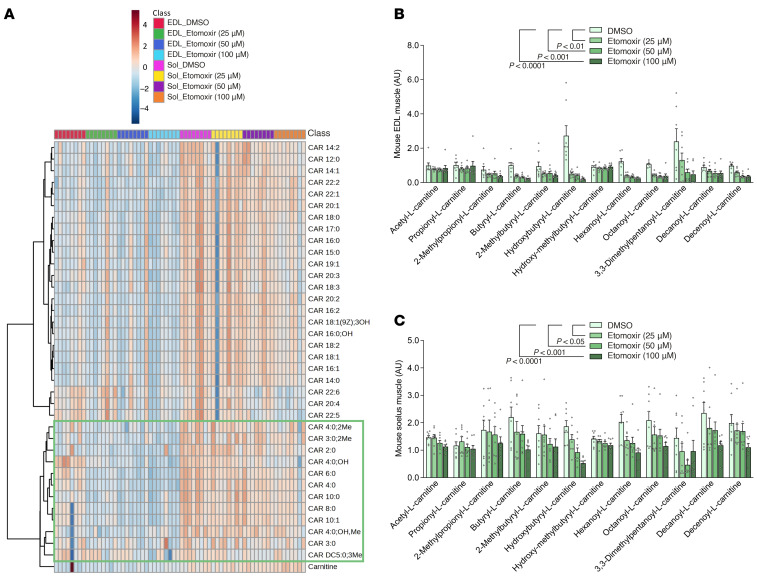
Etomoxir reduces muscle acylcarnitines in mouse skeletal muscle. (**A**) Heat map of carnitine and acylcarnitine species quantified by mass spectrometry in EDL and soleus muscles incubated with DMSO (vehicle) or etomoxir (25–100 μM). Blue and red colors indicate relative abundance (*z*-score normalized). Green box highlights a cluster that includes most short- and medium-acylcarnitines reduced by etomoxir. (**B** and **C**) Quantification of selected acylcarnitine species in green box at (**A**) in EDL (**B**) and soleus (**C**) muscles. The combined levels of 12 acylcarnitines were analyzed as a group to assess the effect of etomoxir using 1-way ANOVA. When significant differences were found, Dunnett’s post hoc test was applied to compare the DMSO group with the etomoxir-treated group. *n* = 4–10 per group. Data are presented as means ± SEM.

**Figure 10:**
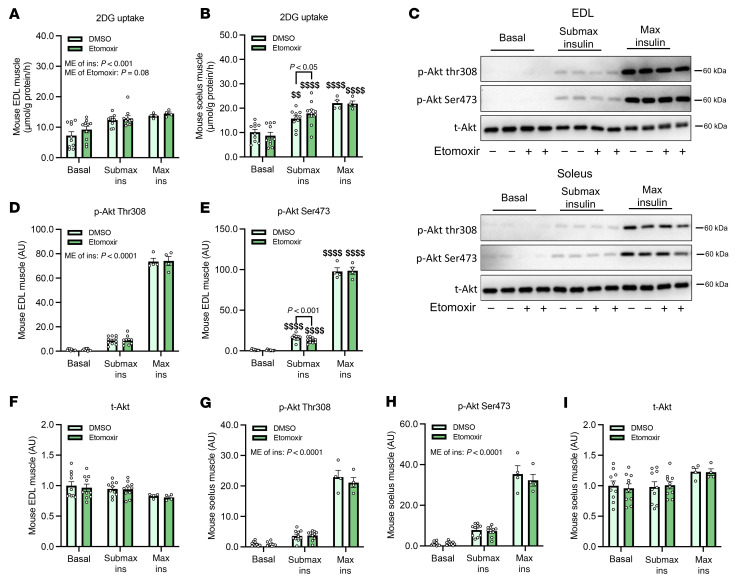
Etomoxir enhances insulin-stimulated glucose uptake in mouse skeletal muscle. (**A** and **B**) Uptake of 2-deoxyglucose (2DG) in mouse EDL (**A**) and soleus (**B**) muscles in response to submaximal (Submax) or maximal (Max) insulin (Ins) stimulation, with or without etomoxir. (**C**) Representative immunoblots showing phosphorylation of Akt (Thr308, Ser473) and total Akt in EDL and soleus muscle samples treated as in (**A** and **B**). (**D**–**F**) Quantification of p-Akt Thr308 (**D**), p-Akt Ser473 (**E**), and total Akt (**F**) in EDL muscle. (**G**–**I**) Quantification of p-Akt Thr308 (**G**), p-Akt Ser473 (**H**), and total Akt (**I**) in soleus muscle. *n* = 4-10 per group. Data are presented as means ± SEM. (**A,** **B**, and **D**–**I**) Two-way repeated-measures ANOVA was used, followed by Tukey’s post hoc test when appropriate. ^$$^*P* < 0.01, ^$$$$^*P* < 0.0001 vs. corresponding basal. ME, main effect.

In a complementary set of experiments, pharmacological inhibition of acylcarnitine synthesis using etomoxir, a CPT1 inhibitor, resulted in dose-dependent reductions of short-, medium-, and, to a lesser extent, long-chain acylcarnitine levels in isolated mouse skeletal muscle (Figure 9, A–C). This intervention led to a significant increase in submaximal insulin-stimulated glucose uptake in soleus muscle and showed a positive trend in EDL muscle without enhancing Akt phosphorylation (Figure 10, A–I). Together, these data support a model in which suppression of short- and medium-chain acylcarnitine levels contributes to enhanced muscle insulin sensitivity and further implicate acylcarnitine modulation as a potential mediator of the insulin-sensitizing effects observed after IIR-70%EX.

## Discussion

A single bout of exercise enhances insulin-stimulated glucose uptake in skeletal muscle, and we have previously shown in animal models that this phenomenon is caused by enhanced AMPK activation (9, 10). In the present study, we show that IIR potentiated the effect of moderate-intensity exercise on AMPK activity and insulin sensitivity in human skeletal muscle. In contrast, although the activation of AMPK induced by increased exercise intensity is similar to that elicited by IIR, it did not enhance the exercise-induced improvements in muscle insulin sensitivity. These results suggest that IIR potentiates muscle insulin sensitivity independently of AMPK activation and that maximizing AMPK activation in skeletal muscle is not necessary to take full advantage of the insulin-sensitizing effect of exercise. Intriguingly, untargeted metabolomics, supported by subsequent rodent muscle experiments, revealed that the suppression of the exercise-induced increase in medium-chain acylcarnitine levels is involved in the potentiation of muscle insulin sensitivity by IIR. Additionally, exercise combined with IIR elevates insulin-stimulated blood flow that is likely linked to increased NO-mediated vasodilation following eNOS phosphorylation. Collectively, IIR potentiates postexercise muscle insulin sensitivity by reshaping the intramuscular metabolite profile and by enhancing glucose delivery. This extends beyond the proposed role of AMPK for muscle insulin sensitization after exercise.

The accumulation of decanoyl-l-carnitine in muscle tissue or plasma has been linked to insulin resistance (25–27). Additionally, insulin resistance has been shown to develop in primary cultured myotubes when exposed to a combination of long- and medium-chain acylcarnitines (28) and insulin-stimulated glucose uptake is compromised in 3T3-L1 adipocytes incubated with 0.1 mM decanoyl-l-carnitine (29). These findings are consistent with our current results showing that 0.15 mM decanoyl-l-carnitine impairs insulin-stimulated glucose uptake in rat EDL muscle. To further test the role of acylcarnitines, we reduced their levels using etomoxir, a CPT1 inhibitor. Etomoxir dose dependently decreased levels of short- and medium-chain acylcarnitines and enhanced submaximal insulin-stimulated glucose uptake in soleus muscle, with a similar trend seen in EDL muscle. These results support the idea that medium-chain acylcarnitines negatively regulate muscle insulin sensitivity and that their suppression improves glucose uptake.

Previous studies have demonstrated that high-intensity exercise elevates short-chain acylcarnitine levels in both human skeletal muscle and plasma while displaying no notable impact on long-chain acylcarnitines (30). This increase in short-chain acylcarnitine levels after exercise coincides with a drop in muscle carnitine levels (31), a trend also observed in our etomoxir experiment, where pharmacological inhibition of CPT1 led to a dose-dependent reduction in acylcarnitines along with an increase in carnitine levels (Figure 9A). Certain specific short-chain acylcarnitines exhibit a favorable association with fat oxidation during periods of rest and after exercise, suggesting their potential role in augmenting postexercise fat utilization (31). Additionally, whereas long-chain acylcarnitines like oleoyl-l-carnitine and stearoyl-l-carnitine remain unaffected by exercise, we identified an exercise-induced increase in short- and medium-chain acylcarnitine levels (e.g., butyryl-l-carnitine, octanoyl-l-carnitine, octenoyl-l-carnitine, decanoyl-l-carnitine, decatrienoy-l-carnitine), which was suppressed by IIR. As a result, the application of IIR appeared to inhibit the synthesis of acylcarnitines, notably decanoyl-l-carnitine, from carnitine during exercise, potentially amplifying insulin sensitivity and favoring utilization of glucose over FAs as the primary fuel source during the subsequent recovery period. This shift in substrate preference is consistent with the Randle cycle concept, according to which decreased FA oxidation promotes increased glucose utilization, highlighting metabolic flexibility as an adaptive mechanism that optimizes substrate use in response to exercise and IIR.

Recently, we reported that the interstitial glucose concentration in human skeletal muscle decreases during a hyperinsulinemic-euglycemic clamp and that prior exercise enhances this reduction, due to an increase in the muscle membrane permeability for glucose (32). This increased permeability is believed to be important for the insulin-sensitizing effect of exercise (11), and we have evidence to suggest this might be further enhanced if blood flow and, thus glucose delivery, is increased (32). In support of this, a rodent study demonstrated improved muscle glucose uptake following pharmacological enhancement of microvascular perfusion (33). Considering this, we speculate that the ischemia-induced reperfusion during exercise promotes endothelial shear stress, leading to elevated eNOS activity levels and, consequently, enhanced insulin-stimulated vasodilation (17). Indeed, this is supported by our findings of enhanced eNOS phosphorylation, accompanied by increased femoral artery blood flow and insulin-stimulated glucose uptake in the previously exercised leg of the IIR-70%EX trial. Furthermore, in an independent protocol involving intravenous infusion of l-NMMA (a NOS inhibitor) in humans (7), we observed reduced eNOS Ser^1177^ phosphorylation (data not shown) in parallel with a blunted postexercise enhancement of insulin-stimulated blood flow and glucose uptake in skeletal muscle (7), providing additional support for the contribution of eNOS-mediated vasodilation to this effect. This observation implies that an increase in glucose delivery to the insulin-sensitized muscle contributes markedly to the potentiation of insulin-stimulated glucose uptake.

Originally, we hypothesized that both IIR-70%EX and 70%/95%EX would lead to further improvements in muscle insulin sensitivity compared with 70%EX, because muscle insulin sensitivity is regulated by AMPK activation (10, 34, 35) as well as decreased glycogen levels (21, 36). As expected, exercise-induced AMPK activation, glycogen consumption, and leg lactate release were similar between IIR-70%EX and 70%/95%EX but increased compared with 70%EX. Intriguingly, the insulin-sensitizing effect was greater with IIR-70%EX compared with both 70%/95%EX and 70%EX. In a study of rodents, improvements in muscle insulin sensitivity was higher after low-intensity continuous swimming exercise compared with high-intensity short-term swimming exercise, whereas phosphorylation of AMPK Thr^172^ as well as muscle glycogen consumption were greater after high-intensity, short-term swimming exercise (37). Together, these observations indicate the magnitudes of AMPK activation and glycogen consumption achieved following exercise do not determine the magnitude of the subsequent improvement in insulin sensitivity. The latter is consistent with our recent meta-analyses of human data (38) and suggests that although glycogen consumption might still be necessary, it does not play a substantial role in regulating the magnitude of muscle insulin sensitivity after exercise in humans. This reinforces the notion that further improvements in muscle insulin sensitivity by IIR does not seem to be regulated by AMPK activation and glycogen consumption.

Previously, we showed that the enhancing effect of exercise on skeletal muscle insulin sensitivity is regulated by the AMPK-TBC1D4 signaling pathway (9, 10). Specifically, we propose that the AMPK downstream target TBC1D4 Ser^704^ is likely the pivotal phosphorylation site that regulates muscle insulin sensitivity after exercise (8). However, in the present study, 70%EX did not increase levels of phosphorylated TBC1D4 Ser^704^ (p-TBC1D4 Ser^704^) either immediately or 3 hours after exercise, despite improvements in muscle insulin sensitivity. One possible explanation for this discrepancy may reside in a technical limitation to detect subtle changes in p-TBC1D4 Ser^704^ at relatively low exercise intensities. Indeed, knee extension exercise at 70% peak workload for 60 minutes is the lightest workload used compared with our previous studies reporting effects of exercise on p-TBC1D4 Ser^704^ in human skeletal muscle (7, 20, 39–41). Another explanation may be that p-TBC1D4 Ser^704^ determines how long the insulin-sensitizing effect of exercise is maintained. Additionally, species differences may also contribute to the observed discrepancy. It was recently suggested that p-TBC1D4 Ser^704^ is not essential for the insulin-sensitizing effect of exercise in rats (42), whereas our previous evidence was based on mouse skeletal muscle (8). Nevertheless, we observed no differences in p-TBC1D4 Ser^704^ levels between the IIR-70%EX and 70%/95%EX trials. Given that insulin sensitivity was potentiated following IIR-70%EX compared with 70%/95%EX, these results indicate that p-TBC1D4 Ser^704^ is not important for mediating the effect of IIR on muscle insulin sensitivity.

In summary, our study demonstrates that IIR potentiates the insulin-sensitizing effect of exercise in skeletal muscle from healthy lean men independently of the AMPK-TBC1D4 signaling axis. Conversely, our metabolomics analysis, supported by experiments in rodent muscle, identified medium-chain acylcarnitines, particularly decanoyl-l-carnitine, as potential mediators of the IIR-induced enhancement of postexercise insulin sensitivity. Additionally, IIR may augment glucose delivery by increasing blood flow, thereby enhancing the potential of the muscle to extract glucose from the circulation. Notably, further activation of AMPK by increasing exercise intensity does not augment the insulin-sensitizing effect of exercise on muscle glucose uptake. These findings hold substantial importance because they offer valuable insight on how to maximize the insulin-sensitizing effect of exercise as well as increase our understanding of how exercise induces signaling in muscle to enhance insulin sensitivity in humans. Moreover, we speculate that combining IIR with low- to moderate-intensity exercise may represent a promising intervention to explore in future studies as a potential alternative for individuals with impaired physical capacity. Given the observed enhancement in insulin sensitivity with IIR under relatively moderate exercise conditions, this approach warrants further investigation to determine its applicability and efficacy in populations with insulin resistance or limited exercise tolerance.

## Methods

### Sex as a biological variable.

Our study focused on male human participants and male rats because they exhibited less variability in phenotype.

### Human experimental protocol.

Eight young and healthy male volunteers (Supplemental Table 3) were included in the study. Prior to the first experimental day, body composition was determined by dual-energy X-ray absorptiometry (DPX-IQ Lunar; Lunar Corp.). The maximal oxygen uptake (VO_2_) peak was determined on a bike ergometer (Monark AB) by an incremental test to exhaustion using breath-by-breath measurements of VO_2_ (Master-Screen CPX; Intramedic A/S). The participants were subsequently familiarized with the 1-legged knee extensor ergometer and, on a separate day, a minimum 1 week prior to the first experimental day, peak aerobic workload (PWL) of the knee extensors was determined in both legs by an incremental test (43). The participants were instructed to record food intake for 3 days and to abstain from alcohol, caffeine, and strenuous physical activity for 48 hours before the experimental day. Participants were investigated on 3 experimental days separated by at least 2 weeks. They were instructed to follow the same dietary regime before all experimental days.

On each experimental day, the participants arrived at the laboratory in the early morning following an overnight fast (~12 hours) after which they ingested a light breakfast (oatmeal, skimmed milk, sugar; 5% of daily energy intake). The participants then rested in the supine position for 3 hours during which catheters (Pediatric Jugular Catheterization set; Arrow International) were inserted into the femoral artery of 1 leg and femoral veins of both legs under local anesthesia (xylocaine 1%; AstraZeneca). Following rest, the participants were then randomized to perform 1 of 3 different knee-extensor exercise protocols for 60 minutes. The exercising leg was randomly chosen, but for each individual, the same leg was exercised in all 3 trials. The exercise protocols consisted of (a) 70% of PWL (70%EX) for 1 hour; (b) 70% of PWL with 25 seconds of added ischemia (>250 mmHg to upper thigh) every 2 minutes (IIR-70%EX); and (c) 70% of PWL evenly interspersed with six 5-minute intervals at 95% of PWL (70%/95%EX). Leg ischemia was imposed by blood flow occlusion induced by a pneumatic tourniquet (13 cm width) placed proximal on the leg thigh. The pressure was momentarily increased to greater than 250 mmHg and instantly deflated by an automated cuff device (Rapid Cuff Inflation System; DE Hokanson Inc.). Complete leg blood-flow occlusion was verified by confirming no popliteal-artery blood flow during ischemia of the exercising leg. A noninflated cuff was placed on the thigh of the nonexercising leg. Before exercise, as well as at 30 and 60 minutes after the initiation of exercise (during the reperfusion phase in the IIR-70%EX trial), blood samples were drawn from the artery and both venous catheters, and femoral artery blood flow was measured in both legs using the ultrasound Doppler technique (Phillips iU22; ViCare Medical A/S). This enabled the assessment of leg balance for various metabolites, using Fick′s principle. Specifically, leg glucose uptake (μmol/min/kg) was calculated as: leg glucose uptake = leg blood flow (mL/min/leg) × [glucose]A-V (μmol/mL).

Three hours after the exercise bout, a 120-minute euglycemic-hyperinsulinemic clamp was initiated with a bolus of insulin (9 mU/kg) (Actrapid; Novo Nordisk) followed by 120 minutes of constant insulin infusion (1.42 mU/min/kg). Euglycemia was maintained by infusion of a 20% glucose solution. Blood samples were drawn from all 3 catheters at 15, 30, 60, 90, and 120 minutes into the clamp. Prior to blood sampling, femoral arterial blood flow was measured (as described). Muscle biopsies of the vastus lateralis muscle were obtained in the rested leg before exercise and in the exercised leg immediately after exercise, as well as in both legs before and at the end of the insulin clamp (120 minutes) under local anesthesia (2% xylocaine) (Figure 1A) using the Bergström needle technique with suction. The biopsy material was forced out of the needle into a metal sieve by rinsing the inner tube of the needle with an ice-cold physiological and sterile saline solution. The material was then quickly placed on filter paper to absorb any fluid and subsequently frozen in liquid nitrogen. Muscle samples were stored at –80°C until further use.

### Animal study.

Male Wistar rats (CLEA Japan) aged 4 weeks were subjected to overnight fasting. Under inhalational anesthesia with isoflurane, the EDL muscle was dissected. The isolated muscles were then incubated as previously described (44). In brief, the EDL muscles were placed in 4 mL of oxygenated Krebs–Henseleit bicarbonate buffer supplemented with 39.85 mM mannitol and 0.1% bovine serum albumin, with or without 0.15 mM decanoyl-l-carnitine. The incubation was carried out for 30 minutes with continuous shaking at 30°C, and the flasks were continuously gassed with a mixture of 95% O_2_ and 5% CO_2_. Following the 30-minute incubation, the EDL muscles were incubated for an additional 20 minutes at 30°C in the same buffer, either with or without 50 μU/mL insulin. This was followed by a 20-minute incubation in the buffer supplemented with 8 mM 2-deoxyglucose. The mannitol concentration in all incubation buffers was adjusted to maintain an osmolarity of 40 mM. The concentration of 2-deoxyglucose-6-phosphate in the muscle was subsequently determined using a method previously described (45).

Female C57Bl/6 mice, which generally exhibit higher insulin responsiveness than males, were used to assess the potential insulin-sensitizing effect of etomoxir. Mice (Taconic Biosciences), aged 11–15 weeks, were anesthetized by a single intraperitoneal injection of pentobarbital and xylocain (100 mg/kg and 5 mg/kg, respectively), after which the soleus and EDL muscles were removed, suspended in heated (30°C), and continuously gassed (95% O_2_ and 5% CO_2_) incubation chambers (Multi Myograph System; Danish Myo Technology) containing Krebs-Ringer buffer supplemented with 0.1% bovine serum albumin, 8 mM mannitol, and 2 mM pyruvate (KRB) as previously described (46). After approximately 15 minutes of preincubation in KRB, muscles were incubated for 60 minutes in KRB containing etomoxir (50 μM) or vehicle (DMSO). Thirty minutes into the etomoxir stimulation protocol, muscles were stimulated with insulin (0, 100, and 10,000 μU/mL) for 30 minutes. The uptake of 2-deoxyglucose was measured during the last 10 minutes of the 30-minute stimulation period by adding 1 mM [3H]-2-deoxyglucose (0.056 MBq/mL), 7 mM [14C]-mannitol (0.0167 MBq/mL) (Hartmann Analytic), and 2 mmol/l-pyruvate to the incubation buffer. Muscles used for analyses of acylcarnitine levels were stimulated by etomoxir (25, 50, and 100 μM or vehicle), but not by insulin, for 60 minutes. After incubation, muscles were washed in ice-cold KRB, dried on filter paper, and frozen in liquid nitrogen until further analyses. Uptake of 2-deoxyglucose was determined as previously described (8–10).

### Cell culture study.

L6-GLUT4myc rat myoblasts (Kerafast Inc.; ESK202) were cultured in MEMα (Fujifilm Wako; 137-17215) supplemented with 10% FBS, 1% penicillin-streptomycin, and 2 μg/mL blasticidin. Upon reaching confluence, cells were induced to differentiate into myotubes by switching to MEMα containing 2% FBS and 1% antibiotics for 8 days.

On day 8 of differentiation, myotubes were stimulated with either vehicle (ethanol) or 0.4 mM decanoyl-l-carnitine for 1 hour. This was followed by a 20-minute stimulation with 10 nM insulin to assess submaximal insulin responsiveness. After stimulation, cells were washed with ice-cold PBS and fixed with 3% paraformaldehyde. After fixation, cells were incubated with an anti-myc primary antibody (Fujifilm Wako; 017-21871) and an appropriate fluorophore-conjugated secondary antibody (Fujifilm Wako; 115-167-003). GLUT4myc translocation was quantified by high-content imaging using the CQ1 system (Yokogawa Electric Corp.).

### Biogeochemical analysis.

Methods for muscle processing; plasma, muscle glycogen, and metabolites; AMPK, GS, and HK activity; muscle metabolomics; acylcarnitine analysis; and Western blot analysis are all presented in the online-only Supplemental Materials.

### Statistics.

One participant withdrew from the study after having completed the 70%EX and IIR-70%EX trials; thus, analyses are based on 8 participants in both the 70%EX and IIR-70%EX trials and 7 in the 70/95%EX trial. Data are presented as mean ± SEM. Statistical significance within and between groups was assessed using a 2-way ANOVA — with repeated measures where applicable — and paired (2-tailed) Student’s *t* tests. A 1-way ANOVA with or without repeated measures was used to assess statistical difference of the acute exercise effect (Δ) between the groups. For post hoc testing, the Bonferroni-Šidák or Tukey’s tests were used as appropriate, except for Figure 9, B and C, where Dunnett’s test was applied to specifically compare treatment groups with the control. Correlation analyses were performed by calculating the Pearson’s product-moment correlation coefficient. Statistical significance was defined as *P* < 0.05.

### Study approval.

This human study was approved by the Copenhagen Regional Ethics Committee (H-18006850) and complied with the guidelines of the Declaration of Helsinki. Written informed consent was obtained from all participants prior to entering the study. The animal studies were conducted with the approval of the Animal Care and Use Committee of Fukuoka University (approval no. 2107040 and 2215108) as well as by the Danish Animal Experiments Inspectorate (Copenhagen, Denmark) (license number: 2019-15-0201-01659).

### Data availability.

All data needed to evaluate the conclusions in this article are present in the article and/or the supplemental materials, including the Supporting Data Values file.

## Author contributions

K Kido, JFPW, and RK designed and managed the human trial and performed the human experiments along with JRH, JO, and KAS. K Kido, JFPW, and RK designed the animal work. K Kido directed and managed the animal work and performed the mouse experiments along with TZ and K Kawanaka. K Kido, JBB, NOE, TZ, JFH, NJF, YH, and RK performed the biochemical analyses. K Kido drafted the first version of the manuscript. All authors interpreted the results, contributed to the discussion, edited and revised the manuscript, and read and approved the final version of the manuscript. K Kido, JFPW, and RK are the guarantors of this work and, as such, have full access to all the data in the study and take responsibility for the integrity of the data and the accuracy of the data analysis.

## Funding support

- Danish Council for Independent Research (FSS) grant 8020-00288B to JFPW.
- Novo Nordisk Foundation grants NNF0070370 and NNF082659 to JFPW.
- Japan Society for the Promotion of Science, Grants-in-Aid for Scientific Research 18J01392, 19K20007, and 24K02839 to K Kido.
- European Foundation for the Study of Diabetes/ Japan Diabetes Society Fellowship Program to K Kido.

## Supplementary Material

Supplemental data

Unedited blot and gel images

Supplemental table 2

Supporting data values

## Figures and Tables

**Table 2:**
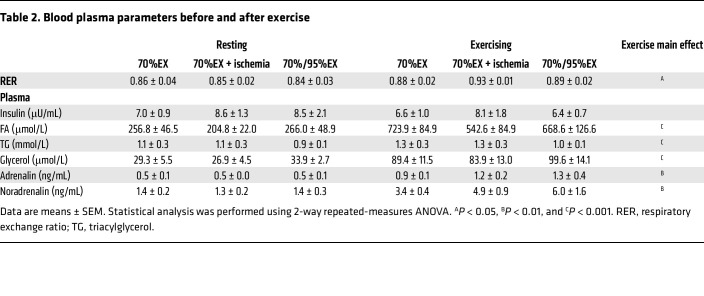
Blood plasma parameters before and after exercise

**Table 1:**
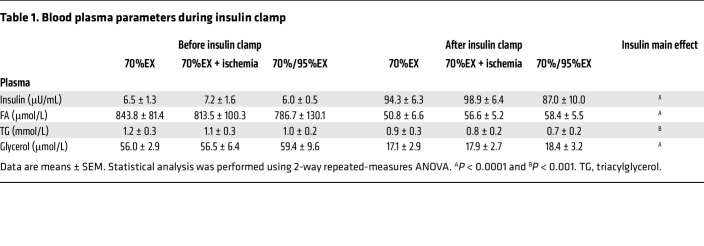
Blood plasma parameters during insulin clamp

## Version 1 09/02/2025

In-Press Preview

## Version 2 11/03/2025

Electronic publication
