# Supplementary material for: Intermittent ischemia/reperfusion as a potent insulin-sensitizing intervention via blood flow enhancement and muscle decanoyl-l-carnitine suppression
Source: J Clin Invest. 2025 Sep 2;135(21):e183567. doi: 10.1172/JCI183567 (PMC12578404; doi:10.1172/JCI183567)
Supplement: Supplemental data [file jci-135-183567-s229.pdf]

Supplementary material

Intermittent Ischemia-Reperfusion as a Potent Insulin-Sensitizing Intervention via Blood Flow Enhancement and Muscle Decanoyl-L-carnitine Suppression

Kohei Kido[1,2,3], Janne R. Hingst[1], Johan Onslev[1], Kim A. Sjøberg[1], Jesper B. Birk[1], Nicolas O. Eskesen[1], Tongzhu Zhou[3], Kentaro Kawanaka[3], Jesper F. Havelund[4], Nils J. Færgeman[4], Ylva Hellsten[5], Jørgen F.P. Wojtaszewski[1], Rasmus Kjøbsted[1]

[1] The August Krogh Section for Molecular Physiology, Department of Nutrition, Exercise and Sports, Faculty of Science, University of Copenhagen, DK-2100 Copenhagen, Denmark

[2]Health and Medical Research Institute, National Institute of Advanced Industrial Science and Technology (AIST), Takamatsu, Kagawa, Japan.

[3]Faculty of Sports and Health Science, Fukuoka University, Fukuoka, Japan.

[4]Department of Biochemistry and Molecular Biology, VILLUM Center for Bioanalytical Sciences, University of Southern Denmark, 5230 Odense, Denmark

[5] The August Krogh Section for Human Physiology, Department of Nutrition, Exercise and Sports, Faculty of Science, University of Copenhagen, DK-2100 Copenhagen, Denmark

Supplementary Methods

Analysis of Plasma Samples

Plasma glucose and lactate concentrations were measured by a blood-gas analyzer (ABL800 FLEX, Radiometer, Denmark). Plasma insulin concentration was quantified using an Insulin ELISA kit (ALPCO, Salem, NH). The concentration of plasma fatty acids (NEFA C kit, Wako Chemicals GmbH, Neuss, Germany), triacylglycerol (TG) (GPO-PAP kit, Roche Diagnostics, Rotkreuz, Switzerland) and glycerol (Glycerol assay, Randox Laboratoires, Crumlin, UK) were measured using enzymatic colorimetric methods (Pentra 400, Clinical chemistry analyzer, Horiba GmbH, Wolfsburg, Germany). Plasma adrenalin and noradrenalin were measured using a 2-CAT Plasma ELISAHigh Sensitive kit (Labor Diagnostika Nord GmbH & Co, Nordhorn, Germany).

Human Muscle Processing

The frozen muscle biopsy samples were freeze-dried for 48 h and subsequently dissected free of visible connective tissue, fat and blood. The freeze-dried and dissected muscle samples were homogenized as previously described (1) and muscle protein lysates were collected as the supernatant of centrifuged (16,000g, 20 min, 4°C) muscle protein homogenates. Total muscle protein content in lysate and homogenate was determined by the bicinchoninic acid method (Pierce BCA protein assay kit, Thermo Fisher Scientific, Waltham, MA).

SDS-PAGE and Western Blot Analyses

Muscle protein lysate and homogenate were boiled in laemmli buffer and separated by SDS-PAGE using self-cast polyacrylamide gels (5-12%). After SDS-PAGE, the proteins were transferred to polyvinylidene difluoride (PVDF) membranes by the semidry blotting method. The membranes were blocked for 5 min in 2% fat-free skim milk with Tris-buffered saline containing 0.05% Tween 20 (TBS-T) followed by overnight incubation at 4°C with primary antibodies

against AMPK α2 (Santa Cruz, Dallas, TX #sc-19131), Hexokinase II (Santa Cruz, Dallas, TX #sc-130358 or Cell Signaling Technology, Danvas, MA #2867) ; TBC1D4 (Abcam, Cambridge, UK, # ab189890); GLUT4 (Thermo Scientific, Waltham, MA, PA1-1065 or Cell Signaling Technology, Danvas, MA #2213); eNOS (# 610296), p-eNOS Ser^{1177} (#612393)(BD Transduction Lab); TBC1D1 (Proteintech, Rosemont, IL, #22124-1-AP); p-TBC1D1 Ser^{237} (Millipore, Burlington, MA, #07-2268); p-TBC1D4 Ser^{704} (Yenzym antibodies, Brisbane, CA, custom made); pyruvate dehydrogenase (PDH), phospho-PDH site1, and phospho-PDH site2 (Dr. Henriette Pilegaard, University of Copenhagen, Copenhagen, custom made), glycogen synthase (GS) (Dr. Oluf Pedersen, University of Copenhagen, Copenhagen, custom made); p-GS Ser^{7+10} (GS site2+2a), and p-GS $Ser^{640+644}$ (GS site3a+3b) (Dr. David Grahame Hardie, University of Dundee, Dundee, custom made); Akt2 (#3063 or #2920), Akt Ser^{473} (#9271 or #4060) Akt Thr^{308} (#9275 or #13038), p-TBC1D4 Thr^{642} (#8881), p-TBC1D4 Ser^{588} (#8730), acetyl-CoA carboxylase (ACC) Ser^{221} (#3661), and Hexokinase I (#2024) (Cell Signaling Technology, Danvas, MA). The probed membranes were washed with TBS-T and incubated with appropriate secondary antibodies for 1 h at room temperature. After a final wash in TBS-T, the protein bands were visualized using chemiluminescence (Immobilon Western HRP, Millipore, Burlington, MA) and a ChemiDoc MP imaging system (BioRad Laboratories, Hercules, CA).

Muscle Glycogen and Metabolites

Glycogen concentration in skeletal muscle was measured in 150 µg of muscle protein homogenate after acid hydrolysis. Briefly, muscle protein homogenates were heated at 100°C for 2 hours in 2 M HCl, and then glycosyl units were quantified in the supernatant by a fluorometric method (2). Muscle lactate, creatine (Cr), phosphocreatine (PCr), and adenine nucleotides were

extracted in percloric acid and measured fluorometrically (2), while nucleotides were analyzed by reverse-phase HPLC (3).

AMPK, GS, and HK activity

AMPK α1β2γ1, α2β2γ1, and α2β2γ3, heterotrimer-specific activity was measured by sequential immunoprecipitation (IP) of the AMPKγ3, α2 and α1 subunits. AMPK γ3, α2 (Custom made; MRC PPU Reagents and Services, University of Dundee, Scotland, UK), and α1 antibody (Custom made; Genscript, NJ, USA) were used for IP. The isoform-specific AMPK activity was measured in the presence of 200 µM AMP and 100 µM AMARA-peptide (Schafer-N, Copenhagen, Denmark) as previously described (4). GS activity was measured in homogenates in the presence of 0.02, 0.17, and 8 mM glucose-6-phosphate (G6P) as previously described (5). HK activity was determined by rapid radiochemical filter paper assay as previously described (6).

Muscle Metabolomics Analysis

Ice-cold extraction solvent (methanol:acetonitrile:water (5:3:2)) was added to pre-weighed tissues (250 µL per 25 mg tissue) and tissues were then lysed by beating in a FastPrep-24 homogenizer. Blank samples without tissue were included as blank controls. Subsequently, samples were thoroughly vortexed and centrifuged (16,000 g, 20 min, 4°C). Supernatants were transferred to new 1.5 mL Eppendorf tubes prior to lyophilization. Samples were then resuspended in 0.01 % formic acid (25 µL), centrifuged again, and transferred to LC injection vials. 5 µL from each sample were pooled and used for quality control (QC).

Samples were injected using a Vanquish Horizon UPLC (Thermo Fisher Scientific) equipped with Zorbax Eclipse Plus C18 guard (2.1 × 50 mm and 1.8 µm particle size, Agilent Technologies), and an analytical column (2.1 × 150 mm and 1.8 µm particle size, Agilent Technologies) kept at 40°C. The analytes were eluted using a flow rate of 400 µL/min and the

following composition of eluent A (0.1% formic acid) and eluent B (0.1% formic acid, acetonitrile) solvents: 3% B from 0 to 1.5 min, 3% to 40% B from 1.5 to 3 min, 40% to 95% B from 3 to 5 min, 95% B from 5 to 7.6 min and 95% to 3% B from 7.6 to 8 min before equilibration for 3.5 min with the initial conditions. The flow from the UPLC was coupled to a QExactive mass spectrometer (Thermo) for mass spectrometric analysis, operated in both positive and negative ion mode.

Raw data were processed with MzMine (7). Compounds were annotated at metabolomics standards initiative (8) level 2 using local MS/MS spectra databases of the National Institute of Standards and Technology 17 (NIST17) and MassBank of North America (MoNA).

Features were removed if their average signal were not > 5 x more abundant in the QC samples than blanks (water extraction). The signals were corrected for drift using statTarget (9). Finally, signals were normalized using the QC samples, before log transformation (base 10) and auto scaling, all done in Metaboanalyst (10).

Carnitine and Acylcarnitine Analysis

Muscle tissue samples (approx. 8 mg) were extracted using one-phase and the extraction solvent MeOH:ACN:H2O (50:30:20). Prior to tissue lysis, Splash mix (Merck) was added to the extraction solvent, and tissue samples were lysed by beat beating in a FastPrep-24 homogenizer. After shaking at 4°C (1,000 rpm, 30 min), samples were centrifuged (16,000 g, 15 min, 4°C) and the supernatant transferred to new tubes. Supernatants were lyophilized and resuspended in 0.1% formic acid and transferred to LC vials. A quality control sample was constructed by pooling 3 µl of each sample.

Samples were analyzed using a Vanquish Horizon UPLC system (Thermo Fisher Scientific) with two separate injections per sample: one optimized for short-chain acyl carnitines

and another for long-chain acyl carnitines. For short-chain acyl carnitines, the setup included a Zorbax Eclipse Plus C18 guard column (2.1 × 50 mm, 1.8 µm particle size; Agilent Technologies, Santa Clara, CA, USA) and an analytical column (2.1 × 150 mm, 1.8 µm particle size; Agilent Technologies) maintained at 40°C. The mobile phase consisted of eluent A (0.1% formic acid in water) and eluent B (0.1% formic acid in acetonitrile) at a flow rate of 400 µL/min, with the following gradient: 3% B (0–1.5 min), 3–40% B (1.5–4.5 min), 40–95% B (4.5–7.5 min), 95% B (7.5–10.1 min), 95–3% B (10.1–10.5 min), followed by 3.5 min equilibration at initial conditions. For long(er)-chain acyl carnitines, a Waters ACQUITY Premier CSH column (2.1 × 100 mm, 1.7 µm particle size) was used at 55°C. The mobile phase comprised eluent A (60:40 acetonitrile/water, 10 mM ammonium formate, 0.1% formic acid) and eluent B (90:10 isopropanol/acetonitrile, 10 mM ammonium formate, 0.1% formic acid) at 400 µL/min, with the gradient: 40% B (0–0.5 min), 40–43% B (0.5–0.7 min), 43–65% B (0.7–0.8 min), 65–70% B (0.8–2.3 min), 70–99% B (2.3–6 min), 99% B (6–6.8 min), 99–40% B (6.8–7 min), followed by 3 min equilibration at initial conditions.

The UPLC effluent was coupled to a TimsTOF Flex (Bruker) mass spectrometer operated in positive ion mode in a scanning range from 40-1300 m/z. Compounds were annotated in Metaboscape (Bruker, v 2025B) using a rule-based annotation approach and the LipidBlast MS2 library (11). Features with an average signal intensity in quality control (QC) samples less than 3-fold higher than in blanks (water extraction) were excluded. When an acyl carnitine was detected in both setups, the setup yielding the lowest coefficient of variation (CV) in the QC sample pool was selected. Signals were corrected for drift and filtered using blanks and QC samples with Metabolink software (12).

References

1. Hingst JR, et al. Exercise-induced molecular mechanisms promoting glycogen supercompensation in human skeletal muscle. *Mol Metab.* 2018;1624-1634.
2. Lowry HO, et al. A flexible system of enzymatic analysis. *London: Academic Press, Inc.* 1972.
3. Tullson PC, et al. Alterations in AMP deaminase activity and kinetics in skeletal muscle of creatine kinase-deficient mice. *Am J Physiol.* 1998;274(5):C1411-C1416.
4. Birk JB, Wojtaszewski JFP. Kinase Activity Determination of Specific AMPK Complexes/Heterotrimers in the Skeletal Muscle. *Methods Mol Biol.* 2018;1732:215-228.
5. Højlund K, et al. Dysregulation of glycogen synthase COOH- and NH2-terminal phosphorylation by insulin in obesity and type 2 diabetes mellitus. *J Clin Endocrinol Metab.* 2009;94(11):4547-4556.
6. Hingst JR, et al. Rapid radiochemical filter paper assay for determination of hexokinase activity and affinity for glucose-6-phosphate. *J Appl Physiol (1985).* 2019;127(3):661–667.
7. Pluskal T, et al. MZmine 2: modular framework for processing, visualizing, and analyzing mass spectrometry-based molecular profile data. *BMC Bioinformatics.* 2010;11:395.
8. Salek RM, et al. Dissemination of metabolomics results: role of MetaboLights and COSMOS. *Gigascience.* 2013;2(1):8.
9. Luan H, et al. statTarget: A streamlined tool for signal drift correction and interpretations of quantitative mass spectrometry-based omics data. *Anal Chim Acta.* 2018;1036:66-72.
10. Chong J, et al. Using MetaboAnalyst 4.0 for Comprehensive and Integrative Metabolomics Data Analysis. *Curr Protoc Bioinformatics.* 2019;68(1):e86.
11. Kind T, et al. LipidBlast in silico tandem mass spectrometry database for lipid identification. *Nat Methods.* 2013;10(8):755–758.
12. Mendes A, et al. MetaboLink: a web application for streamlined processing and analysis of large-scale untargeted metabolomics data. *Bioinformatics.* 2024;40(7):btae459.

Supplemental Figure 1.

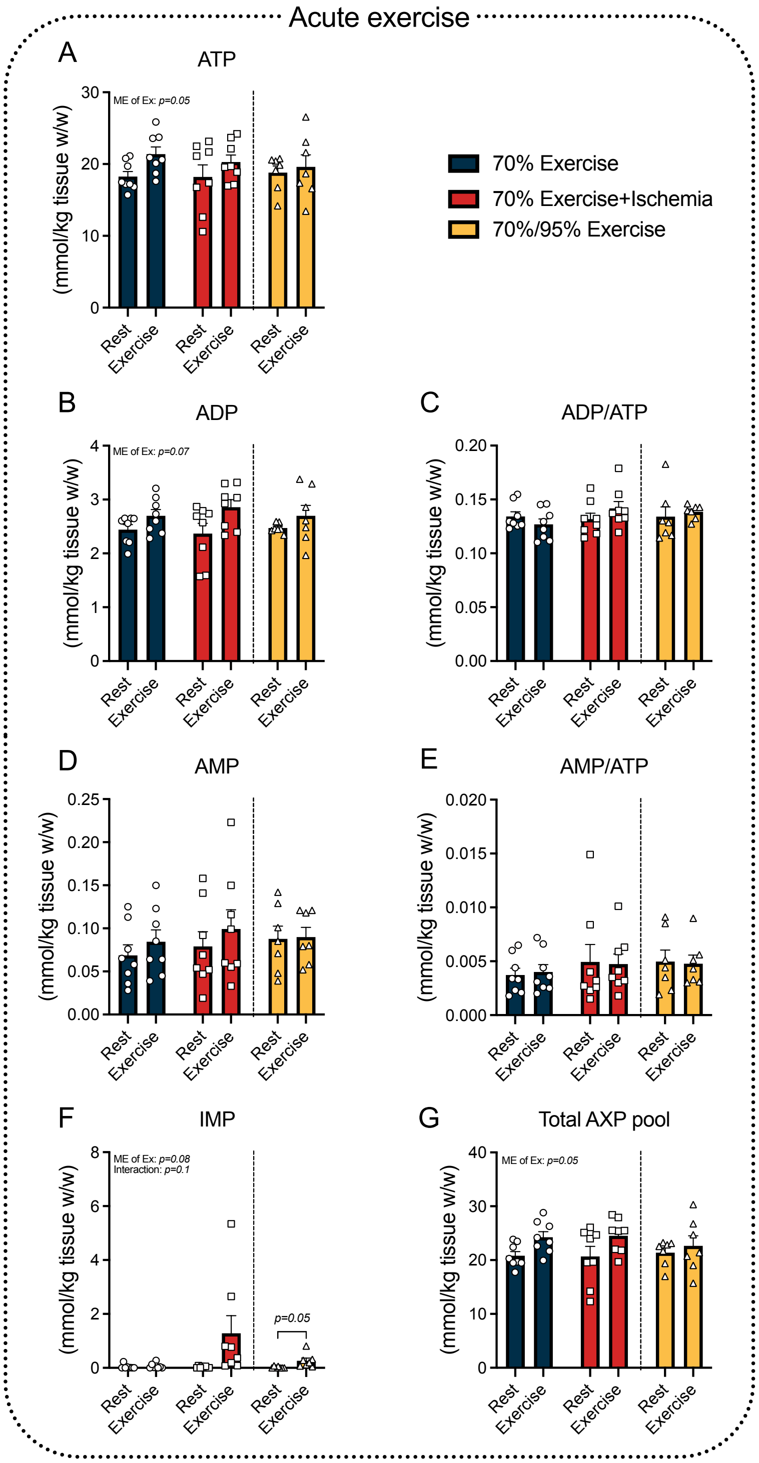

Effect of exercise on intramuscular nucleotide levels.
The figures illustrate the content of ATP (A), ADP (B), ADP/ATP ratio (C), AMP (D), AMP/ATP ratio (E), IMP (F), and total nucleotides (AXP) (G) in muscle before and immediately after exercise. n = 8 70% Exercise and 70% Exercise+ischemia and n = 7 70%/95% Exercise. Data are mean ± SEM.

For panels A-G, comparisons between 70% Ex and 70% Ex + Ischemia were performed using 2-way repeated-measures ANOVA. For 70%/95% Ex, the exercise effect was evaluated using a paired (two-tailed) Student's t-test.
ME, main effect.

Supplemental Figure 2.

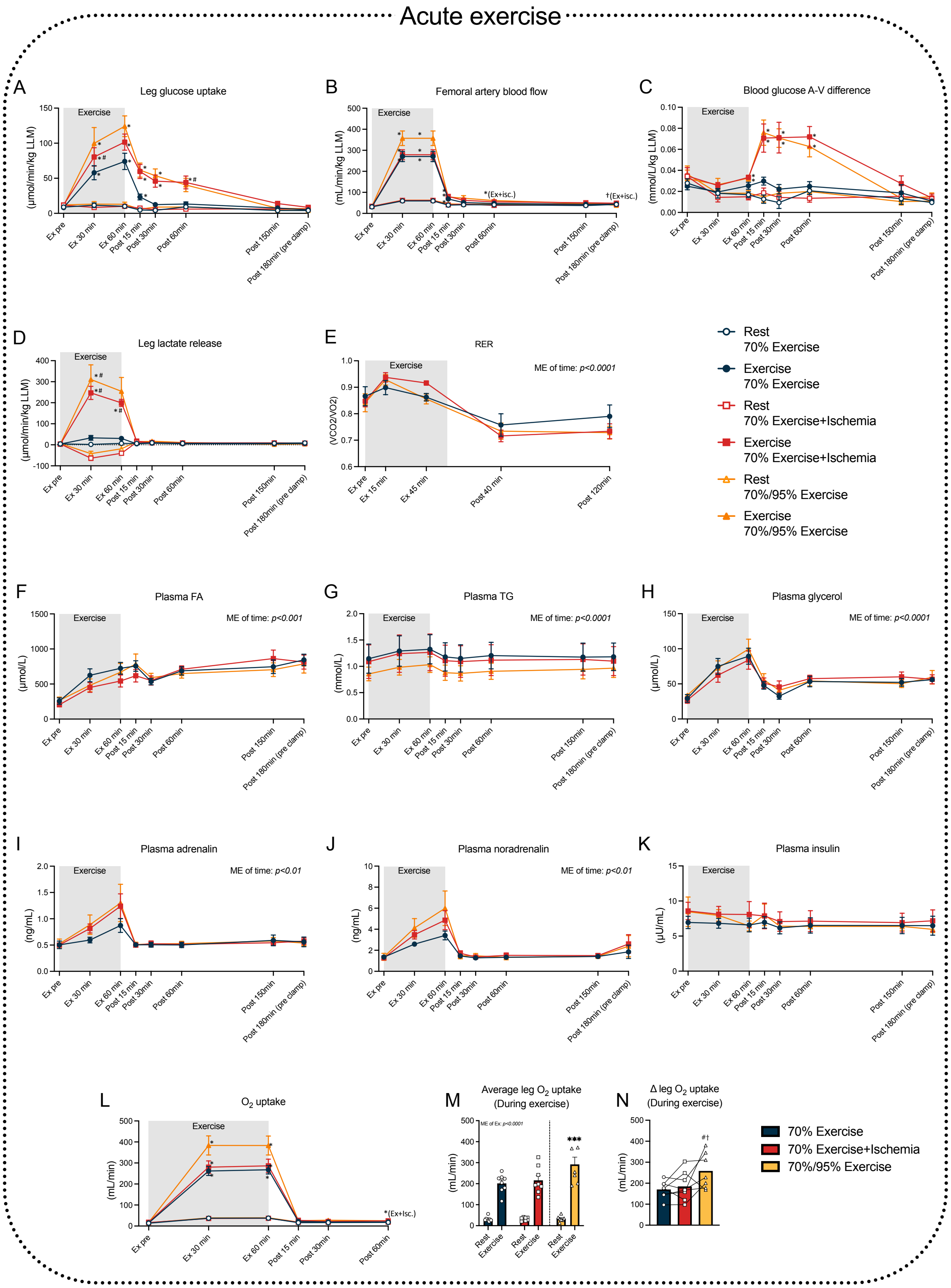

Effect of exercise on circulatory and metabolic parameters during and after acute exercise.

The figures illustrate the time-course changes in leg and blood parameters before, during (shaded area), and after acute 70% Exercise (black), 70% Exercise+Ischemia (red), and 70%/95% Exercise (orange). The following variables are shown:(A) Leg glucose uptake, (B) Leg blood flow, (C) Arteriovenous (A–V) glucose difference, (D) Lactate release, (E) Respiratory exchange ratio (RER), (F) Plasma free fatty acids (FA), (G) Plasma triglycerides (TG), (H) Plasma glycerol, (I) Plasma adrenaline, (J) Plasma noradrenaline, (K) Plasma insulin, (L) Leg oxygen uptake. The figures depict the average leg O^2 uptake during each exercise(M), along with the changes (Δ) induced by each exercise (N).
n = 8 for 70% Exercise and 70% Exercise+Ischemia; n = 7 for 70%/95% Exercise. Data are mean ± SEM. *$P < 0.05$, ***$P < 0.001$ vs. Rest within each condition; #$P < 0.05$ vs. 70% Exercise; †$P < 0.05$ vs. 70% Exercise+Ischemia.
For Panels A-L, a 2-way repeated-measures ANOVA was used, followed by Tukey’s post hoc test when appropriate. For panel M, comparison between 70% Ex and 70% Ex + Ischemia was performed using 2-way repeated-measures ANOVA. For 70%/95% Ex, the exercise effect was evaluated using a paired (two-tailed) Student’s t-test. For panel N, a 1-way repeated-measures ANOVA was used, followed by Tukey’s post hoc test when significance was found.
ME, main effect.

Supplemental Figure 3.

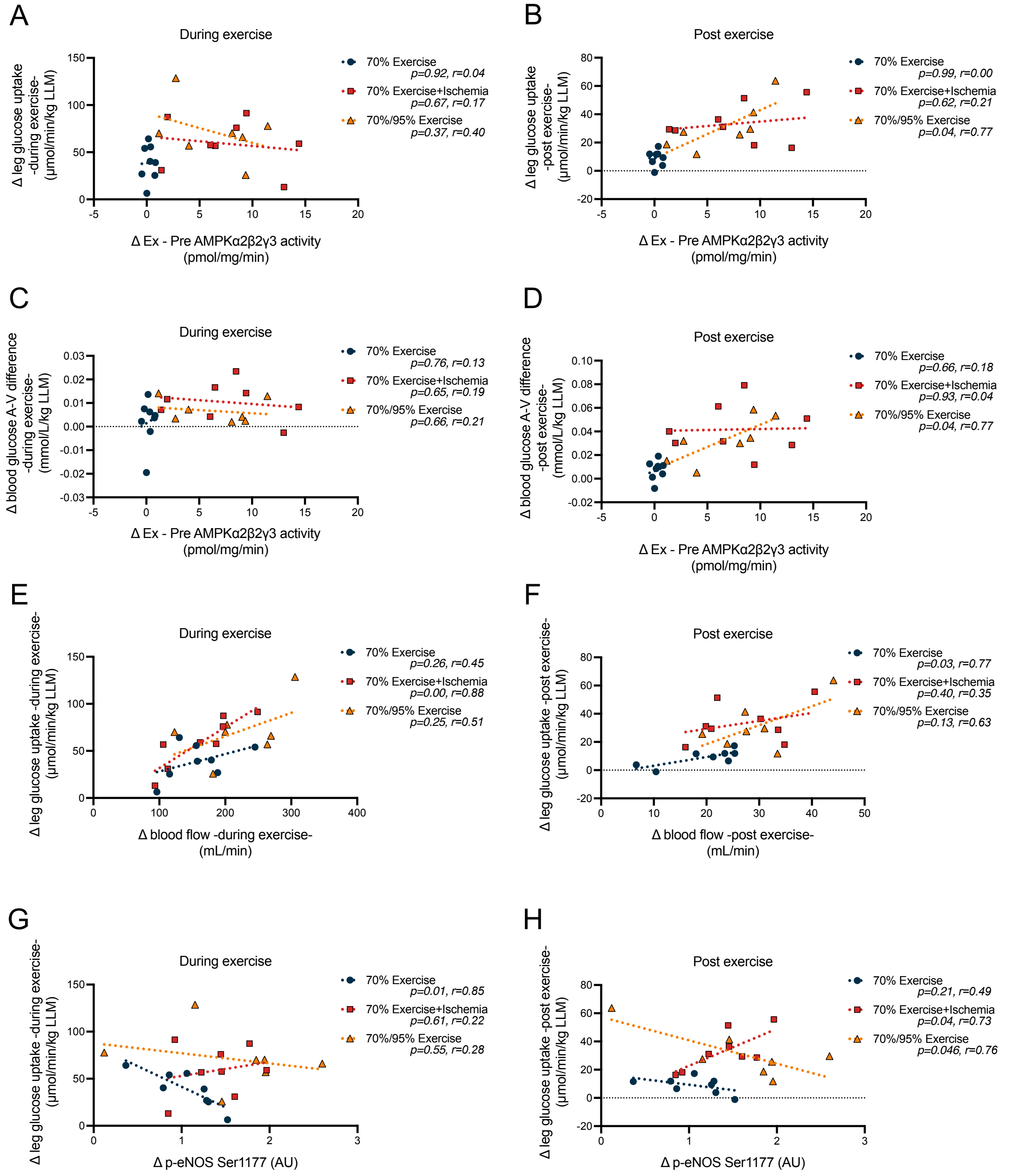

Relationships between AMPK activity, leg blood flow, eNOS phosphorylation, and leg glucose uptake during and after acute exercise.

The figures illustrate the correlations between exercise-induced changes in molecular and physiological parameters during and after three exercise modalities: 70% Exercise (black), 70% Exercise+Ischemia (red), and 70%/95% Exercise (orange). The following relationships are shown: (A, B) Leg glucose uptake vs. change in AMPKα2β2γ3 activity (Δ Ex - Pre) during (A) and post (B) exercise. (C, D) Arteriovenous (A–V) glucose difference vs. change in

AMPKα2β2γ3 activity during (C) and post (D) exercise. (E, F) Leg glucose uptake vs. change in leg blood flow during (E) and post (F) exercise. (G, H) Leg glucose uptake vs. change in eNOS Ser1177 phosphorylation during (G) and post (H) exercise. Each data point represents an individual participant. Linear regression lines are shown for each condition with corresponding Pearson's correlation coefficients (r) and p-values. n = 8 for 70% Exercise and 70% Exercise+Ischemia; n = 7 for 70%/95% Exercise. Data are shown as individual values with trend lines. Correlation analyses were performed using Pearson's product–moment correlation coefficient separately for each intervention: 70% Ex, 70% Ex + Ischemia, and 70%/95% Ex.

Supplemental Figure 4.

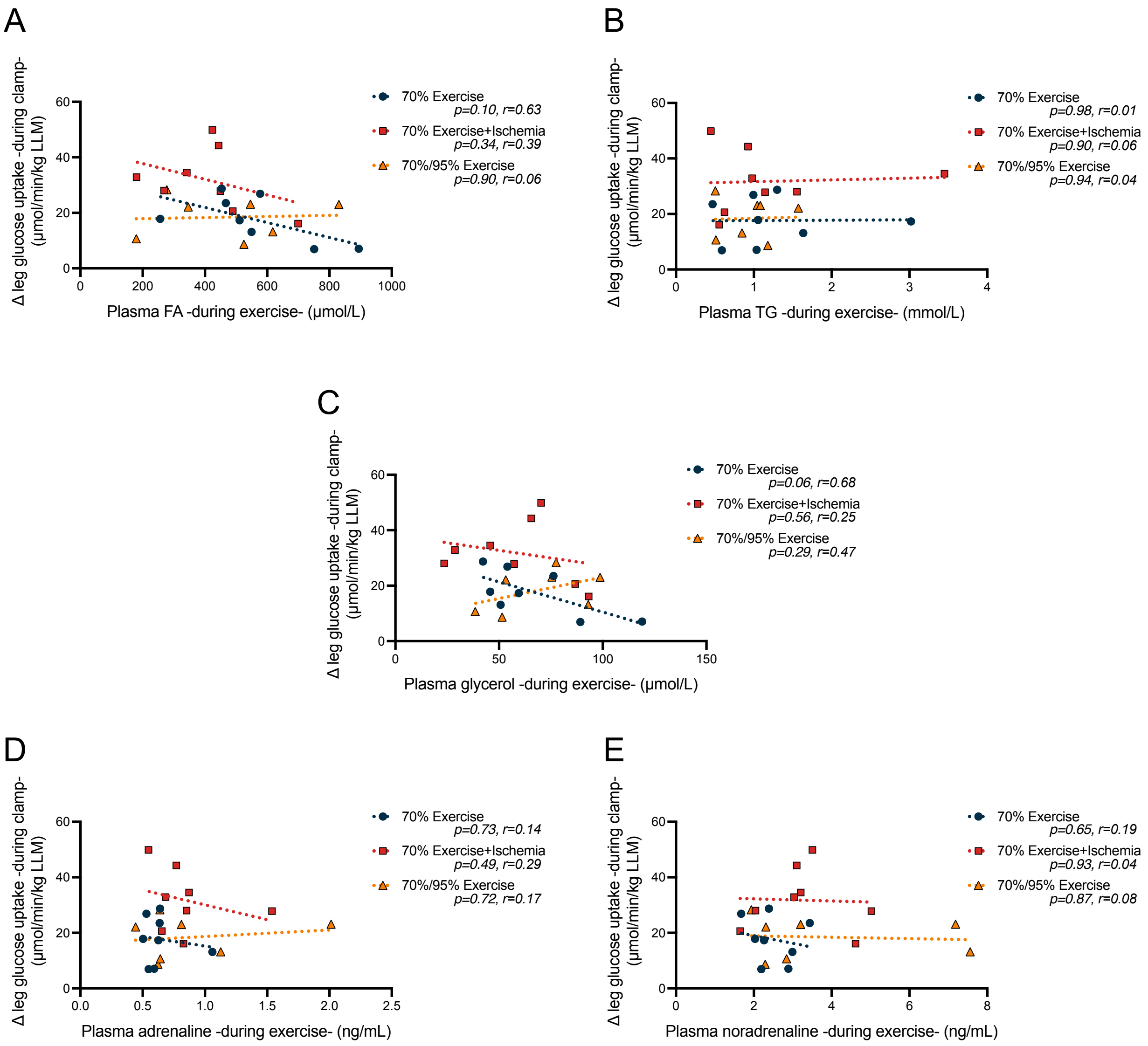

Relationships between lipid-related plasma metabolites, catecholamines, and leg glucose uptake during acute exercise.

The figures illustrate the correlations between plasma metabolite or hormone concentrations and leg glucose uptake during the clamp phase of acute exercise under three conditions: 70% Exercise (black), 70% Exercise+Ischemia (red), and 70%/95% Exercise (orange). The following relationships are shown: (A) Plasma free fatty acids (FA) vs. leg glucose uptake, (B) Plasma triglycerides (TG) vs. leg glucose uptake, (C) Plasma glycerol vs. leg glucose uptake, (D) Plasma adrenaline vs. leg glucose uptake, (E) Plasma noradrenaline vs. leg glucose uptake. Each point represents an individual participant. Linear regression lines are shown for each condition, along with corresponding Pearson's correlation coefficients (r) and p-values. n = 8 for 70% Exercise and 70% Exercise+Ischemia; n = 7 for 70%/95% Exercise. Data are shown as individual values with trend lines. Correlation analyses were performed using Pearson's product–moment

correlation coefficient separately for each intervention: 70% Ex, 70% Ex + Ischemia, and 70%/95% Ex.

Supplemental Figure 5.

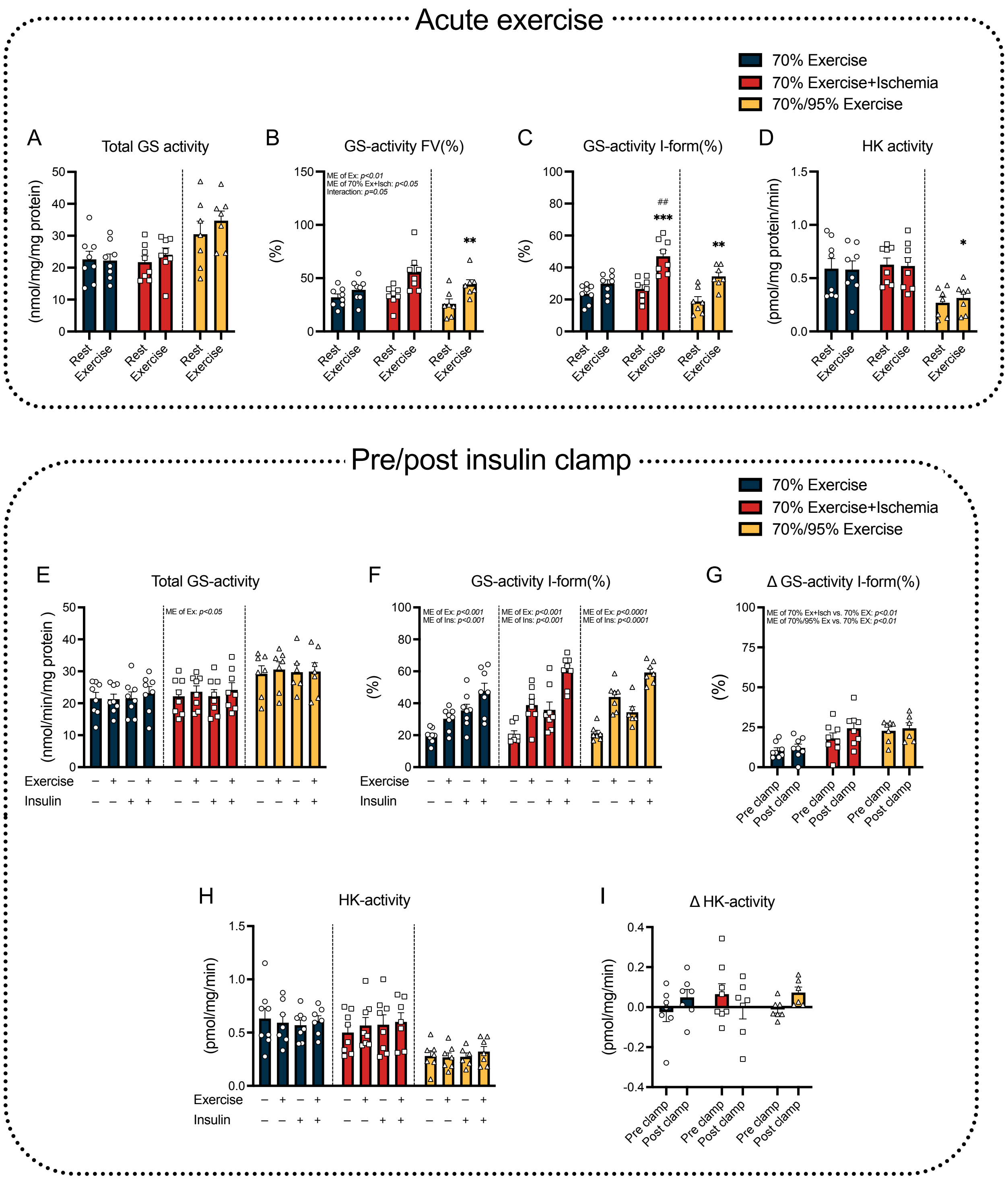

GS and HK activity before and after exercise as well as before and after insulin stimulation.
Depicted in the upper panels are the total GS activity (A), GS activity presented as a percentage of fractional velocity (B), I-form GS activity (C), and hexokinase (HK) activity (D) in both pre-

and immediately post-exercised muscle. Illustrated in the lower panels are the total GS activity (E), GS activity presented as I-form GS activity (F), and HK activity (H) before and after the insulin clamp, along with the alterations (Δ) resulting from each prior exercise (G, I). n = 8 70% Exercise and 70% Exercise+ischemia and n = 7 70%/95% Exercise. Data are mean ± SEM.
For panels A-D, comparisons between 70% Ex and 70% Ex + Ischemia were performed using 2-way repeated-measures ANOVA; when a significant interaction was detected, post hoc Bonferroni–Sidak tests were applied. For 70%/95% Ex, the exercise effect was evaluated using a paired (two-tailed) Student's t-test. Panels E, F, and H were analysed using 2-way repeated-measures ANOVA for each intervention (70% Ex, 70% Ex + Ischemia, and 70%/95% Ex). For panel G and I, 2-way repeated-measures ANOVA was used, followed by Tukey's post hoc test when appropriate.
*P < 0.05, **P < 0.01, ***P < 0.001 vs. Rest within each condition; ##P < 0.01 vs. 70% Exercise. ME, main effect.

Supplemental Figure 6.

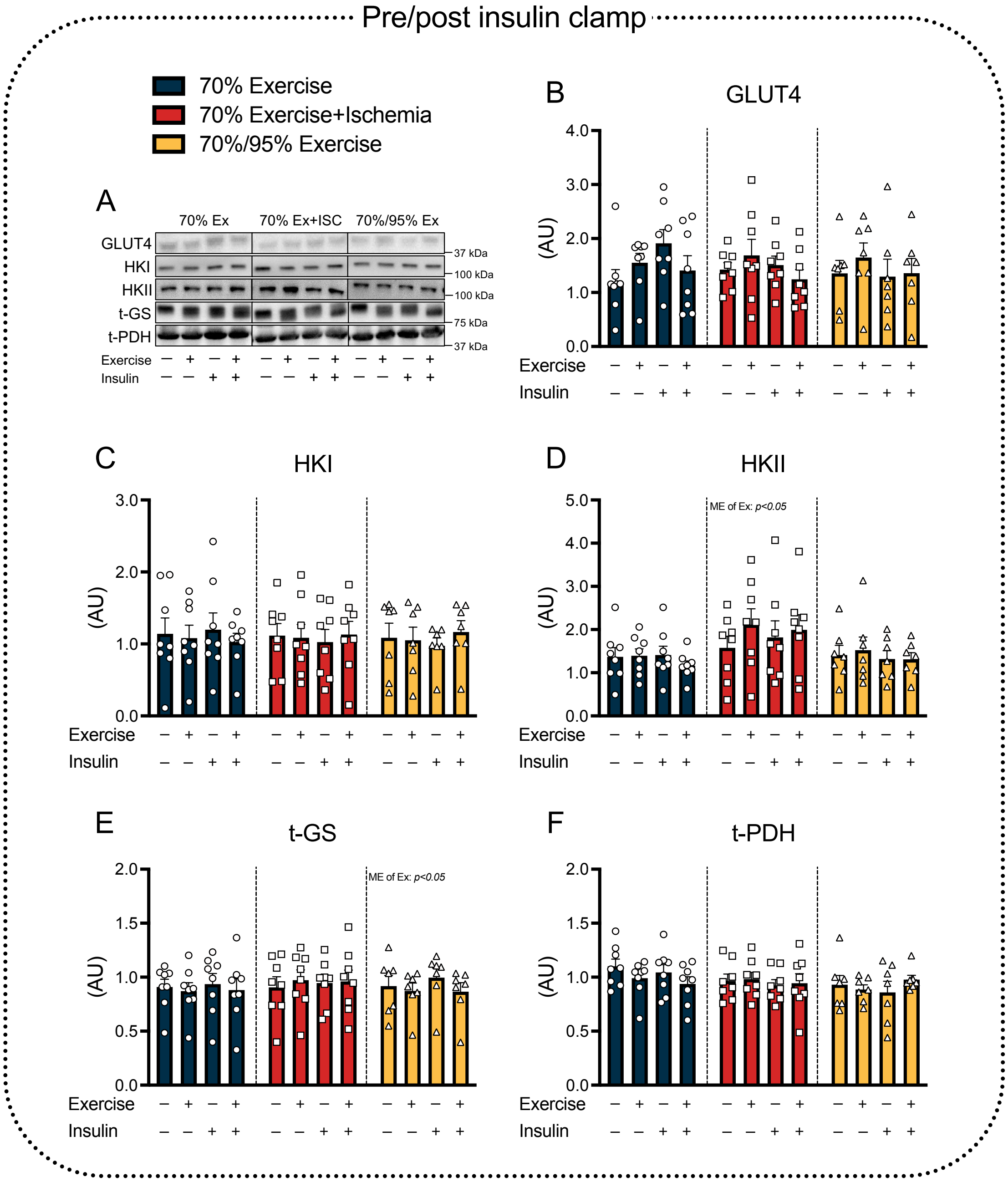

Expression of glucose handing intermediate proteins before and after the insulin clamp. Panel A shows representative immunoblot band images. Illustrated in panels are GLUT4 (B), HKI (C), HKII (D), GS (E), and PDH (F) expression before and after the insulin clamp. n = 8 70% Exercise and 70% Exercise+ischemia and n = 7 70%/95% Exercise. Data are mean ± SEM. Panels B-F were analysed using 2-way repeated-measures ANOVA for each intervention (70% Ex, 70% Ex + Ischemia, and 70%/95% Ex).
ME, main effect.

Supplemental Figure 7.

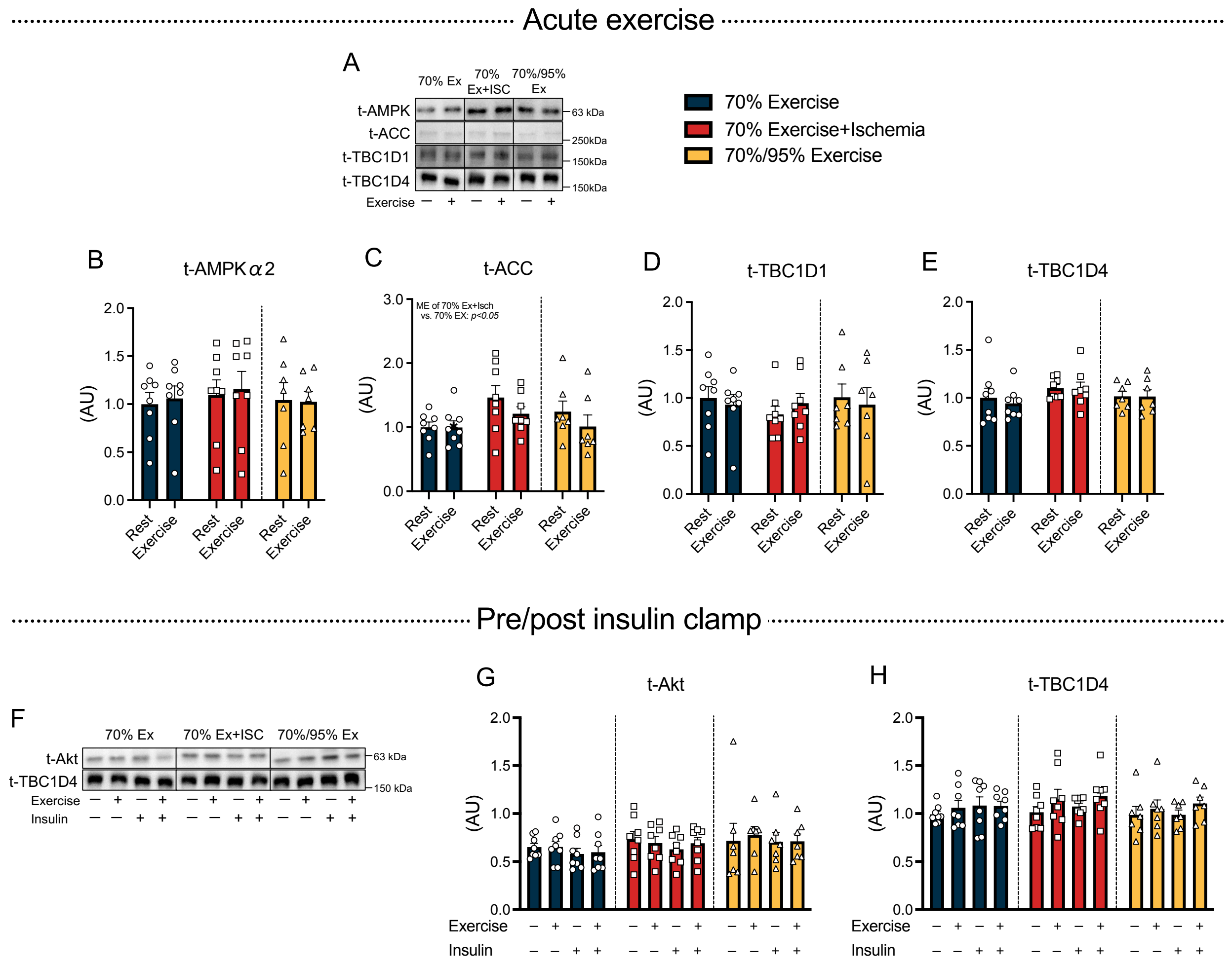

Total expression of glucose uptake regulatory proteins.

Panel A and F display representative immunoblot band images. Shown in the upper panels are the total expressions of pre- and immediately post-exercised AMPKα2 (B), ACC (C), TBC1D1 (D), and TBC1D4 (E). Illustrated in the lower panels are the total expressions of Akt (G) and TBC1D4 (H) before and after the insulin clamp. n = 8 70% Exercise and 70% Exercise+ischemia and n = 7 70%/95% Exercise. Data are mean ± SEM.

For panels B-E, comparisons between 70% Ex and 70% Ex + Ischemia were performed using 2-way repeated-measures ANOVA. For 70%/95% Ex, the exercise effect was evaluated using a paired (two-tailed) Student's t-test. Panels G and H were analysed using 2-way repeated-measures ANOVA for each intervention (70% Ex, 70% Ex + Ischemia, and 70%/95% Ex.

ME, main effect.

Supplemental Table 1. Acute exercise effect on muscle PCr and lactate.

	Resting leg			Exercising leg		
	70% Ex	70% Ex+Ischemia	70%/95% Ex	70% Ex	70% Ex+Ischemia	70%/95% Ex
Muscle						
PCr/ PCr+Cr (mmol/ kg tissue)	0.66 ± 0.02	0.64 ± 0.03	0.60 ± 0.03	0.67 ± 0.02	0.53 ± 0.03 ** #	0.50 ± 0.04 ##
Lactate (mmol/kg tissue)	36.0 ± 3.0	36.4 ± 2.1	30.0 ± 2.3	36.5 ± 1.6	52.7 ± 2.9 * ##	46.9 ± 4.4
Leg lactate release during exercise (μmol/ min/kg LLM)	3.7 ± 1.4	-40.8 ± 6.8 ##	-21.1 ± 7.2 #	25.1 ± 6.1 *	174.0 ± 18.6 *** ####	219.9 ± 46.8 ** #

Data are mean ± SEM. Statistical analysis was performed using 2-way repeated-measures ANOVA followed by Tukey's post hoc test. *P < 0.05, **P < 0.01, and *** P < 0.001 vs. Rest within each trial; # P < 0.05, ## P < 0.01, and #### P < 0.0001 vs. 70% Ex within either rested or exercised leg. PCr, phosphocreatine. Cr, Creatine. LLM, leg lean mass.

Supplemental Table 2. (separate file)

The data used to illustrate Fig. 6A to D and Fig. 7A-B.

Supplemental Table 3. Subject characteristics.

Age (years)	27.3 ± 1.2
Weight (kg)	80.4 ± 3.4
BMI	24.1 ± 1.0
Lean mass (kg)	61.6 ± 2.4
Fat mass (kg)	16.2 ± 2.3
VO_2max (ml min^{-1} kg^{-1})	46.9 ± 1.6
PWL (watt)	56.3 ± 2.5

Data are mean ± SEM. BMI, body mass index. PWL, peak work load.
