# Supplementary material for: Intermittent ischemia/reperfusion as a potent insulin-sensitizing intervention via blood flow enhancement and muscle decanoyl-l-carnitine suppression
Source: J Clin Invest. 2025 Sep 2;135(21):e183567. doi: 10.1172/JCI183567 (PMC12578404; doi:10.1172/JCI183567)
Supplement: Unedited blot and gel images [file jci-135-183567-s230.pdf]

Full unedited gel for Figure 2
P-ACC Ser221

Red dashed line box indicates representative western blot shown in article

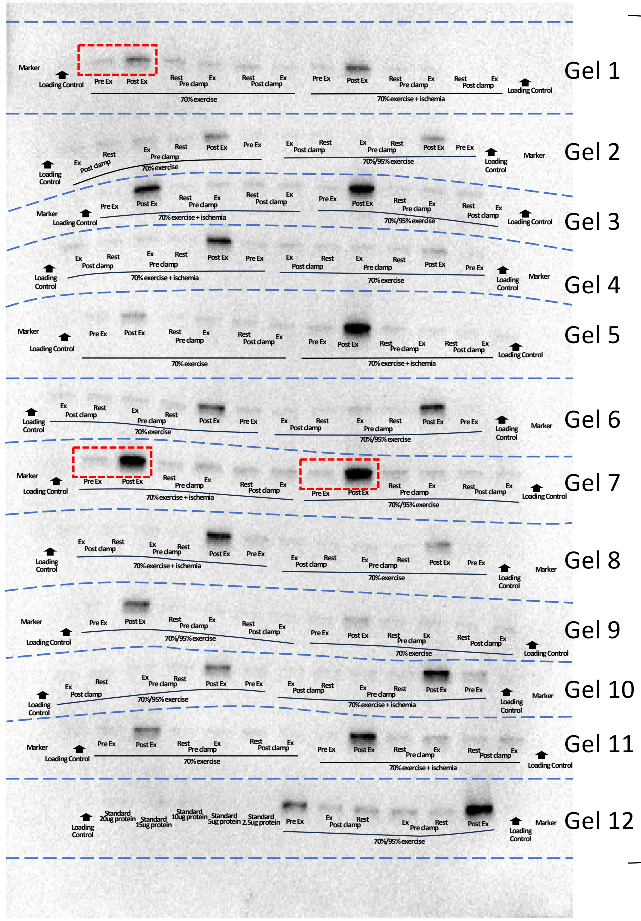

Gel 1
Gel 2
Gel 3
Gel 4
Gel 5
Gel 6
Gel 7
Gel 8
Gel 9
Gel 10
Gel 11
Gel 12

Gel piece 1-12 has been loaded with independent replicates and transferred to a single PVDF membrane

Full unedited gel for Figure 2
P-TBC1D1 Ser237

Red and green dashed line box indicate representative western blot shown in article
The representative western blot within the green dashed box was horizontally flipped in the article to align with the band order as presented.

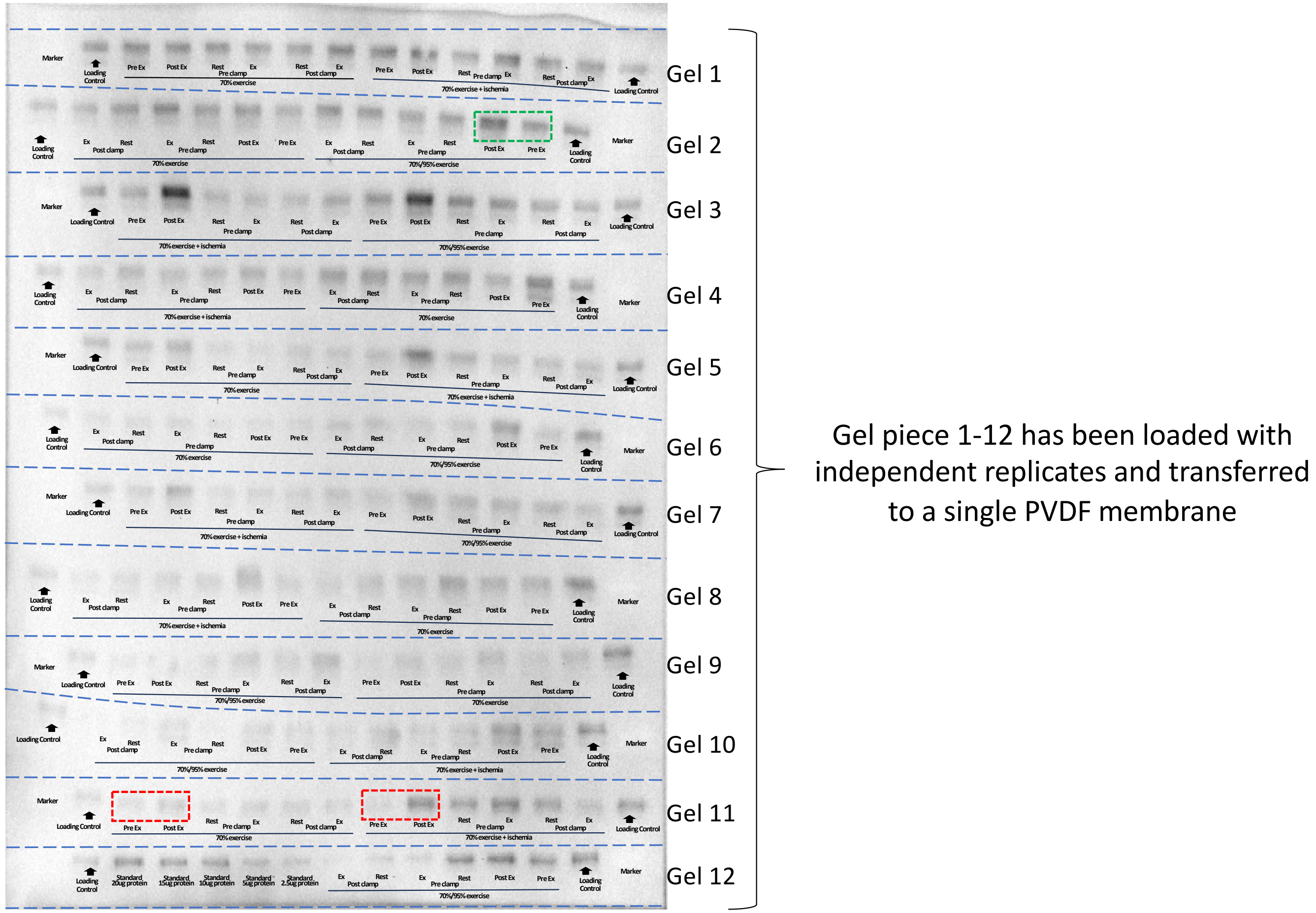

Full unedited gel for Figure 3
P-TBC1D4 Ser704

Red dashed line box indicates representative western blot shown in article

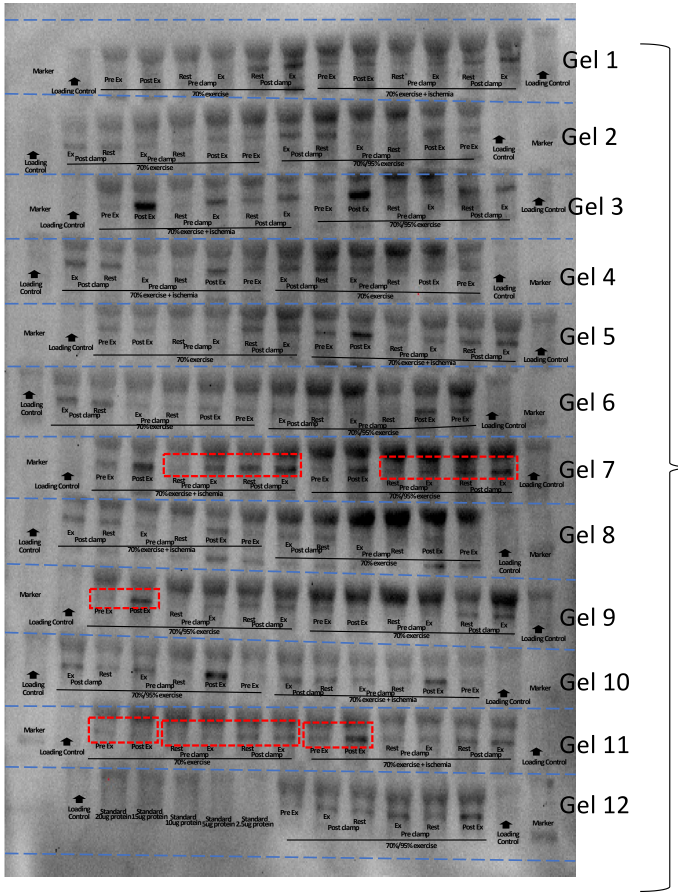

Gel piece 1-12 has been loaded with independent replicates and transferred to a single PVDF membrane

Full unedited gel for Figure 3
P-TBC1D4 Ser588

Red dashed line box indicates representative western blot shown in article

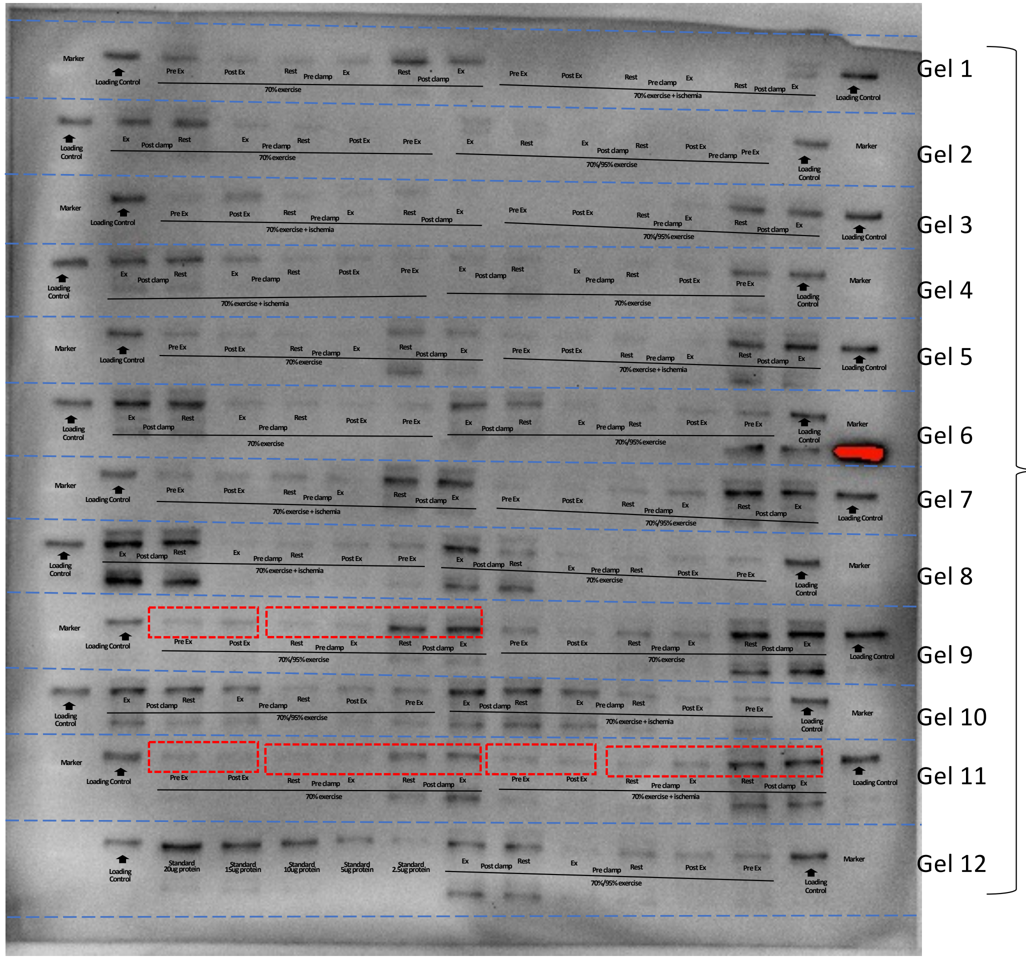

Gel piece 1-12 has been loaded with independent replicates and transferred to a single PVDF membrane

Full unedited gel for Figure 3
P-TBC1D4 Thr642

Red dashed line box indicates representative western blot shown in article

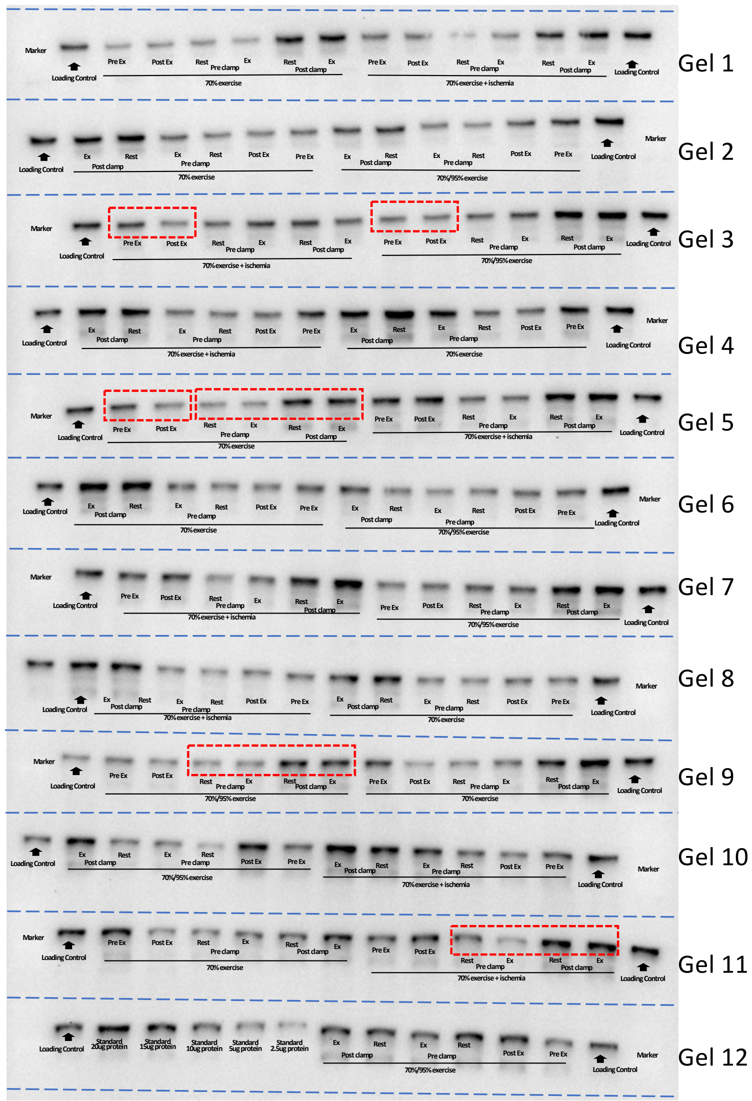

Full unedited gel for Figure 3
P-Akt thr308

Red dashed line box indicates representative western blot shown in article

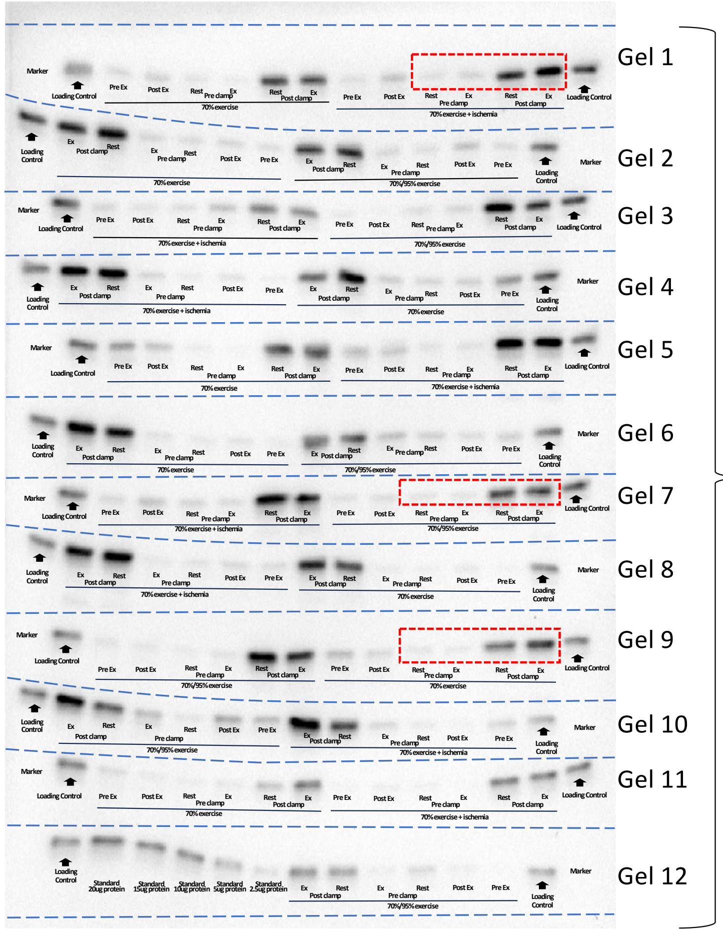

Full unedited gel for Figure 3
P-Akt Ser473

Red dashed line box indicates representative western blot shown in article

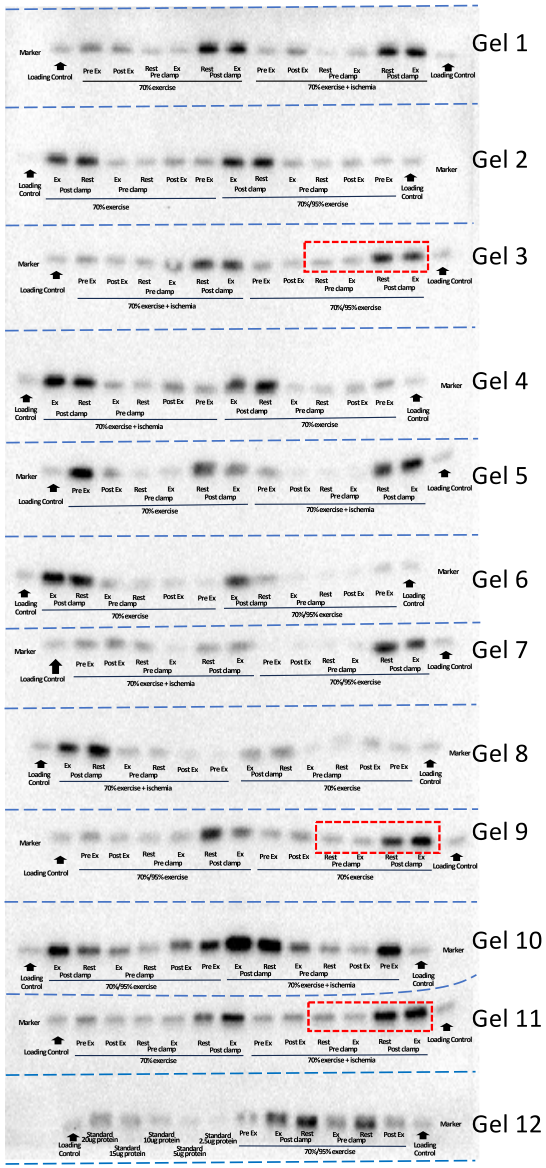

Gel piece 1-12 has been loaded with independent replicates and transferred to a single PVDF membrane

Full unedited gel for Figure 4
P-eNOS Ser1177

Red dashed line box indicates representative western blot shown in article

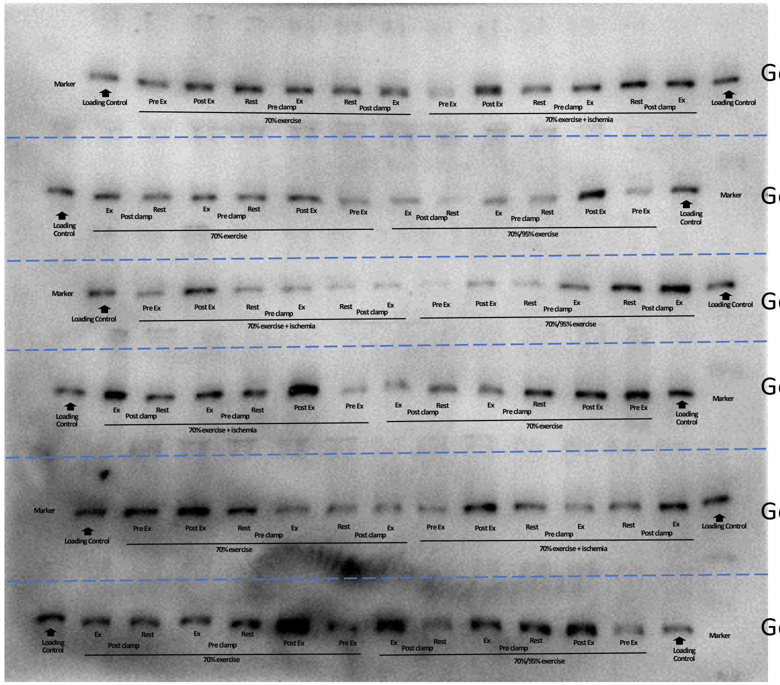

Gel 1
Gel 2
Gel 3
Gel 4
Gel 5
Gel 6

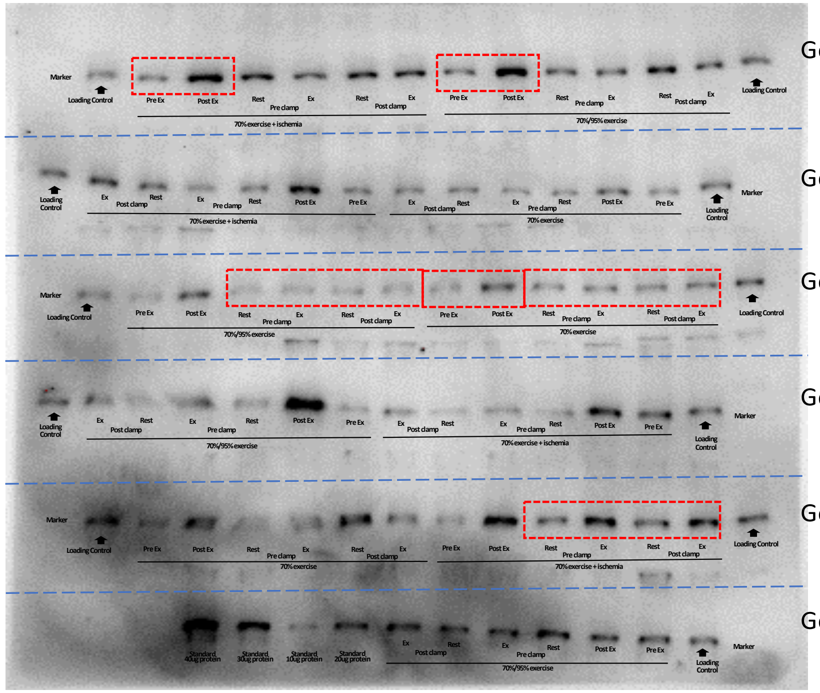

Gel 7
Gel 8
Gel 9
Gel 10
Gel 11
Gel 12

Gel piece 1-12 has been loaded with independent replicates and transferred onto two separate PVDF membranes

Full unedited gel for Figure 4

T-eNOS

Red dashed line box indicates representative western blot shown in article

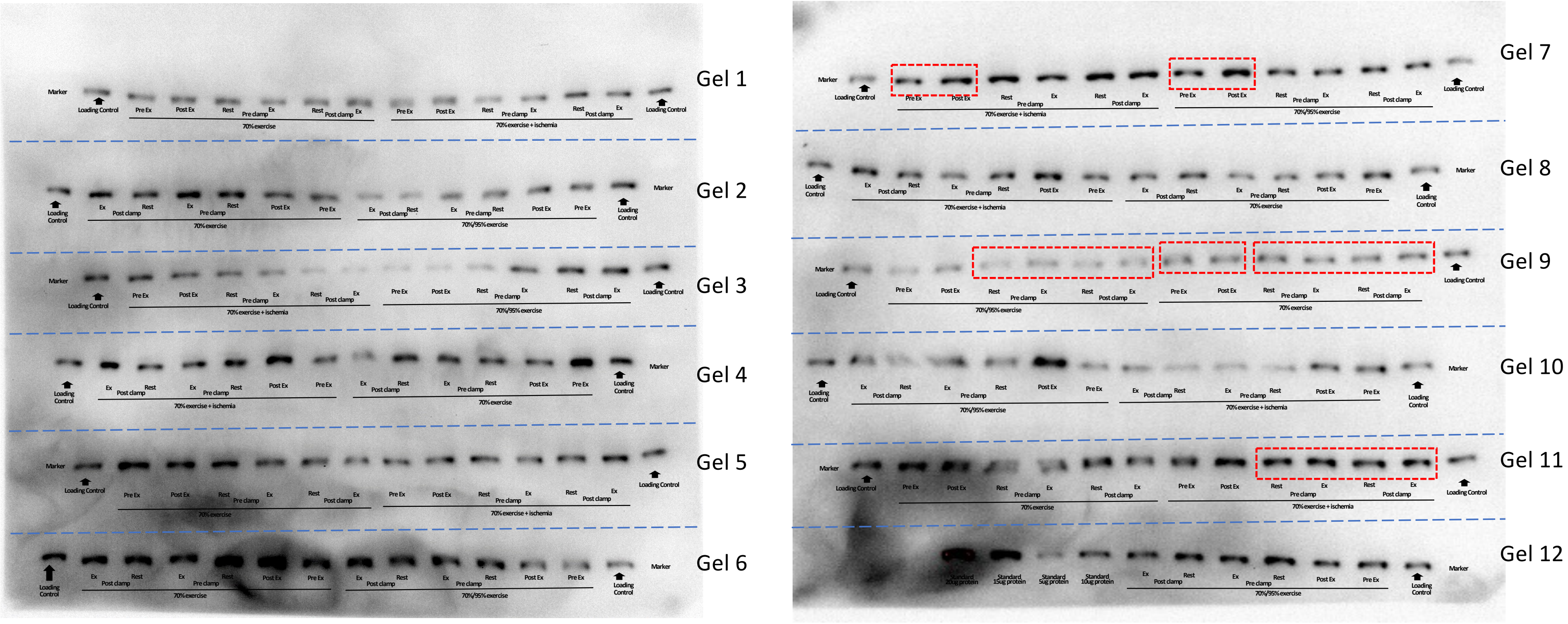

Gel piece 1-12 has been loaded with independent replicates and transferred onto two separate PVDF membranes

Full unedited gel for Figure 5

P-GS sites 2+2a

Red dashed line box indicates representative western blot shown in article

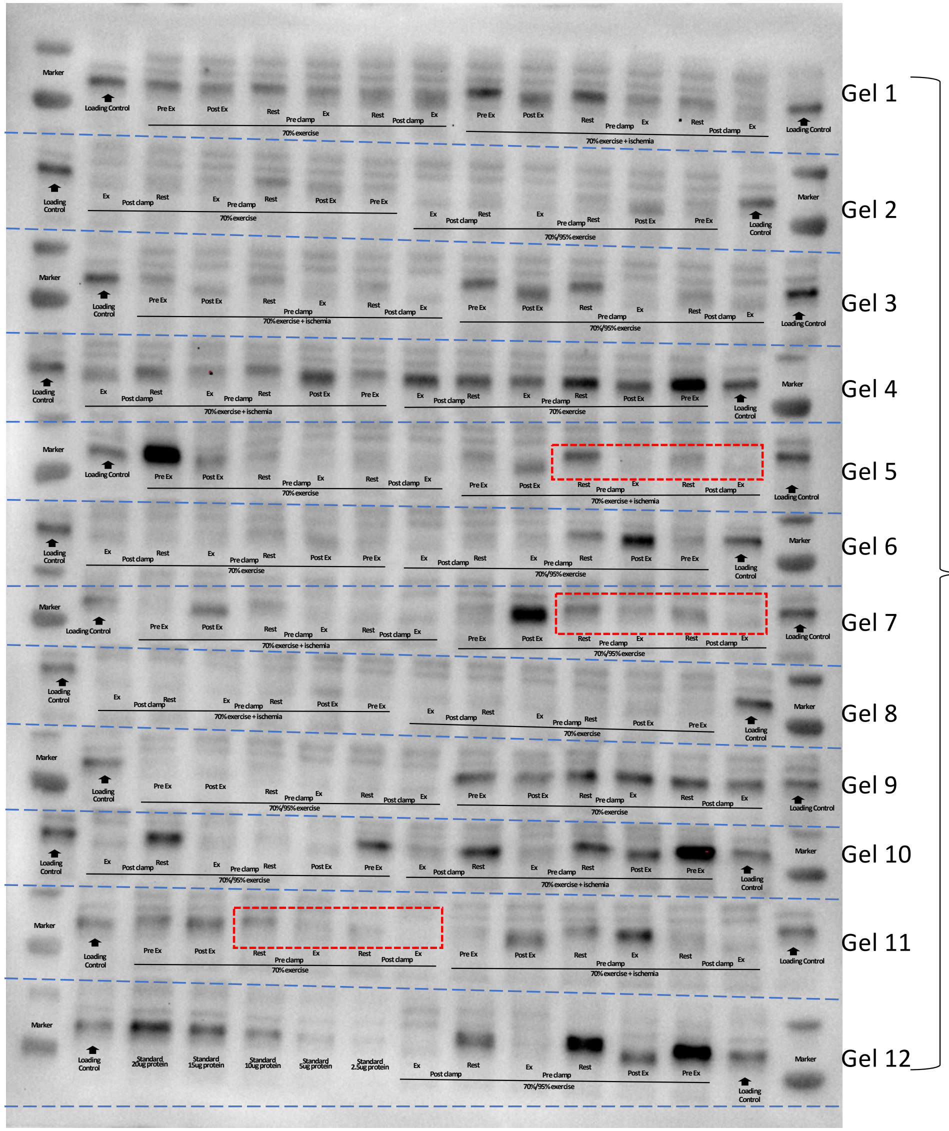

Gel piece 1-12 has been loaded with independent replicates and transferred to a single PVDF membrane

Full unedited gel for Figure 5
P-GS sites 3a+3b

Red dashed line box indicates representative western blot shown in article

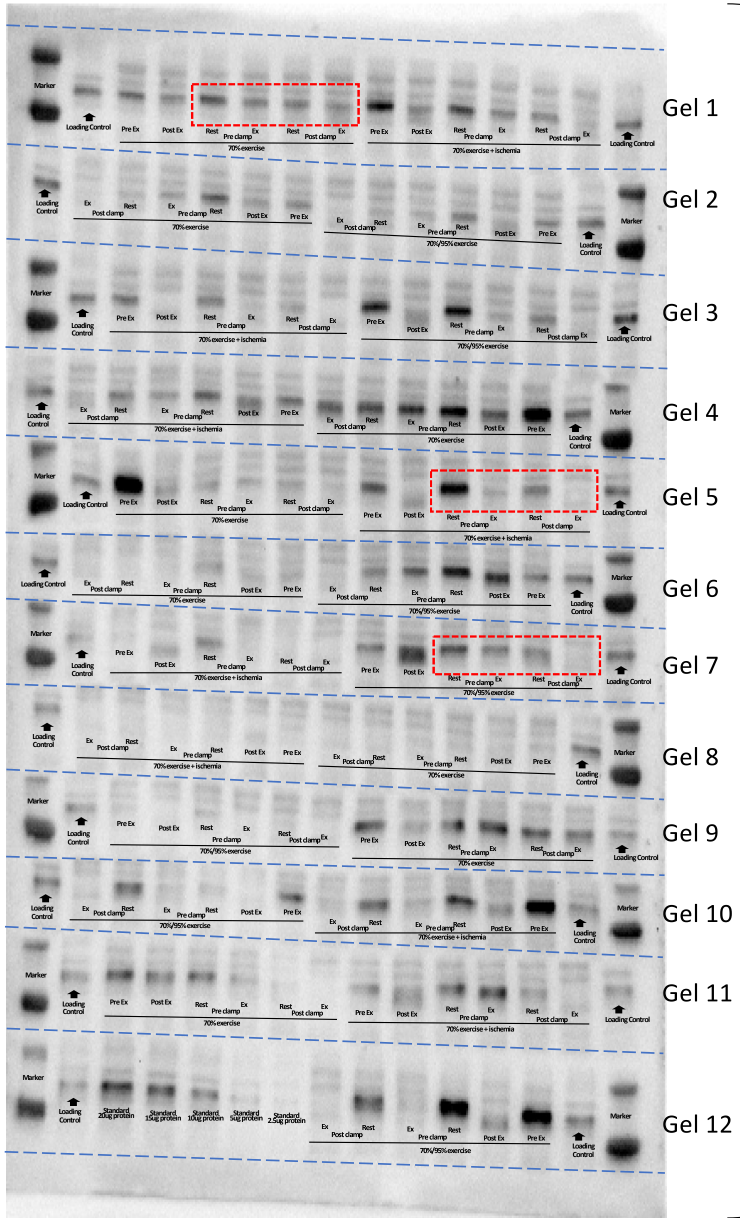

Gel 1
Gel 2
Gel 3
Gel 4
Gel 5
Gel 6
Gel 7
Gel 8
Gel 9
Gel 10
Gel 11
Gel 12

Gel piece 1-12 has been loaded with independent replicates and transferred to a single PVDF membrane

Full unedited gel for Figure 5

P-PDH site 1

Red dashed line box indicates representative western blot shown in article

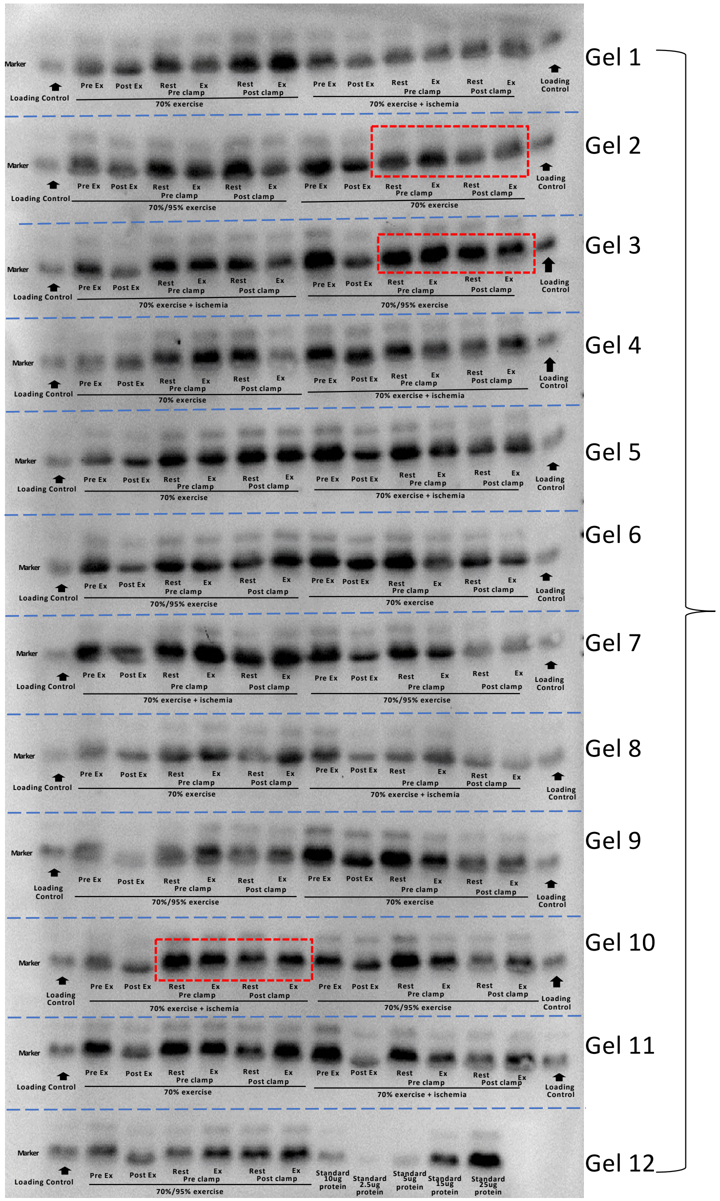

Gel piece 1-12 has been loaded with independent replicates and transferred to a single PVDF membrane

Full unedited gel for Figure 5
P-PDH site 2

Red and green dashed line box indicate representative western blot shown in article
The representative western blot within the green dashed box was horizontally flipped in the article to align with the band order as presented.

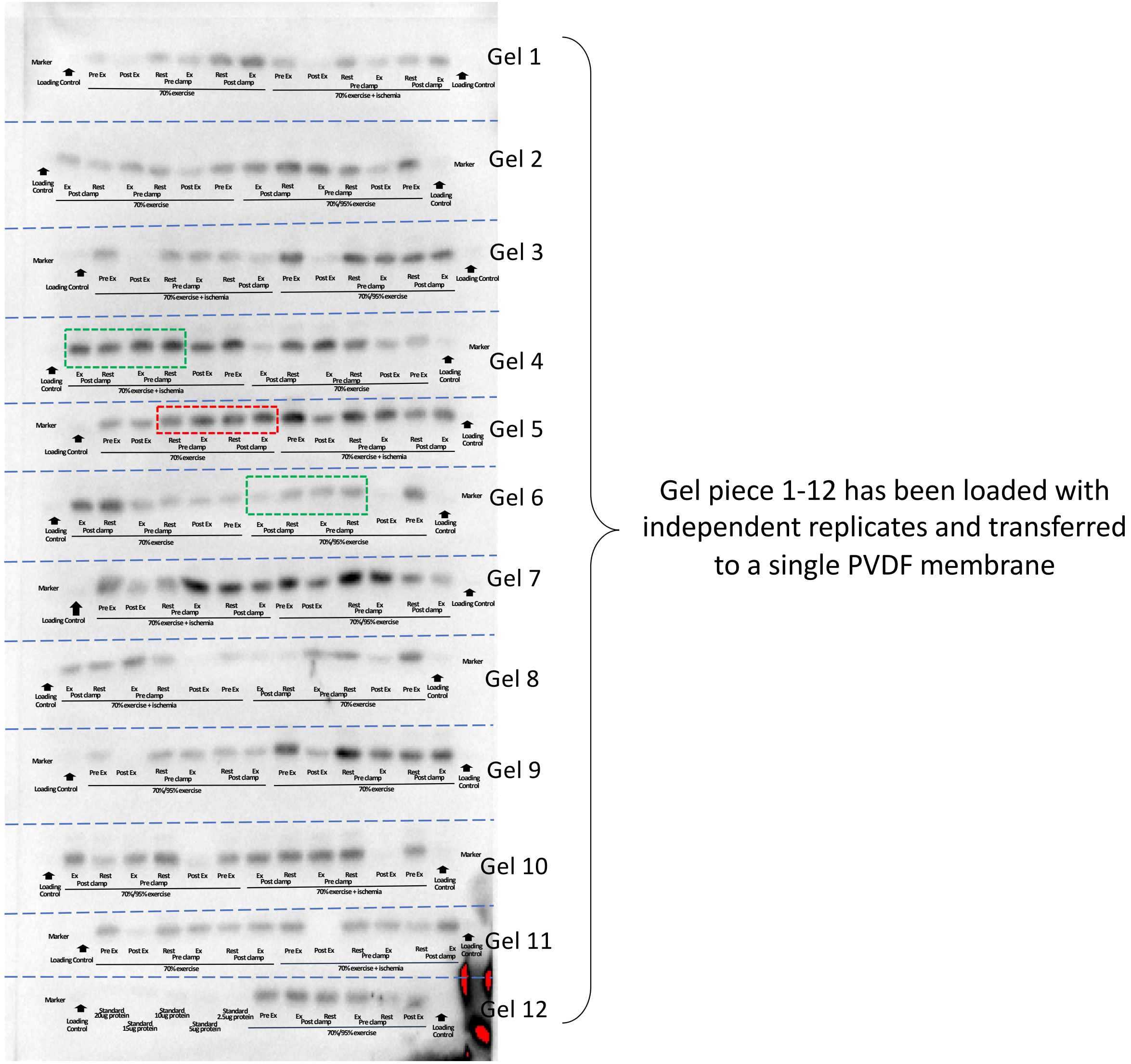

Full unedited gel for Figure 8

P-Akt Thr308

Red dashed line box indicates representative western blot shown in article

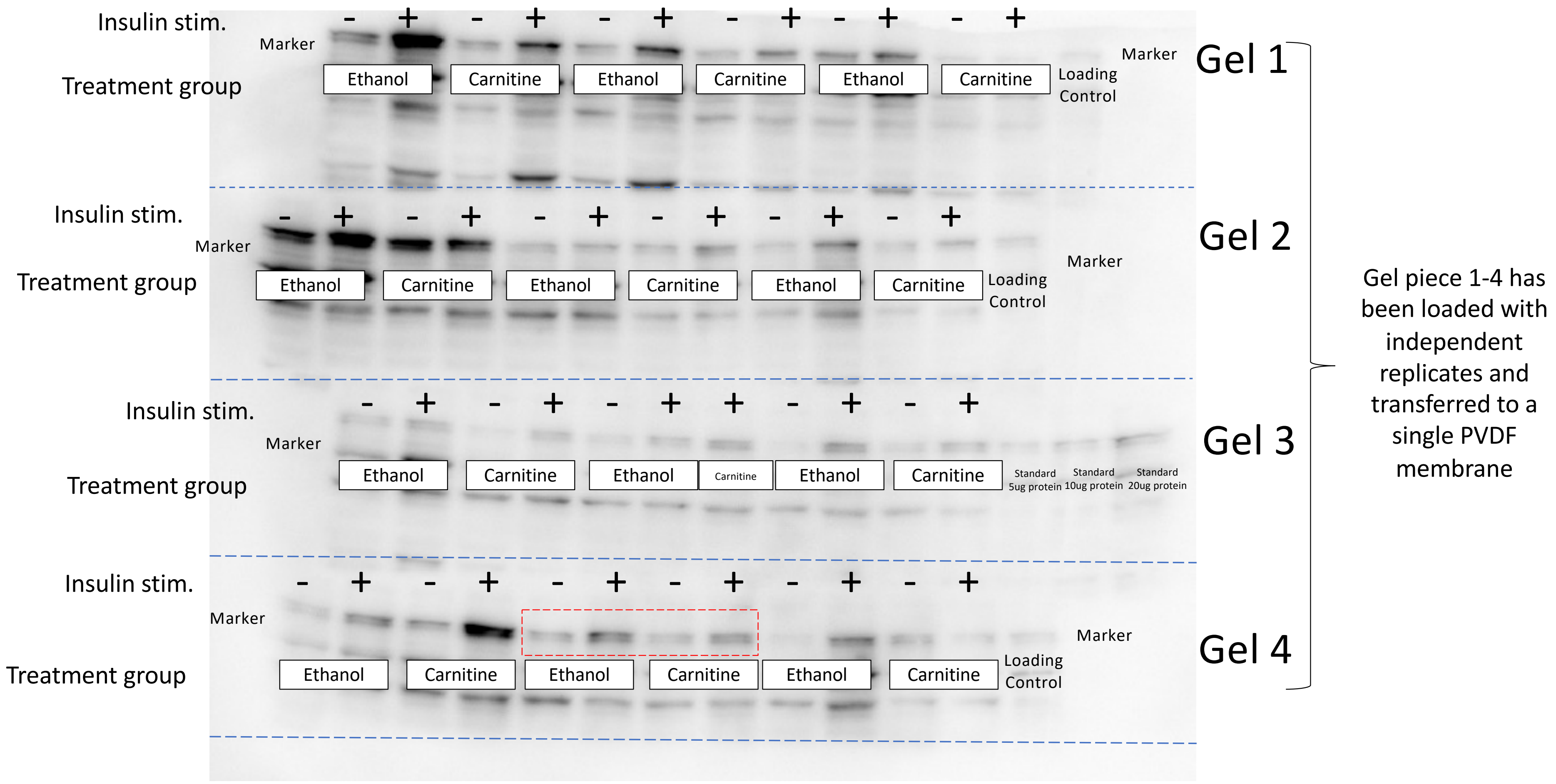

Full unedited gel for Figure 8

P-Akt Ser473

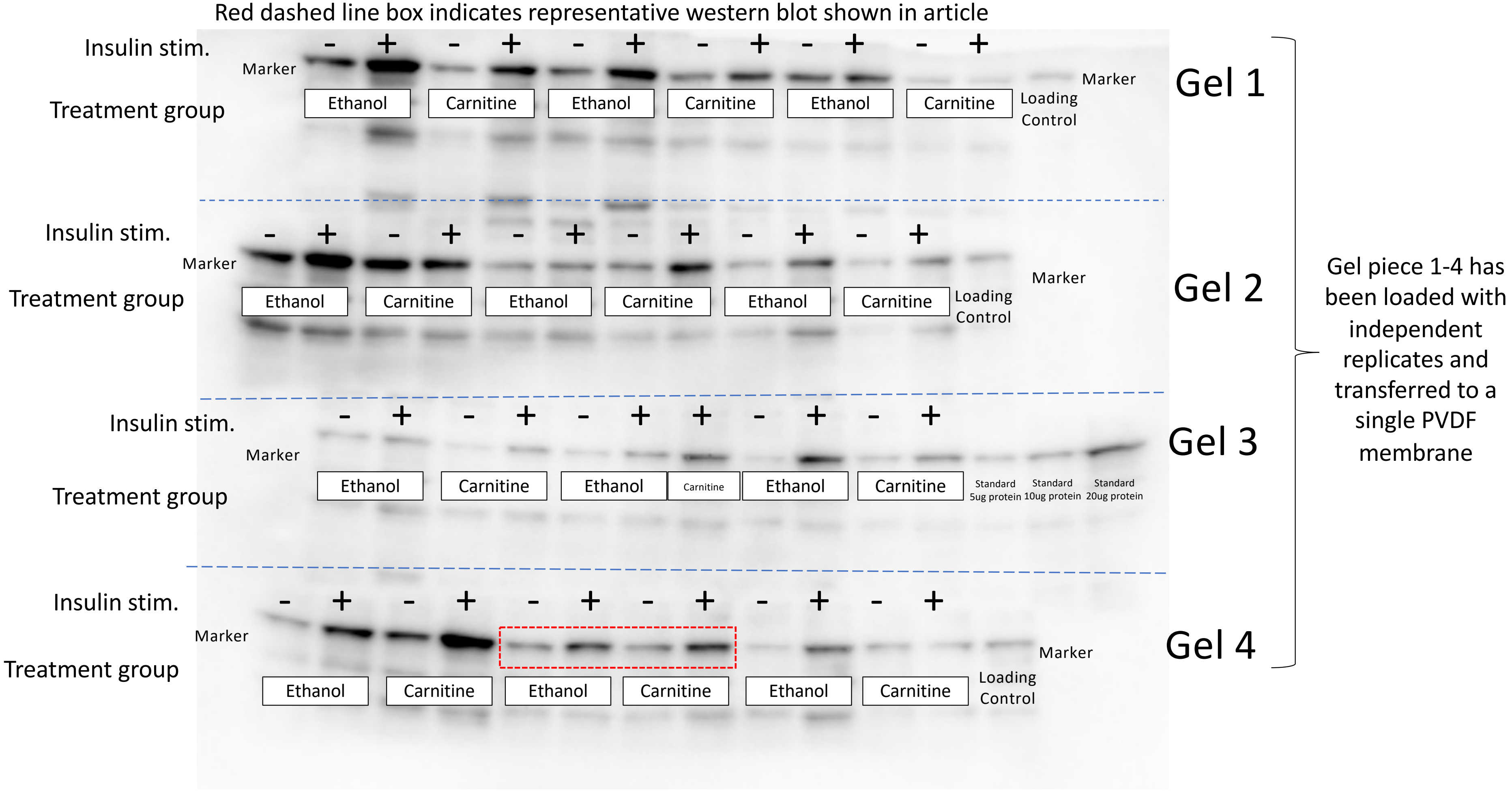

Full unedited gel for Figure 8
T-Akt

Red dashed line box indicates representative western blot shown in article

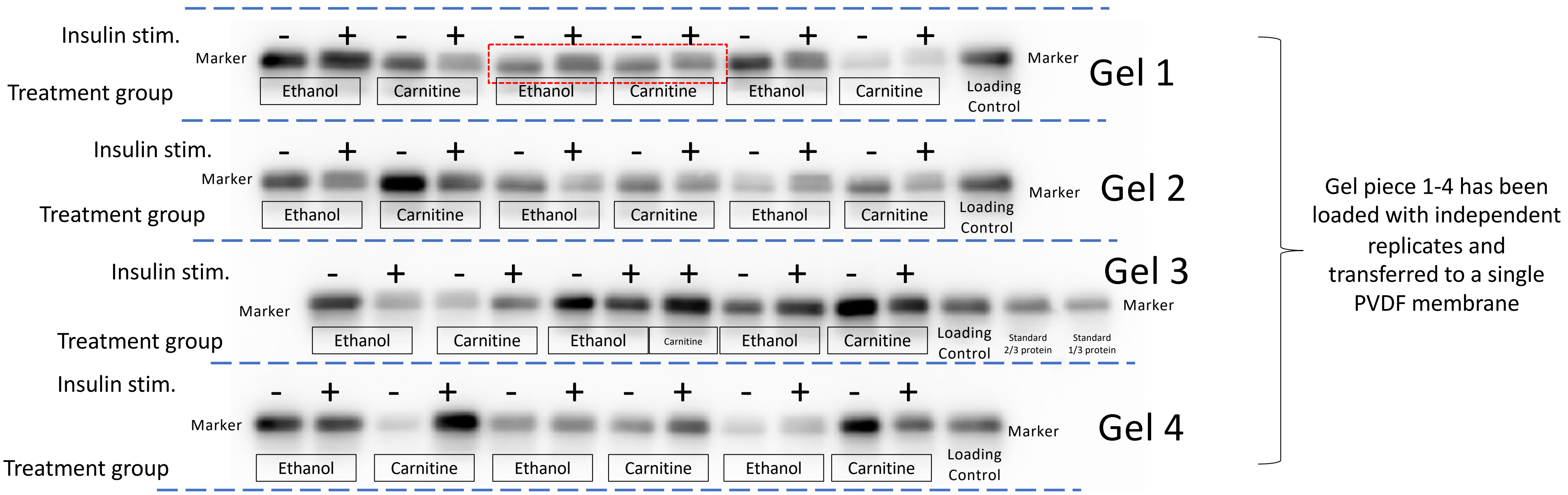

Full unedited gel for Figure 8

GLUT4

Red dashed line box indicates representative western blot shown in article

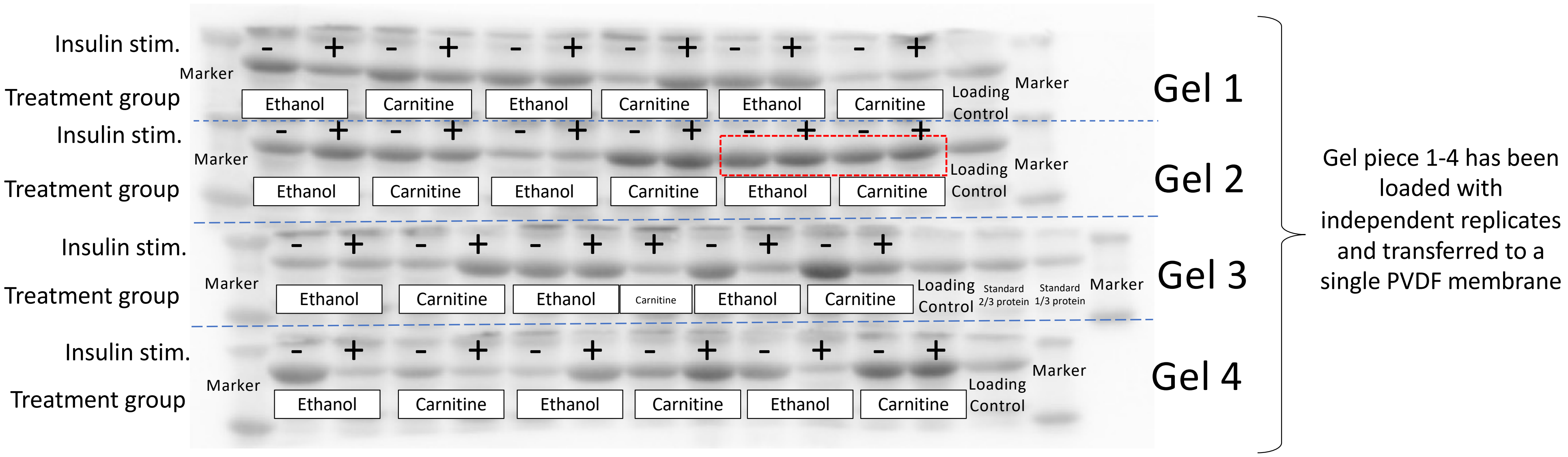

Full unedited gel for Figure 8
HKII

Red dashed line box indicates representative western blot shown in article

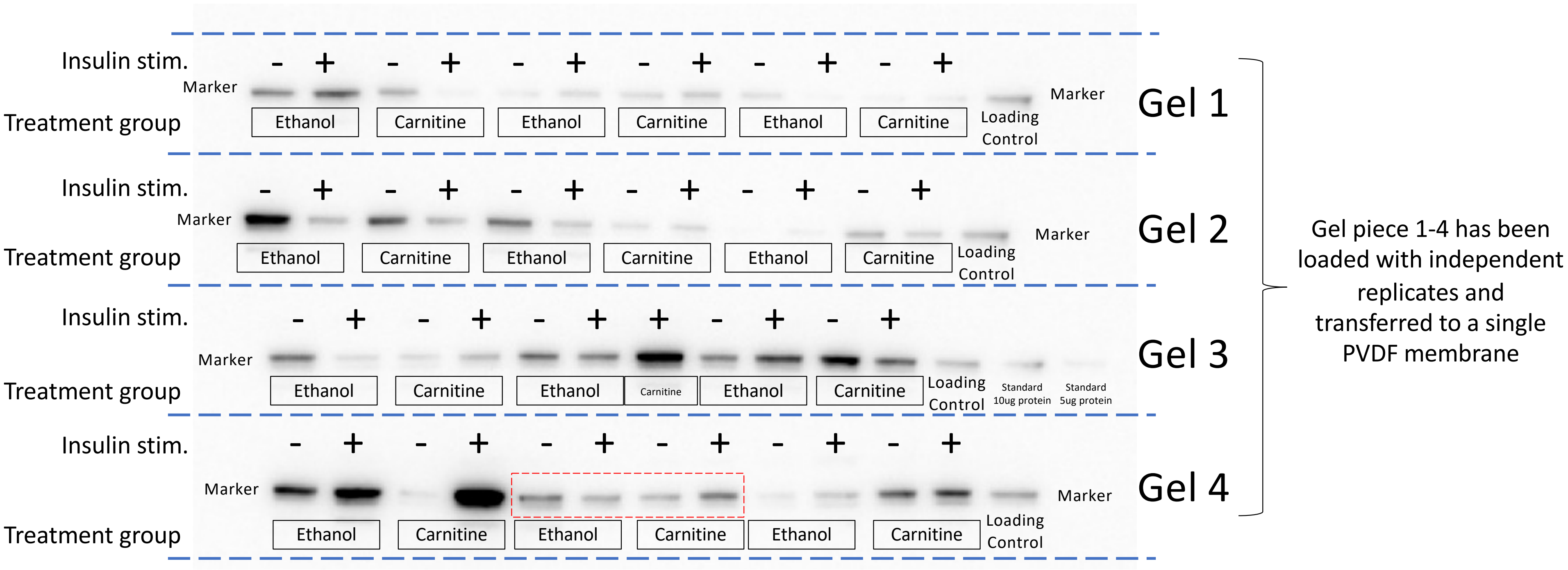

Full unedited gel for Figure 10
P-Akt Thr308

Red and green dashed line box indicate representative western blot shown in article
The representative western blot within the green dashed box was horizontally flipped in the article to align with the band order as presented.

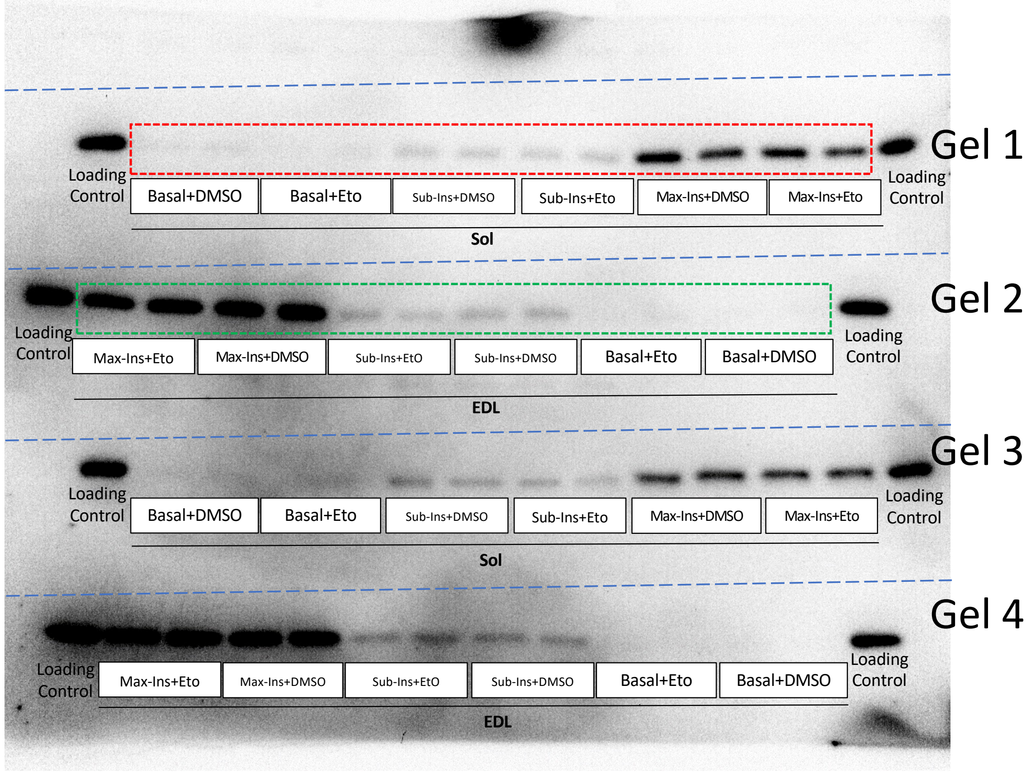

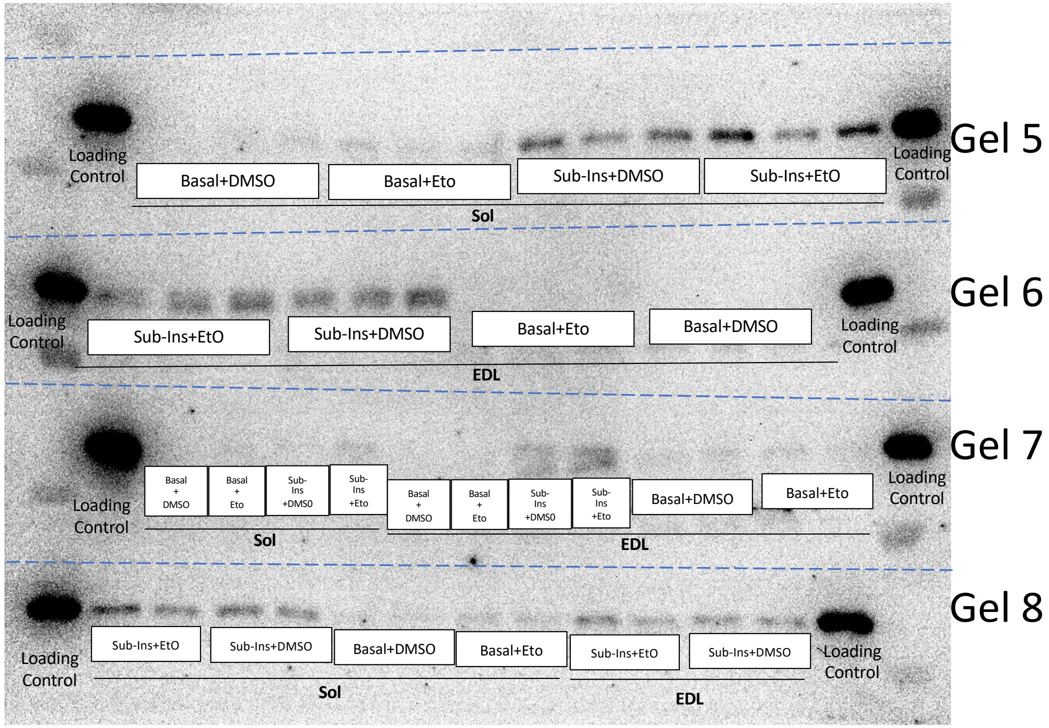

Gel piece 1-8 has been loaded with independent replicates and transferred onto two separate PVDF membranes

Full unedited gel for Figure 10
P-Akt Ser473

Red and green dashed line box indicate representative western blot shown in article
The representative western blot within the green dashed box was horizontally flipped in the article to align with the band order as presented.

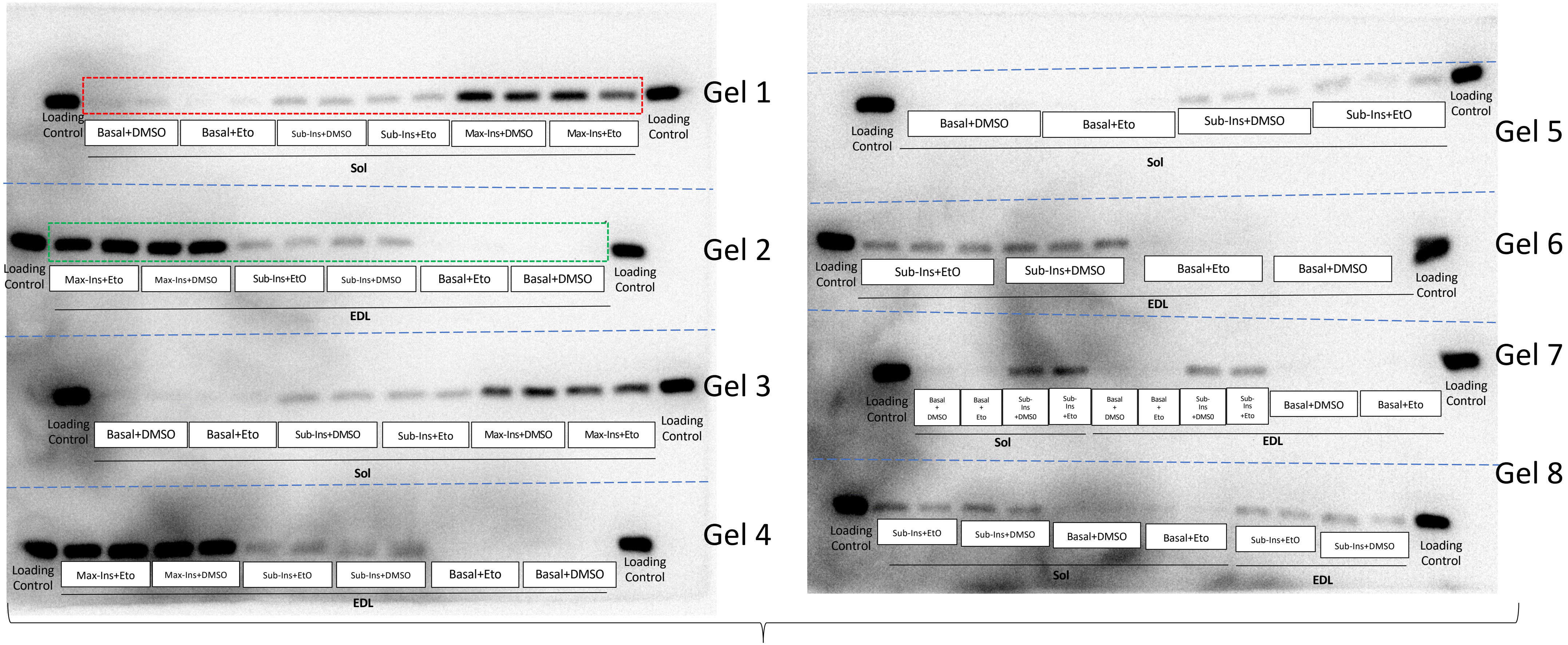

Gel piece 1-8 has been loaded with independent replicates and transferred onto two separate PVDF membranes

Full unedited gel for Figure 10
T-Akt

Red and green dashed line box indicate representative western blot shown in article
The representative western blot within the green dashed box was horizontally flipped in the article to align with the band order as presented.

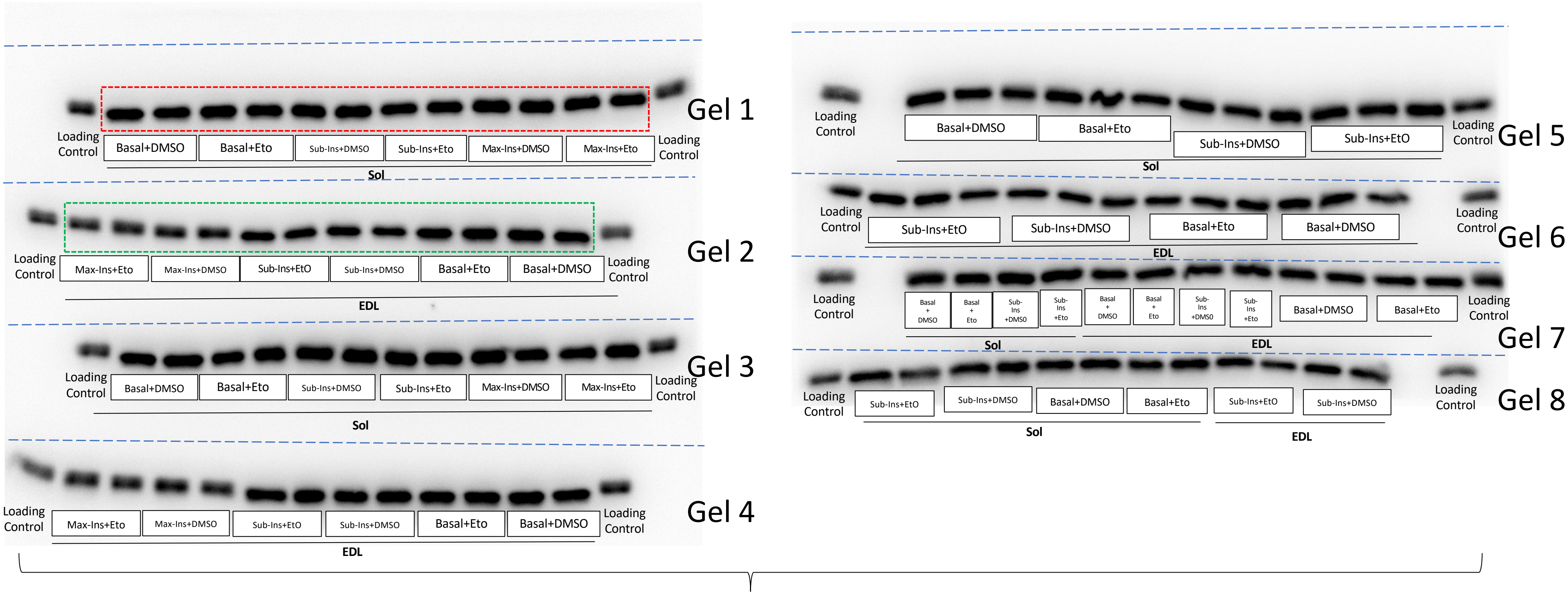

Gel piece 1-8 has been loaded with independent replicates and transferred onto two separate PVDF membranes

Full unedited gel for Supplemental Fig.6
GLUT4

Red dashed line box indicates representative western blot shown in article.

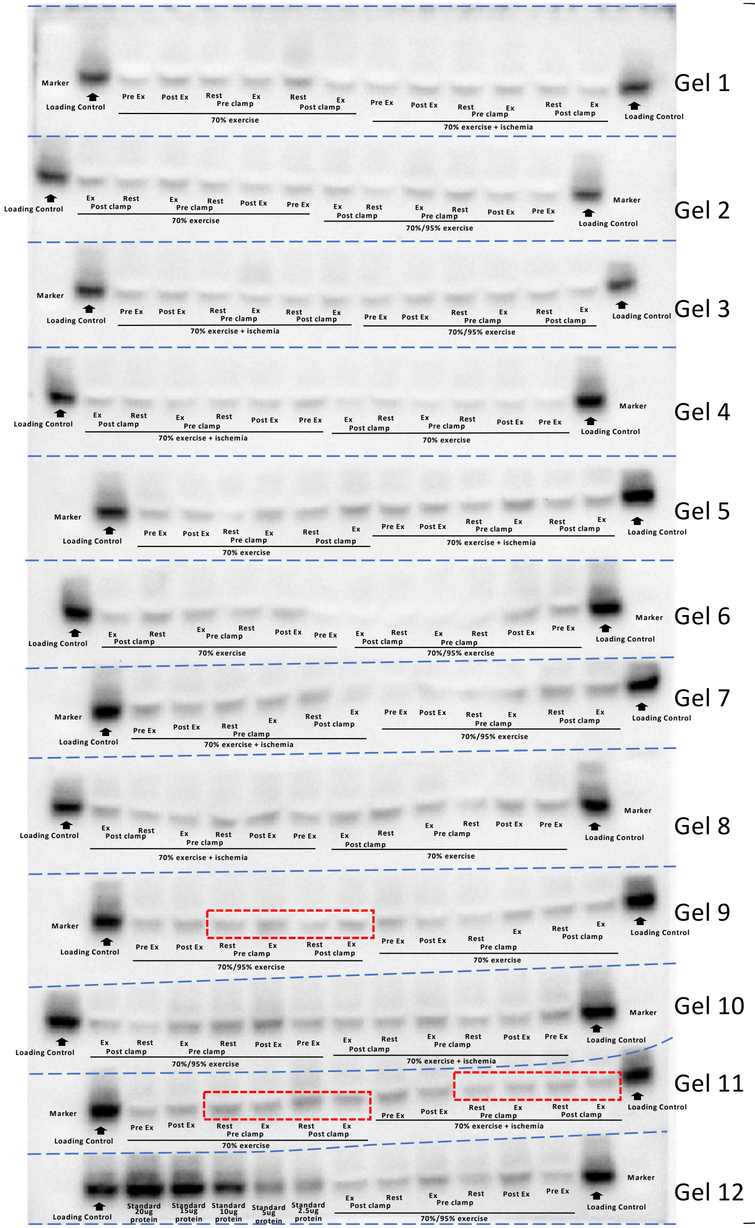

Gel piece 1-12 has been loaded with independent replicates and transferred to a single PVDF membrane

Full unedited gel for Supplemental Fig.6 HKI

Red and green dashed line box indicate representative western blot shown in article. The representative western blot within the green dashed box was horizontally flipped in the article to align with the band order as presented.

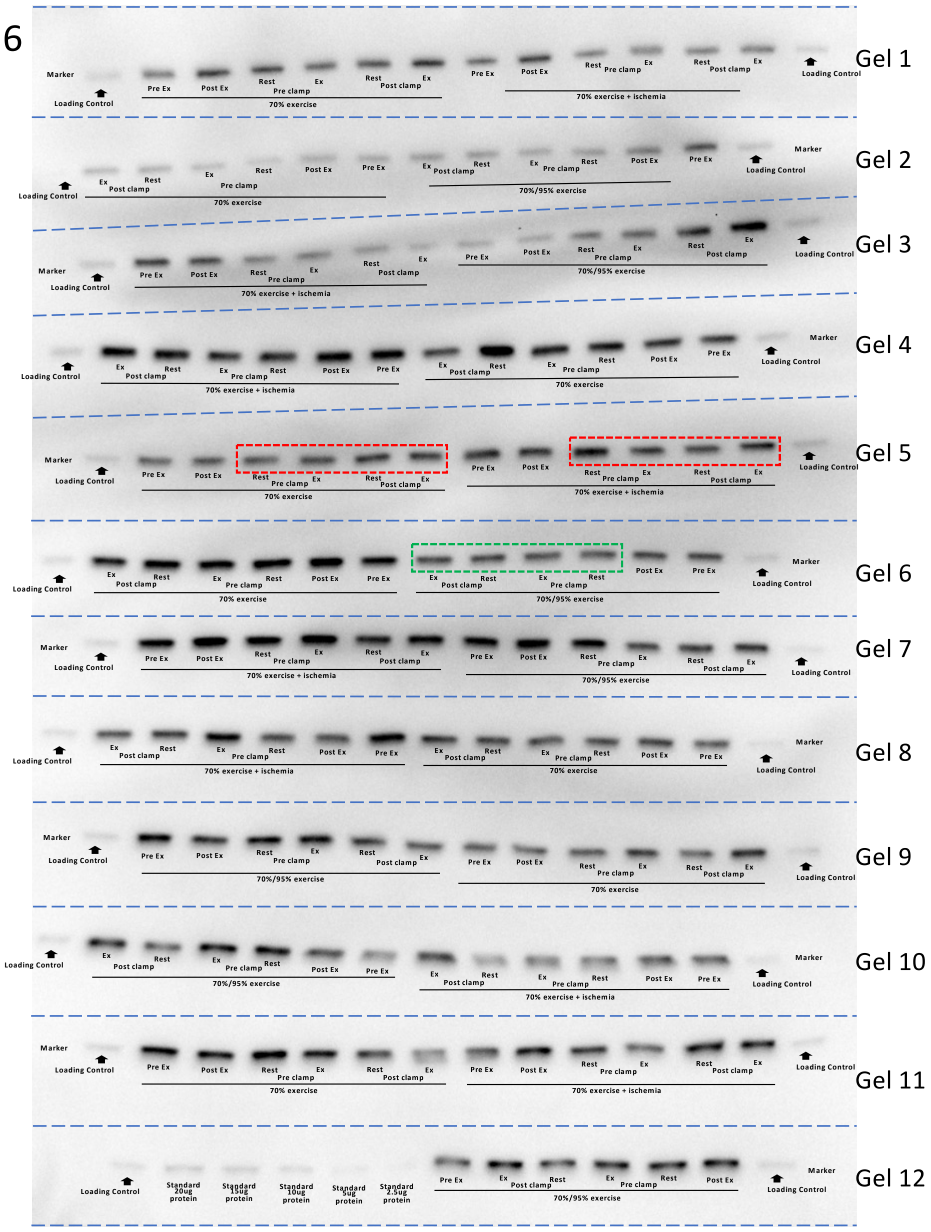

Gel piece 1-12 has been loaded with independent replicates and transferred to a single PVDF membrane

Full unedited gel for Supplemental Fig.6
HKII

Red dashed line box indicates representative western blot shown in article.

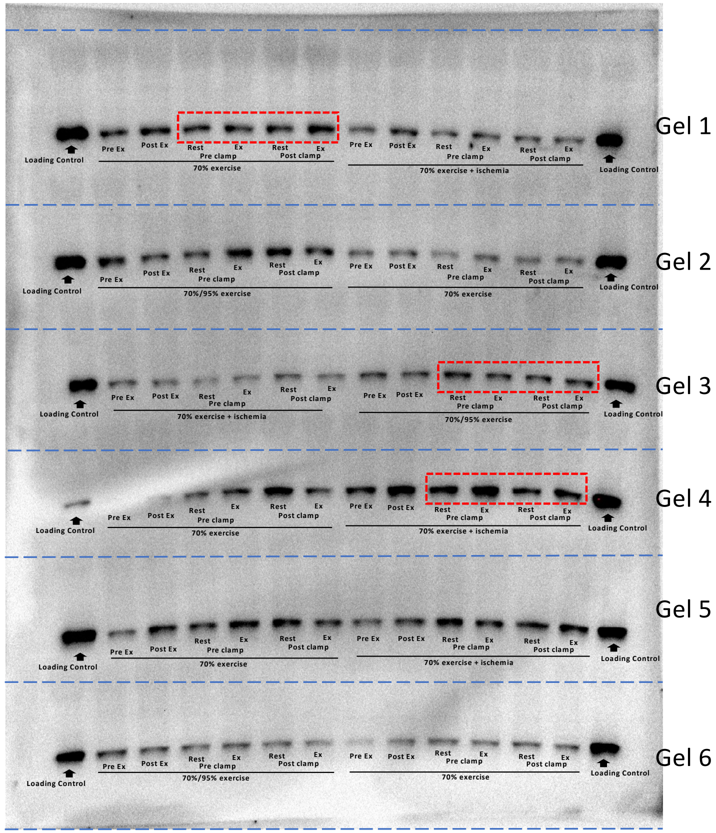

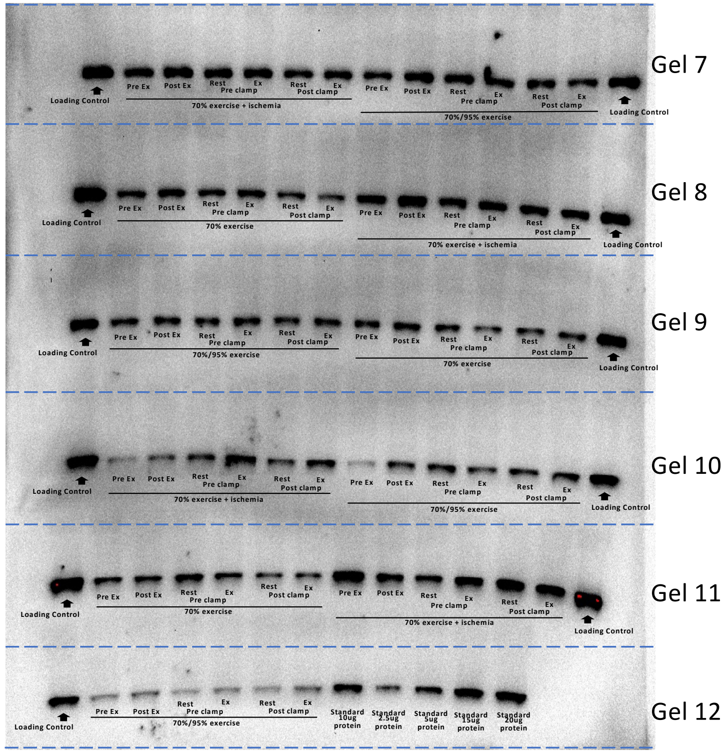

Gel piece 1-12 has been loaded with independent replicates and transferred onto two separate PVDF membranes

Full unedited gel for Supplemental Fig.6
T-GS

Red dashed line box indicates representative western blot shown in article.

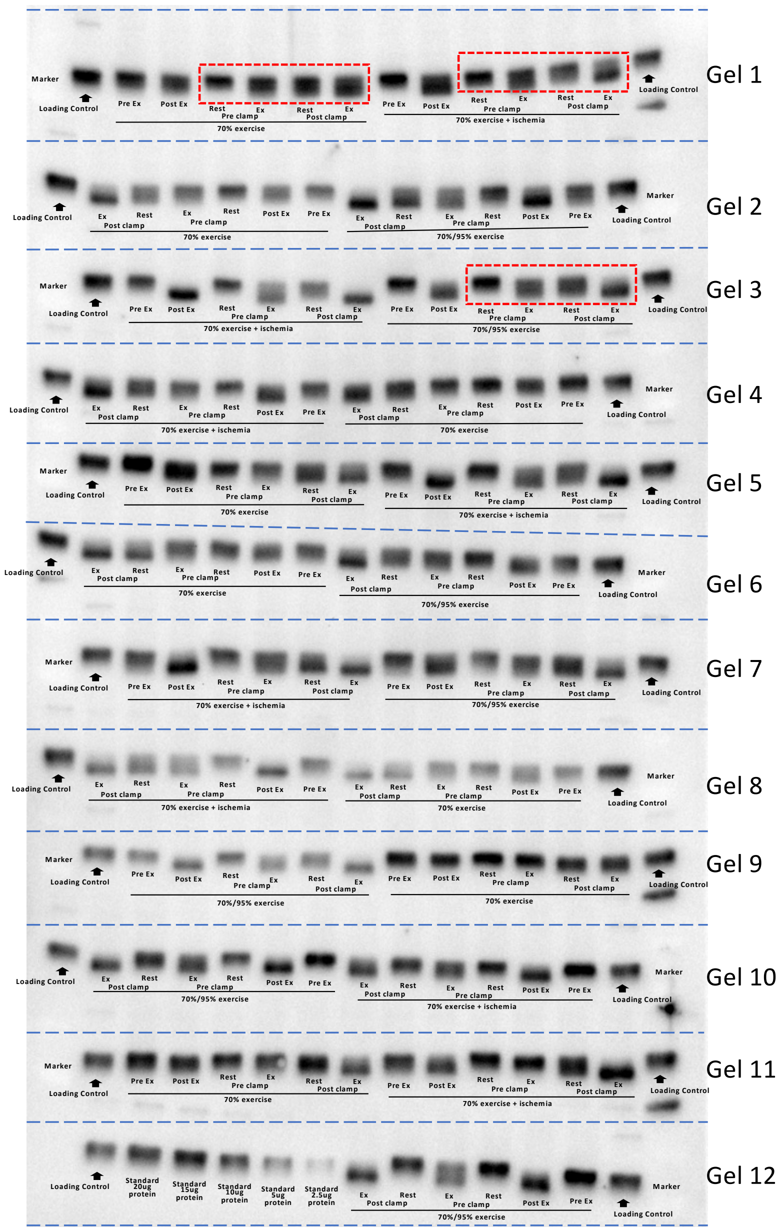

Gel piece 1-12 has been loaded with independent replicates and transferred to a single PVDF membrane

Full unedited gel for Supplemental Fig.6
T-PDH

Red dashed line box indicates representative western blot shown in article

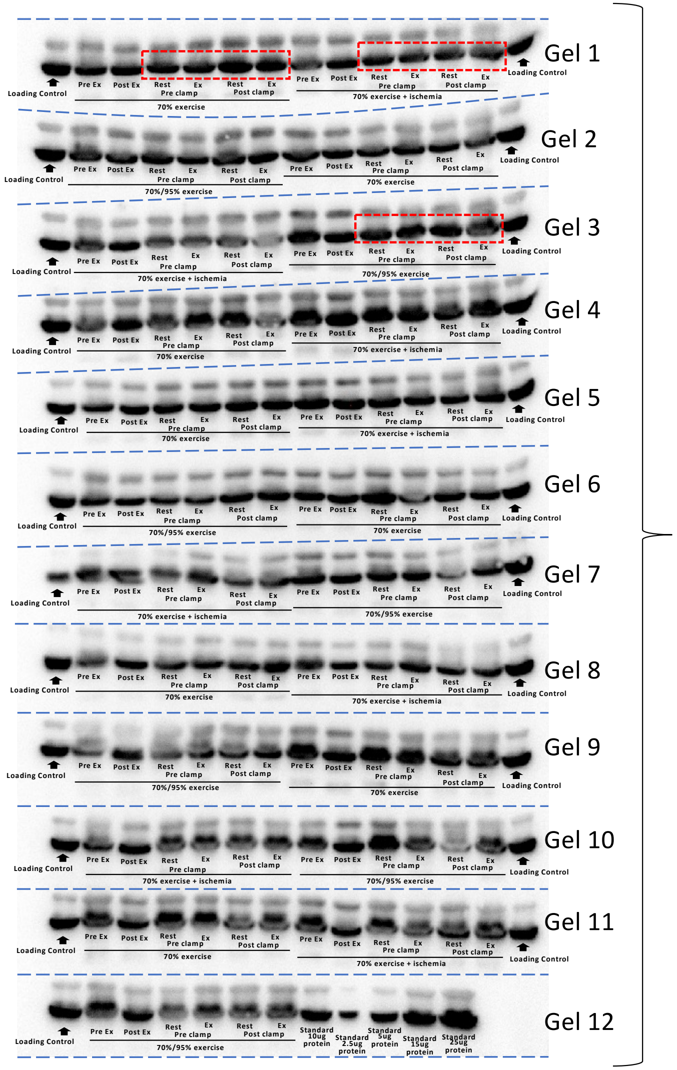

Gel piece 1-12 has been loaded with independent replicates and transferred to a single PVDF membrane

Full unedited gel for Supplemental Fig.7
T-AMPK

Red and green dashed line box indicate representative western blot shown in article.
The representative western blot within the green dashed box was horizontally flipped in the article to align with the band order as presented.

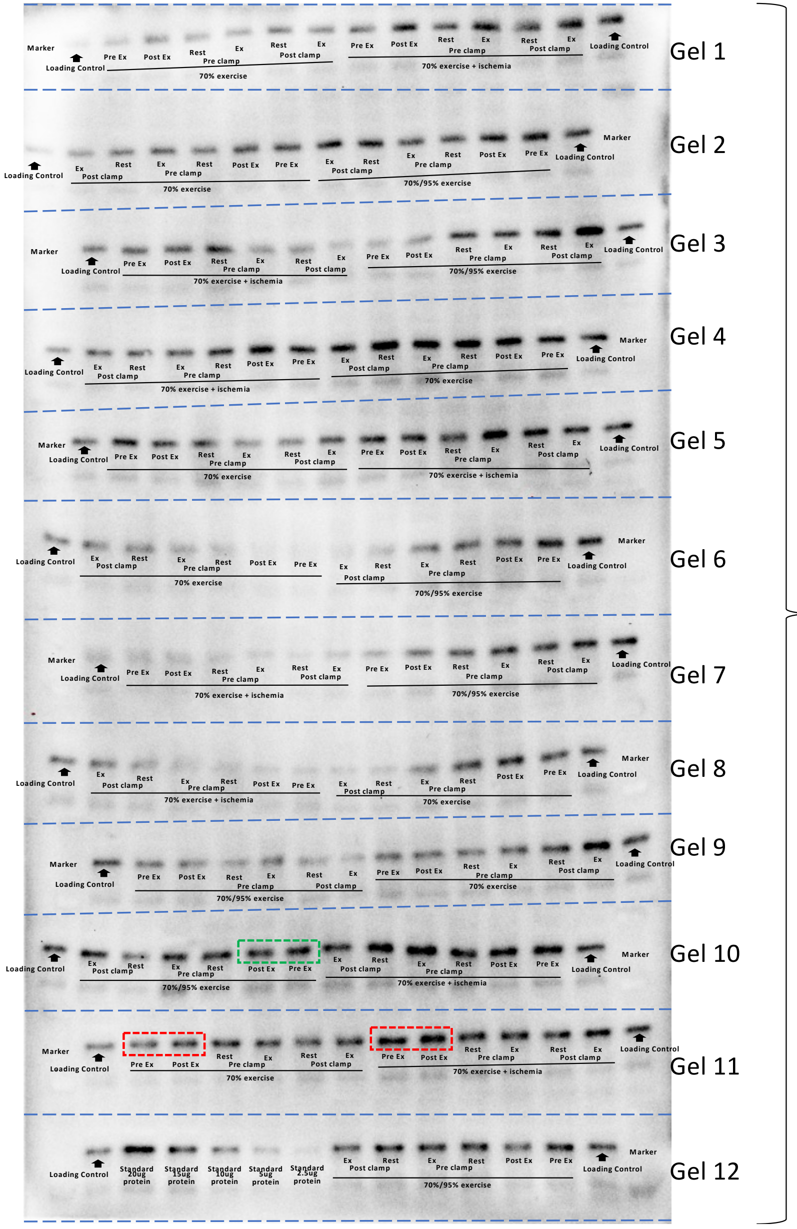

Gel piece 1-12 has been loaded with independent replicates and transferred to a single PVDF membrane

Full unedited gel for Supplemental Fig.7

T-ACC

Red and green dashed line box indicate representative western blot shown in article
The representative western blot within the green dashed box was horizontally flipped in the article to align with the band order as presented.

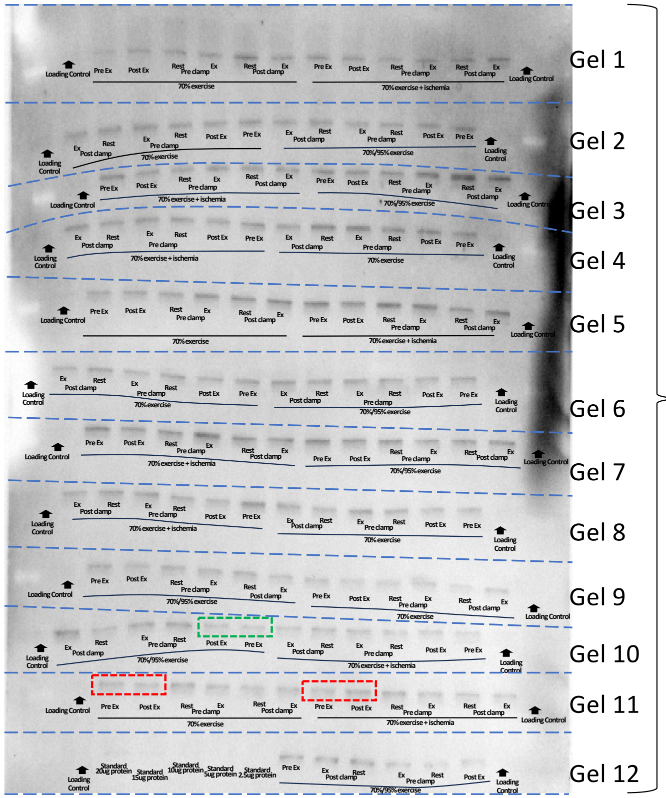

Gel piece 1-12 has been loaded with independent replicates and transferred to a single PVDF membrane

Full unedited gel for
Supplemental Fig.7
T-TBC1D1

Red dashed line box indicates representative western blot shown in article

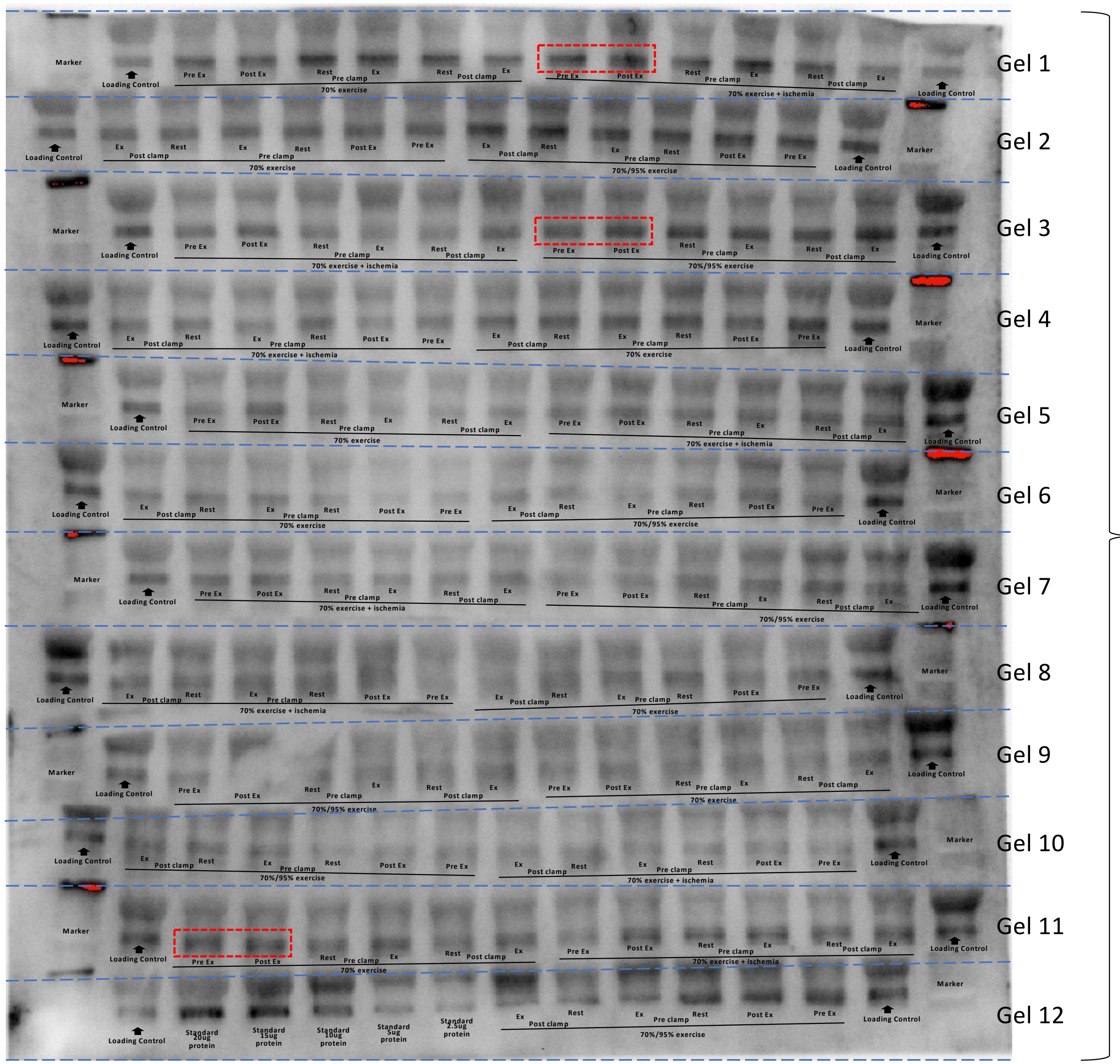

Gel piece 1-12 has been loaded with independent replicates and transferred to a single PVDF membrane

Full unedited gel for Supplemental Fig.7
T-TBC1D4

Red dashed line box indicates representative western blot shown in article

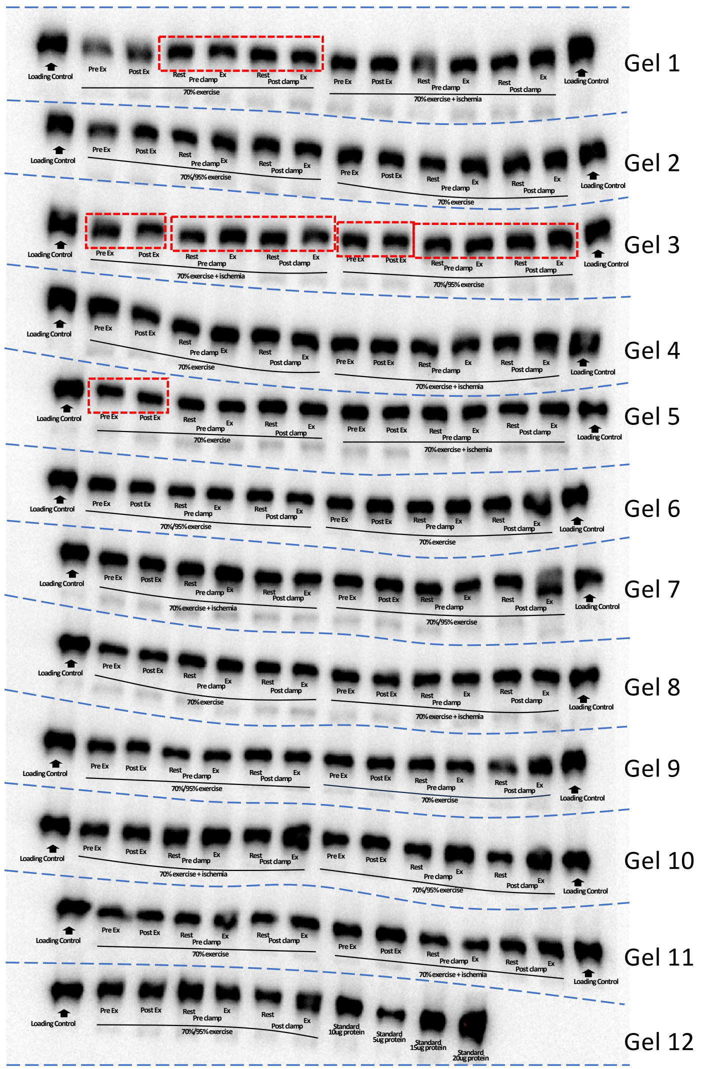

Gel piece 1-12 has been loaded with independent replicates and transferred to a single PVDF membrane

Full unedited gel for Supplemental Fig.7
T-Akt

Red dashed line box indicates representative western blot shown in article

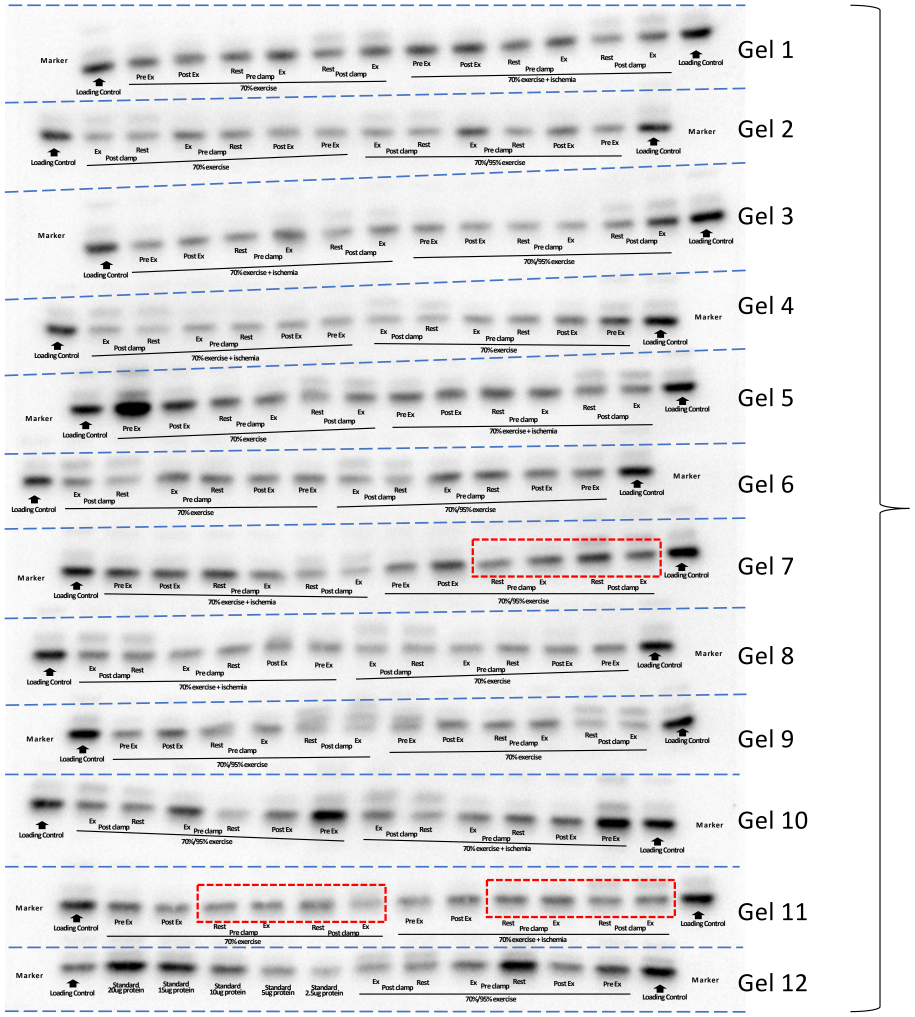

Gel piece 1-12 has been loaded with independent replicates and transferred to a single PVDF membrane
